# Enzymatic Conversion of CO_2_: From Natural to Artificial Utilization

**DOI:** 10.1021/acs.chemrev.2c00581

**Published:** 2023-01-24

**Authors:** Sarah Bierbaumer, Maren Nattermann, Luca Schulz, Reinhard Zschoche, Tobias J. Erb, Christoph K. Winkler, Matthias Tinzl, Silvia M. Glueck

**Affiliations:** † Institute of Chemistry, University of Graz, NAWI Graz, Heinrichstraße 28, 8010 Graz, Austria; ‡ Department of Biochemistry and Synthetic Metabolism, Max Planck Institute for Terrestrial Microbiology, Karl-von-Frisch Straße 10, 35043 Marburg, Germany; § BASF SE, Carl-Bosch-Straße 38, 67056 Ludwigshafen am Rhein, Germany

## Abstract

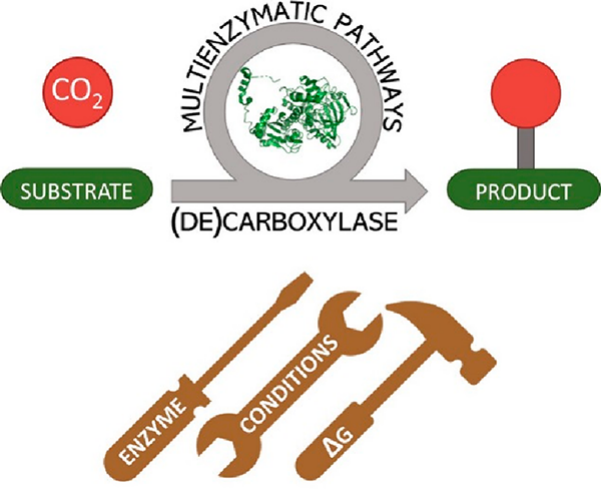

Enzymatic carbon dioxide fixation is one of the most important metabolic reactions as it allows the capture of inorganic carbon from the atmosphere and its conversion into organic biomass. However, due to the often unfavorable thermodynamics and the difficulties associated with the utilization of CO_2_, a gaseous substrate that is found in comparatively low concentrations in the atmosphere, such reactions remain challenging for biotechnological applications. Nature has tackled these problems by evolution of dedicated CO_2_-fixing enzymes, i.e., carboxylases, and embedding them in complex metabolic pathways. Biotechnology employs such carboxylating and decarboxylating enzymes for the carboxylation of aromatic and aliphatic substrates either by embedding them into more complex reaction cascades or by shifting the reaction equilibrium via reaction engineering. This review aims to provide an overview of natural CO_2_-fixing enzymes and their mechanistic similarities. We also discuss biocatalytic applications of carboxylases and decarboxylases for the synthesis of valuable products and provide a separate summary of strategies to improve the efficiency of such processes. We briefly summarize natural CO_2_ fixation pathways, provide a roadmap for the design and implementation of artificial carbon fixation pathways, and highlight examples of biocatalytic cascades involving carboxylases. Additionally, we suggest that biochemical utilization of reduced CO_2_ derivates, such as formate or methanol, represents a suitable alternative to direct use of CO_2_ and provide several examples. Our discussion closes with a techno-economic perspective on enzymatic CO_2_ fixation and its potential to reduce CO_2_ emissions.

## 1 Introduction

Over billions of years, nature has evolved extremely powerful tools to use CO_2_ as a building block for the formation of biomass. In fact, CO_2_ is likely the most ancient carbon source on the planet. In the course of evolution, all three kingdoms of life have evolved and/or acquired carboxylases that allow them to utilize CO_2_ via different mechanistic pathways. Humankind has recently started to exploit these biocatalysts and concepts for biotechnological and synthetic purposes.^1−5^ In such applications, CO_2_ represents an attractive alternative to building blocks derived from fossil feedstock. As a result, carboxylases as well as decarboxylases from both primary and secondary metabolism of various microbial sources have been used for synthetic CO_2_ utilization.^6−14^

Herein, we provide an overview of carboxylating enzymes that occur in nature and biocatalysts that have potential for biotechnological applications. We discuss and guide the implementation of single enzymes in highly complex artificial pathways and cascades. Furthermore, we highlight the biotechnological potential of other one-carbon (C1) metabolites derived from CO_2_ and give a techno-economic perspective of the field.

In the first section of the review, we provide a general introduction to enzymatic carboxylation reactions by discussing their chemical and energetic aspects. We thereby identify common mechanistic strategies employed by carboxylases to overcome the reaction’s thermodynamic challenges (section 2). The following section (section 3) is focused on illustrating the structural and mechanistic details of natural carboxylases, and (de)carboxylases, which can be employed as carboxylases by reversing the direction of the reaction. Additionally, we provide a summary of strategies that are applied to increase the productivity of in vitro single-enzyme carboxylation systems for the synthesis of chemicals. We then turn our attention from single enzymes to CO_2_-fixing (autotrophic) pathways (section 4). Following a brief overview of naturally occurring autotrophic carbon fixation pathways, we provide a roadmap for the design and realization of new-to-nature CO_2_ fixation pathways. The section closes with an overview of carboxylation cascades that utilize CO_2_ as a synthetic C1 building block and directly aim for a product of synthetic or chemical value. section 5 further extends the concept of enzymatic carbon fixation toward C1 compounds (formate, formaldehyde, methanol) that can be generated through the reduction of CO_2_. The current state of carbon fixation technology and the necessary next technological advances are summarized in section 6 from an industrial perspective. Finally, section 7 highlights the immediate challenges and the exciting opportunities for enzymatic carbon fixation.

As this review aims at providing an integrated view on enzymatic carbon fixation, we realize that our discussions are necessarily incomplete. For further details and insights into selected topics, we want to refer the reader to a number of excellent recent reviews on carboxylating enzymes,^6,9,12^ synthetic applications of carboxylases,^10,11^ engineering of artificial fixation pathways,^15^ as well as thermodynamic considerations.^16^

## 2 Enzymatic CO_2_ Fixation Mechanisms and Thermodynamic Considerations

**Scheme 1:**
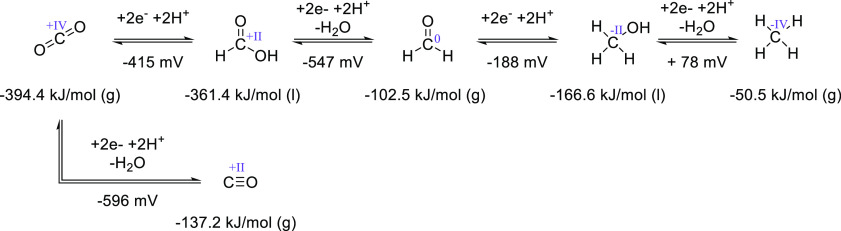
Standard Molar Gibbs Energy of Formation (Δ*fG*°) and Experimental Reduction Potentials of Carbon Dioxide, Carbon Monoxide, Formic Acid, Formaldehyde, Methanol, and Methane, at 298.15 K in kJ/mol (Oxidation States of Carbon Are Given As Roman Numerals)^19,20^

From a chemical perspective, carboxylation reactions are intriguing, as they allow introducing C1 units as building blocks into a target molecule, starting from the abundant and easily accessible carbon source, CO_2_.^10,17^ However, the chemical utilization of CO_2_ as a building block comes with a number of challenges. Most importantly, carbon in CO_2_ is in its highest oxidation state, and therefore any functionalization must be reductive. As was recently put, CO_2_ is at the “bottom of the potential energy well”,^18^ and energy must be invested to utilize it. This is nicely illustrated by the low Gibbs energy of formation of CO_2_ compared to other C1 molecules with a more reduced carbon center, as well as by its comparably low reduction potential (Scheme 1).^19,20^

Although CO_2_ is abundant in the atmosphere, current concentrations (about 412 ppm in 2021^21^) do not allow direct use of atmospheric air for carboxylation chemistry. Additionally, when a gas is used as reagent in synthesis, its phase transfer into solution and its solubility (up to 1.7 g L^–1^ at atmospheric pressure and 20 °C), i.e., the availability of the building block in solution, need to be considered. As CO_2_ undergoes hydration in aqueous solution, CO_2_ availability is strongly pH-dependent, with bicarbonate (HCO_3_^–^) being the most prevalent form at neutral to alkaline pH.

Biocatalytic carboxylation is applied to overcome the mentioned obstacles.^3,5,22^ The high efficiency of enzymes allows acceleration of challenging reactions and the substrate affinity of enzymes permits nature to operate them under the low CO_2_ concentrations in air.^10−12,14,15^ An impressive example of the catalytic efficiency of enzymes that utilize CO_2_ are carbonic anhydrases which belong to the fastest catalysts known to date and accelerate the equilibration of bicarbonate and CO_2 (aq)_ by 6 orders of magnitude.^23^ Evolution has provided a broad portfolio of carboxylases that exhibit different binding and activation modes of CO_2_ and different attack modes of the carbon nucleophile onto the respective electrophilic CO_2_ species. The required electrons for the reduction of CO_2_ are either provided by the substrate itself or in form of reduced cofactors, in particular, nicotinamides (NAD(P)H) or ferredoxin.

### 2.1 General Mechanistic Steps of Enzymatic Carboxylation Reactions

**Scheme 2:**
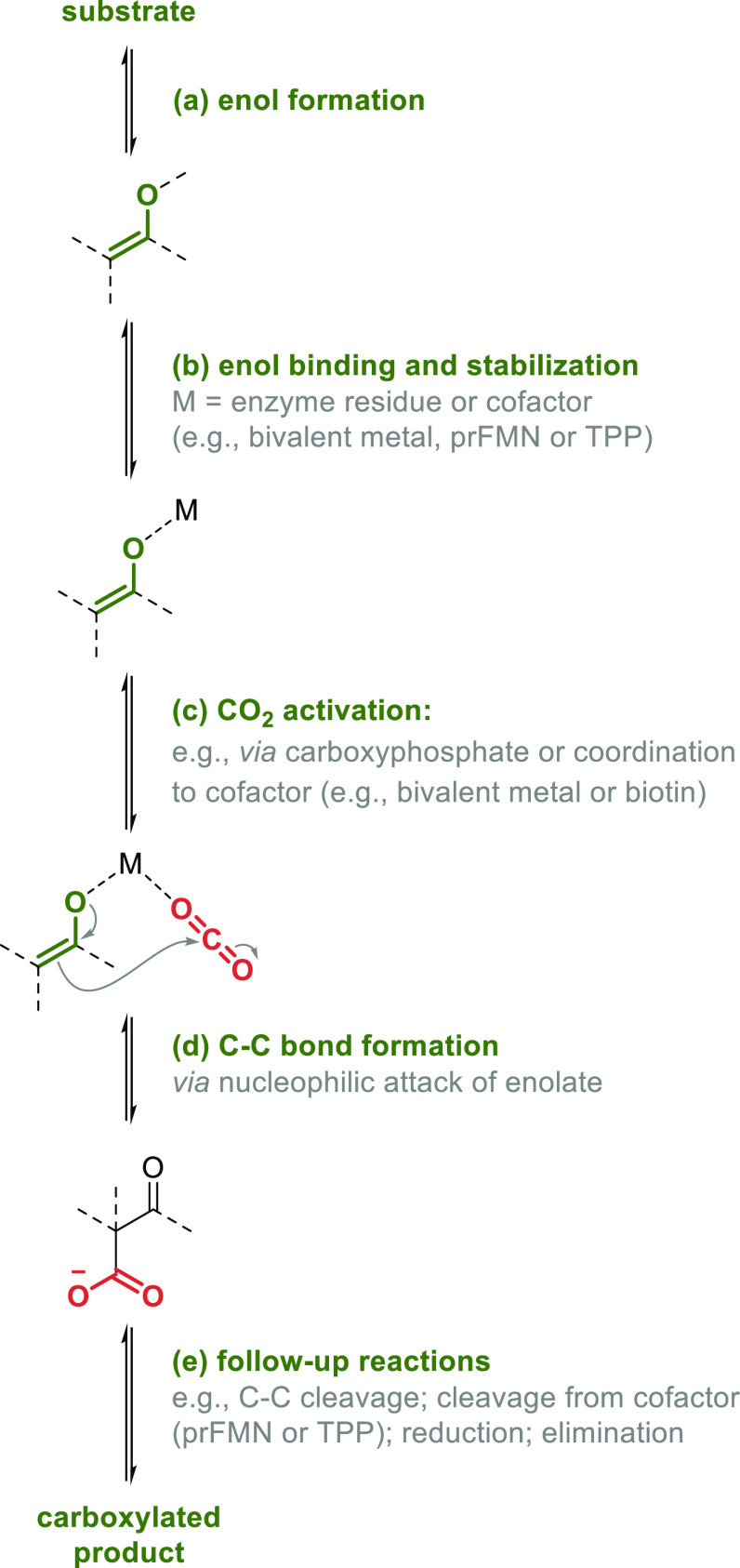
General Mechanistic Steps of Enzymatic Carboxylations

The general mechanistic steps of enzymatic carboxylations are highly similar for almost all carboxylases:^9,12^ (a) generation of an enol or enolate to create a nucleophile, (b) binding and stabilization of the enol (or enolate), (c) accommodation and/or activation of CO_2_ (e.g., as carboxyphosphate), (d) C–C bond formation via nucleophilic attack of the enol (or enolate) onto the carbon of CO_2_, and finally (e) potential follow up reactions such as cleavage of the product from the cofactor (Scheme 2).

Enzymes that do not follow these general steps are the pyridoxal-5′-phosphate (PLP)-dependent glycine cleavage system (GCS)^24^ that is discussed in section 3.1.2, the ferulic acid decarboxylases that depend on a novel prenylated FMN cofactor to catalyze the reversible decarboxylation of ferulic acids via a 1,3-cycloaddition^25^ (compare section 3.2.3), and enzymes that directly reduce CO_2_, such as CO dehydrogenase^26^ or formate dehydrogenase^27^ (compare section 3.1.2). Below, we discuss the key steps for bio(techno)logically relevant carboxylases in detail, with a focus on (step a) enol formation and (step c) CO_2_ activation. Note that for reversible decarboxylases, carboxylation is herein interpreted as the reversed decarboxylation mechanism. For details on specific mechanisms, refer to the subsections of the respective enzyme family (section 3).

**Scheme 3:**
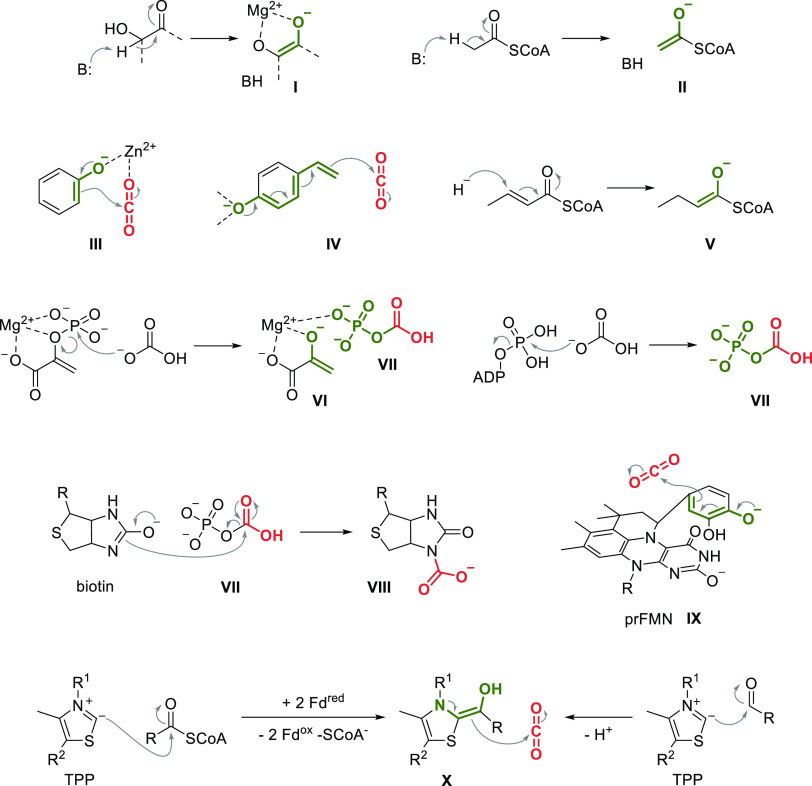
Mechanistic Strategies for the Activation of the Nucleophile (Enol- or Enamine-Formation) and CO_2_ (I) Endiolate stabilized by Mg^2+^, formed in the mechanism of RuBisCO. (II) Enolate formed by acetyl-CoA carboxylases. (III) Phenolate bound to Zn^2+^ in the active site of bivalent metal-dependent decarboxylases. (IV) Trienolate attack onto CO_2_ by the cofactor-independent decarboxylases. (V) Hydride attack (NADPH) onto crotonyl-CoA by enoyl-CoA carboxylases/reductases, forming the intermediary enolate. (VI) Cleavage of the phosphate ester of phosphoenolpyruvate by pyruvate carboxylase. (VII) Carboxyphosphate, formed either from phosphoenolpyruvate or ATP and bicarbonate. (VIII) Formation of carboxybiotin by acetyl-CoA carboxylases. (IX) Intermediary dienolate, covalently bound to prFMN, in the mechanism of AroY-type enzymes. (X) Umpolung of the carbonyl center of either acyl-CoA (2-ketoglutarate synthase or pyruvate synthase) or aldehyde substrates (pyruvate decarboxylase) with TPP as cofactor.

Enols, enolates, and enamines serve as carbon nucleophiles, not only in synthetic organic chemistry but also in biochemistry. It is therefore no surprise that nature has evolved a range of different ways for their formation. The most straightforward reaction to form an enolate proceeds via α-deprotonation of carbonyls. For example, in the reaction catalyzed by RuBisCO, the hydroxy-keto moiety of d-ribulose-1,5-bisphosphate is deprotonated, forming an enediolate, which is stabilized by the active site Mg^2+^ (Scheme 3, I, see also section 3.1.1).^28^ Similarly, enolates are formed via deprotonation in isocitrate dehydrogenases and acetyl-CoA carboxylases, which feature an acetyl-CoA anion (Scheme 3, II) that eventually attacks carboxybiotin (VIII, as discussed below; see also section 3.1.2). Also, the reversed reaction of bivalent metal-dependent decarboxylases (section 3.2.1) and the cofactor-independent decarboxylases (section 3.2.2) require initial activation of the substrate via deprotonation of the substrate’s phenolic hydroxy group. In the first case, the phenolate is bound by a bivalent metal ion in the active site and the ortho-carbon, possessing the character of an enolate, attacks CO_2_ (Scheme 3, III, Zn^2+^ can also be Mg^2+^ or Mn^2+^).^29,30^ For cofactor-independent decarboxylases, the reaction proceeds via a quasi-trienolate (Scheme 3, IV).^31^ A mechanistic alternative for enolate formation is the 1,4-addition of a hydride from NADPH to α,β-unsaturated CoA esters, as in the case of enoyl-CoA carboxylases/reductases, such as crotonyl-CoA carboxylase/reductase (Scheme 3, V; compare to section 3.1.2).^32^

In the case of phosphoenolpyruvate carboxylase (PEPC, see also section 3.1.1), the substrate itself (phosphoenolpyruvate) is already an enol-ester, and, upon cleavage of the phosphoester group with bicarbonate, the enolate is released (stabilized by an active site Mg^2+^; Scheme 3, VI). In parallel, this reaction also activates CO_2_ as the mixed anhydride carboxyphosphate which serves as carboxylation source (Scheme 3, VII).^33^

A similar activation of CO_2_ as carboxyphosphate is also a key-step in other carboxylation mechanisms, such as the reaction catalyzed by acetyl-CoA carboxylases (see also section 3.1.1). In the first mechanistic step, these enzymes convert bicarbonate in an ATP-dependent reaction into carboxyphosphate (Scheme 3, VII). This is followed by the formation of carboxybiotin, another activated CO_2_ equivalent (Scheme 3, VIII) which in turn facilitates the carboxylation of an acetyl-CoA enolate (Scheme 3, II).^34^

Enzymes that depend on prenylated FMN (prFMN) as cofactor show a range of different carboxylation mechanisms (see section 3.2.3). The *para*-carboxylation of phenolic substrates proceeds via a substrate that is covalently bound to prFMN via a quasi-dienolate (Scheme 3, IX).^35^

Finally, the mechanism of enzymes that depend on thiamin pyrophosphate (TPP) differs from the above-mentioned cases, as the nucleophile is not an enolate but an enamine. In the case of 2-ketoglutarate:ferredoxin oxidoreductase or pyruvate:ferredoxin oxidoreductase, the carbonyl of the respective acyl-CoA is turned into a nucleophile via an Umpolung. The reaction requires two electrons, provided by ferredoxins, leading to formation of a TPP-bound enamine intermediate that can attack CO_2_ (Scheme 3, X; compare also section 3.1.2).^36−38^ The same intermediate is produced from aldehydes by pyruvate decarboxylase, however, in this case no reduction is required (compare to section 3.2.4).^39^

### 2.2 Thermodynamic Point of View

**Scheme 4:**
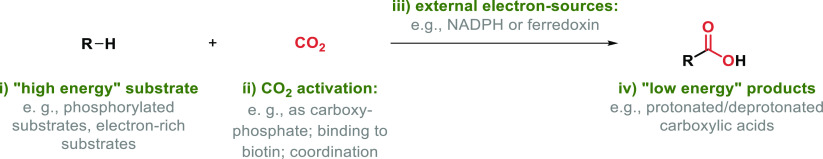
Nature’s Strategies for Reducing the Free Energy of Carboxylation Reactions

For thermodynamically controlled reactions, such as carboxylations, the change in Gibbs free energy (Δ*G*) is a key parameter that needs to be considered (in contrast to kinetically controlled reactions, where the individual reaction rates are more relevant).^40^ In nature and synthesis, carboxylation reactions are accomplished either by reducing the required reaction energy (Δ_r_*G* or the activation energy) or by shifting the reaction equilibrium using Le Chatelier’s principle.^14^ Nature embeds carboxylations within metabolic pathways that are exergonic (cf. section 4.1). The same principle was applied for the development of artificial CO_2_–fixing enzyme cascades (cf. section 4.2). Besides shifting the equilibrium, the energy demand^41−43^ of the overall reaction is reduced by several strategies that are discussed below (Scheme 4).^12,14^ Note that the Δ_r_*G*° values given in this section were calculated using the eQuilibrator tool^42,43^ at standard conditions (1 M concentration of the involved species, 25 °C and 1 bar; and at pH 7.5 and an ionic strength of 0.25 M) and compared to values reported in literature, whenever possible.^14,16^

Strategies to render carboxylation reactions more favorable include:

(i) Using high energy starting materials: This is the case for many CO_2_-fixing enzymes that do not depend on external reductants. Examples are the reactions catalyzed by RuBisCO^44^ or PEPC.^45^ Both reactions are energetically favored, with a Δ_r_*G*° of −32.2 kJ/mol for PEPC^16,42^ and Δ_r_*G*° = −32.0 kJ/mol for RuBisCO, which in addition benefits from the energy provided by the hydrolysis of the carboxylation product, i.e., from the fact that two “low energy” products are formed (see section 3.1.1).^16,42^

(ii) Activation of CO_2_: As previously discussed, many structurally distinct carboxylases share the mechanistic feature of CO_2_ coordination to a bivalent metal in the active site (Mg^2+^, Zn^2+^, or Mn^2+^). Examples include PEPC, isocitrate dehydrogenase, or the metal-dependent phenolic acid decarboxylases.^11,12^ Furthermore, CO_2_ (or HCO_3_^–^) is often directly activated by ATP, forming the reactive carboxyphosphate. Hydrolysis of one phosphate group of ATP provides between 26 and 43 kJ/mol, depending on the Mg^2+^ concentration.^16,42^ Examples include the biotin-dependent acetyl-CoA and propionyl-CoA carboxylases. The free energy of the ATP-dependent enzymatic carboxylation of acetyl-CoA, for example, sums up to −9.1 kJ/mol.^16,42^ Catalysis proceeds via the formation of carboxyphosphate and the carboxylation of biotin (see also section 3.1.1).

(iii) Supply of external reducing equivalents: Electron donors that are commonly utilized are either NADPH, providing up to approximately 65 kJ/mol (for two electrons), or ferredoxins, worth up to approximately 40 kJ/mol per electron.^16,19^ 2-Ketoglutarate synthases, which utilize the TPP cofactor to carboxylate CoA thioesters forming α-keto acids, are examples of ferredoxin-dependent reductive carboxylases. Crotonyl-CoA carboxylase/reductases and isocitrate dehydrogenases depend either on NADPH or NADH and are energetically favorable with Δ_r_*G*° = −14.2 kJ/mol and Δ_r_*G*° = −5.4 kJ/mol, respectively. However, at millimolar substrate concentrations the isocitrate dehydrogenase reaction becomes unfavorable. The glycine cleavage system requires additional energy to run in the carboxylation direction (Δ_r_*G*° = 3.2 kJ/mol).^16,42^ Propionyl-CoA synthase, an enzyme that catalyzes the conversion of 3-hydroxypropionate to propionyl-CoA, harbors a promiscuous NADPH-dependent carboxylation activity (Δ_r_*G*° = −43 kJ/mol) that was recently enhanced by engineering.^46^ Pyruvate:ferredoxin oxidoreductase can only catalyze the formation of pyruvate from CO_2_ and acetyl-CoA if either reduced cofactor or highly reducing ferredoxins are present in sufficient amounts.^47^ In addition to these carboxylases, enzymes that perform direct reduction of CO_2_, namely formate dehydrogenase and CO-dehydrogenase, also require either NADPH or ferredoxin as electron donor.

(iv) Formation of low energy products: the carboxylation of phenolic compounds, as performed by the enzymes of the UbiX-UbiD family, or by the metal-dependent decarboxylases, yields carboxylic acids.^10−12^ The reaction, however, is endergonic and the experimentally measured free standard energy demand of the reaction of approximately 22 kJ/mol^48^ fits roughly to the calculated value of 15.9 kJ/mol (Δ_r_*G*°; Δ_r_*G*′ = 33.0 kJ/mol at concentrations of 1 mM).^42^ The thermodynamically expected reduction of the reactions free energy demand via increasing the concentration of the CO_2_-donor bicarbonate was experimentally demonstrated.^29^

In (synthetic) metabolic pathways, the overall energy demand is usually not given in kJ/mol but instead is measured as the number of ATP and NADPH equivalents consumed per molecule of CO_2_ fixed.^15^ For example, the Calvin–Benson–Bassham cycle (CBB cycle) requires 8.5 ATP equivalents per equivalent of fixed CO_2_. In comparison, the reductive oxidative citric acid cycle (rTCA cycle) only consumes 5 such equivalents per CO_2_(Table 5). Natural CO_2_ fixation pathways were recently classified as “energy-intensive” if the total change in Gibbs free energy per mol of fixed carbon is more negative than −60 kJ/mol and “energy-saving” if it is less negative.^16^ Interestingly, while pathways from aerobic organisms are thermodynamically favorable, they generally have a high demand of ATP. In contrast, anaerobic organisms tend to resort to pathways which proceed with a lower gain in overall free energy but require less ATP (“energy-saving pathways”). This is most likely due to the ability of aerobic organisms to synthesize ATP via oxidative phosphorylation, thereby lifting ATP efficiency restrictions. To allow a better comparison of the different pathways, herein we normalized their energy demand to acetyl-CoA as final product (see section 4, Table 5 and Supporting Information). However, not only the overall reaction energy is important. For the development of synthetic carbon fixation pathways, the thermodynamic profile of reaction sequences plays an important role, as further discussed in section 4. Note that the recently investigated correlation of the reduction potential with basic reaction types allows a quick identification of the reactions that provide energetic barriers.^20^

## 3 Enzymatic Carboxylation and CO_2_ Utilization Systems

The currently available enzymatic carboxylation systems may be categorized into two classes: carboxylases that perform CO_2_ fixation or modification as their natural function, i.e. RuBisCO and other enzymes that are involved in natural CO_2_ fixation pathways (section 3.1), and decarboxylases that naturally perform the decarboxylation reaction but that can be “tricked” into carboxylation (section 3.2). While enzymes involved in central metabolism are in general highly specific for their respective substrates, decarboxylases, which are mostly part of biodegradation pathways, accept a broad range of substrates.^14^ Building on these carboxylation systems, several reaction parameters can be tuned in order to reach higher conversion or productivity as summarized in section 3.3.

### 3.1 Carboxylases Involved in Natural CO_2_ Fixation and Utilization

CO_2_ is necessarily reduced in any chemical carbon fixation reaction. This reduction requires electrons which can be either provided by an external reductant or by oxidation of the substrate/reaction product itself.^9^ As this difference represents an important mechanistic distinction, we discuss natural CO_2_-fixing enzymes according to these two categories: (i) enzymes which *do not* use external reductants (section 3.1.1) and (ii) enzymes which *do* require external reductants (section 3.1.2). Table 1 provides orientation within the natural carboxylases, highlighting the respective enzyme’s cofactors, its natural role (i.e., the pathway it belongs to), its oxygen-sensitivity, and the source of reducing equivalents.

**Table 1:** Overview of Naturally Occurring Carboxylases^a^

carboxylase	carbon species	cofactor	pathway	O_2_ sensitivity	reducing equivalents/reduced species	ref
ribulose-1,5-bisphosphate carboxylase/oxygenase (RuBisCO)	CO_2_	Mg^2+^	CBB	no, but side reactivity	substrate	(49,50)
phosphoenolpyruvate carboxylase (PEPC)	HCO_3_^–^	Mg^2+^	dicarboxylate	no	substrate	(33,45,51)
acetyl-CoA carboxylase (ACC)	HCO_3_^–^	Mg^2+^, ATP, biotin	3HP bicycle, 3HP/4HB	no	substrate	(34,52)
propionyl-CoA carboxylase (ACPCC)	HCO_3_^–^	Mg^2+^, ATP, biotin	3HP bicycle, 3HP/4HB	no	substrate	(53,54,34,55,56)
2-ketoglutarate:ferredoxin oxidoreductase (2KFOR)	CO_2_	ferredoxin, TPP	rTCA	yes	ferredoxin	(57)
pyruvate:ferredoxin oxidoreductase (PFOR)	CO_2_	ferredoxin, TPP	dicarboxylate cycle	yes	ferredoxin	(36,38,58)
isocitrate dehydrogenase (IDH)	CO_2_	NAD(P)H	rTCA	no	NADPH	(59−61)
glycine cleavage system (GCS)	CO_2_	NAD(P)H, PLP, lipoate	rGlycine	no	NADPH	(24,62)
crotonyl-CoA carboxylase/reductase (CCR)	CO_2_	NAD(P)H	ethylmalonyl-CoA pathway	no	NADPH	(32,63)
formate dehydrogenase (FDH)	CO_2_	ferredoxin, NADH	WL/rGlycine	yes	ferredoxin	(64−66)
CO dehydrogenase (CODH)	CO_2_	Ni4Fe4S	WL	yes	ferredoxin	(67−69)
phosphogluconate dehydrogenase (Gnd)	CO_2_	NADPH	GED	no	NADPH	(70)
pyruvate carboxylase	HCO_3_^–^	Mg^2+^, ATP, biotin	anaplerosis	no	substrate	(71,72)
methylcrotonyl-CoA carboxylase	HCO_3_^–^	Mg^2+^, ATP, biotin	leucine degradation	no	substrate	(34,56)
acetone carboxylase	HCO_3_^–^	Mn^2+^/Zn^2+^	acetone degradation	no	substrate	(73,74)
urea carboxylase	HCO_3_^–^	Mg^2+^, ATP, biotin	urea degradation	no	substrate	(75,76)
vitamin K-dependent carboxylase	CO_2_	vitamin K	glutamate carboxylation	no	vitamin K	(77,78)
phenylphosphate carboxylase	CO_2_	K^+^, Mg^2+^ or Mn^2+^	phenol degradation	yes	substrate	(79,80)

a CBB, Calvin–Benson–Bassham; 3HP, 3-hydroxypropionate; WL, Wood–Ljungdahl; DC, dicarboxylate; 3HP/4HB, 3-hydroxypropionate/4-hydroxybutyrate; rGlycine, reductive glycine; rTCA, reverse tricarboxylic acid cycle; RuBisCO, ribulose-1,5-bisphosphate carboxylase/oxygenase. Carboxylases below the double dashed line are not described in detail.

#### 3.1.1 CO_2_-Fixing Enzymes without External Reductant

##### 3.1.1.1 Ribulose-1,5-bisphosphate Carboxylase/Oxygenase (RuBisCO)

**Scheme 5:**
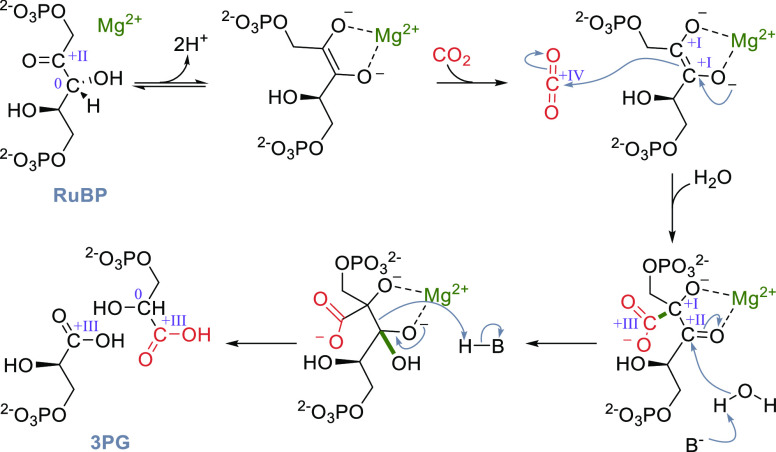
Catalytic Cycle of RuBisCO; Changes of the Oxidation State of Selected Carbons Are Given in Roman Numerals

**Figure 1:**
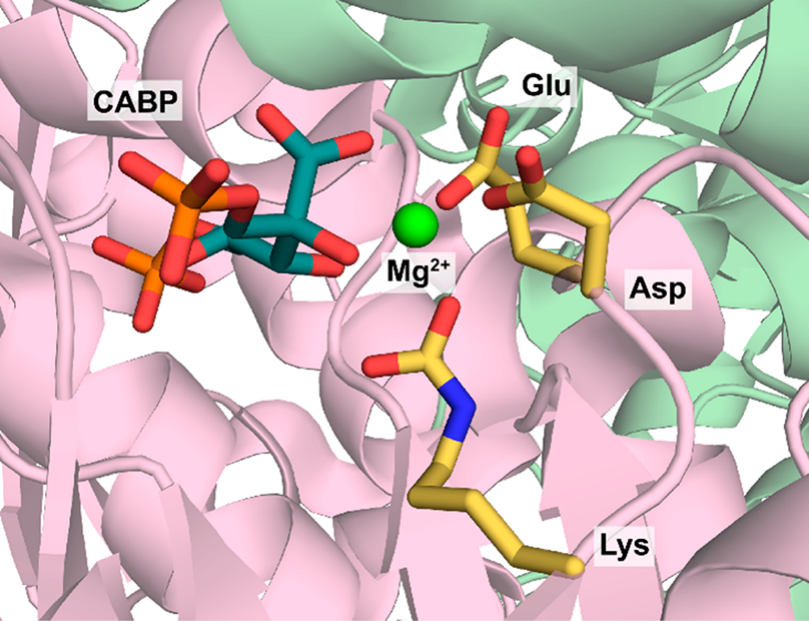
Active site of *Spinacia oleracea* RuBisCO (PDB 8RUC) lies at the dimer–dimer interface of two large subunits (pink and green) and features a Mg^2+^ ion (green). Here, the active site is occupied by the transition state analogue 2-carboxyarabinitol bisphosphate (CABP, forest green).

RuBisCO is the most important carbon fixing enzyme on the planet. Its relevance is illustrated by the fact that the total biomass of RuBisCO (roughly 0.7 Gt)^81^ exceeds the biomass of the entire human population (0.06 Gt)^82^ by 1 order of magnitude. RuBisCO is found in all domains of life, suggesting that it is an evolutionarily old protein. Four classes of RuBisCOs (forms I–IV) are known, three of which (forms I–III) catalyze the carboxylation reaction of ribulose-1,5-bisphosphate (RuBP), and the subsequent hydrolysis of the product, thereby forming two molecules of 3-phosphoglycerate (3PG, Scheme 5, Figure 1).^83−86^ Form I RuBisCOs, which are found in plants and cyanobacteria^84^ generally form hexadecameric complexes composed of eight large and eight small subunits. The small subunit is only found in form I RuBisCOs and is not essential for catalysis but contributes to specificity.^87^ The catalytically active large subunits form four dimers, with the active sites located at the dimer–dimer interface. Form II RuBisCOs are found in purple nonsulfur bacteria, dinoflagellates, and other chemoautotrophic bacteria^86^ and they are on average faster than form I RuBisCOs but tend to be less specific in distinguishing oxygen and CO_2_. Form III RuBisCO, which is found in archaea and anaerobic bacteria, has long been thought to be unable to support autotrophy. However, it has been shown that the thermophilic bacterium *Thermodesulfobium acidiphilum* can utilize its form III RuBisCO to sustain autotrophic growth, using the transaldolase variant of the CBB cycle.^88^ Both form II and form III RuBisCOs are often found as dimers but more exotic oligomers such as hexamers^89^ or decameric structures^90^ have also been reported. Apart from the three carboxylating RuBisCO forms (forms I–III), another group of RuBisCOs (form IV, also called RuBisCO-like proteins, RLPs) exists.^91^ Only a handful or RLPs have been characterized, but all of them work with substrates structurally related to RuBP and share conserved steps in their catalytic cycle. However, their biological functions are diverse, ranging from simple proton abstractions,^92^ over isomerization reactions,^93^ to decarboxylations^94^ and oxygenations.^95^

To form a catalytically competent complex, RuBisCOs require binding of a Mg^2+^ as well as carbamylation of a conserved lysine residue, which coordinates to the Mg^2+^ (Figure 1).^83,84^ The catalytic cycle of RuBisCO has been studied in detail. After coordination of RuBP to the Mg^2+^, a proton is abstracted from the C3 carbon (Scheme 5). Then, the enediolate directly reacts with CO_2_ to form an intermediate that subsequently undergoes hydrolysis, yielding two molecules of 3PG. The formal reduction of CO_2_ is achieved by concomitant oxidation of the C3 carbon of RuBP. Therefore, the overall reaction catalyzed by RuBisCO is redox neutral. Note, that formation of an enediolate is also central to the mechanism of RLPs, while the subsequent steps in their reaction mechanisms (i.e., reprotonation, oxygenation, *trans*-carboxylation) differ, giving rise to the diverse reactions, catalyzed by RLPs.

RuBisCO works under ambient conditions and does not have a dedicated CO_2_ binding site like crotonyl-CoA carboxylase/reductase (CCR, discussed below). During RuBisCO’s catalytic cycle, side reactions with other gaseous molecules, such as oxygen^44^ can occur, and indeed represent a major inefficiency in the metabolism of plants.^96^ Approximately 20% of RuBisCO’s catalytic cycles incorporate oxygen into biomass instead of CO_2_.^97^ The products of this unwanted reaction are 3PG and 2-phosphoglycolate (2PG), a toxic compound which has to be recycled in an energy intensive process that releases previously fixed CO_2_ known as photorespiration.^98^

Notably, reaction of RuBisCO’s singlet enediol intermediate with ground-state triplet O_2_ is spin-forbidden and should thus (if at all) proceed very slowly due to the high-energy barrier involved in the reaction.^99^ Oxygenation in RuBisCO as well as in other carboxylases likely proceeds via a single electron transfer (SET) mechanism, in which a single electron is transferred from the RuBP enediolate to reduce O_2_ and yield a superoxide radical, which can subsequently form a RuBP peroxide intermediate.^100^ A suited dielectric environment in RuBisCO’s active site enables this reaction sequence.^100^ The propensity of carboxylases to react with O_2_ instead of CO_2_ likely depends on a multitude of factors, including the involved metal center, the metal center’s coordination environment, outer shell protein environments, and the dielectric environment of the active site.^100^

To suppress RuBisCO’s oxygenation reaction, nature has developed several carbon concentrating mechanisms (CCMs) to increase local CO_2_ concentrations around RuBisCO to favor carboxylation over oxygenation. Prominent examples for naturally occurring CCMs include the bacterial carboxysome,^101^ the pyrenoid present in algae^102^ as well as the C4 and Crassulacean acid metabolism (CAM) in plants.^103^ Recent efforts in synthetic biology created a synthetic photorespiration pathway that converts 2PG back into the CBB cycle intermediate 3PG through tartronyl-CoA carboxylase, a new-to-nature CO_2_-fixing enzyme that compensates for the missed carboxylation reaction of RuBisCO.^55^

A less commonly discussed side reactivity of RuBisCO is reprotonation of the reactive enediolate intermediate producing xylulose 1,5-bisphosphate, 3-ketoarabinitol 1,5-bisphosphate, or 3-ketoribitol 1,5-bisphosphate.^104−106^ These compounds can act as potent RuBisCO inhibitors and nature has developed dedicated chaperones to remove these inhibitors from the active site.^107^

##### 3.1.1.2 Phosphoenolpyruvate Carboxylase (PEPC)

**Figure 2:**
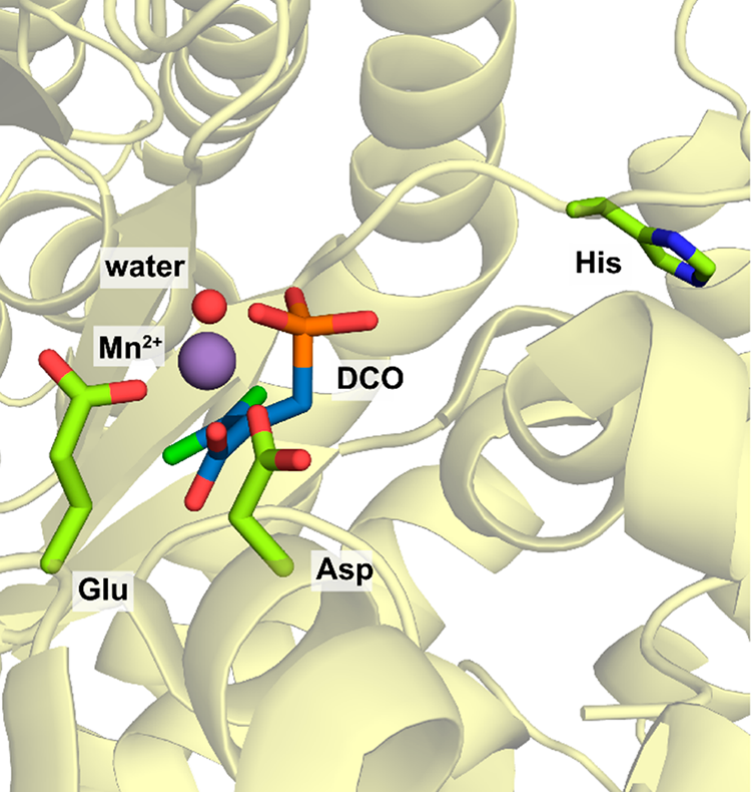
Active site of *E. coli* PEPC (PDB 1JQN) with bound Mn^2+^ (instead of naturally occurring Mg^2+^) and the substrate analogue 3,3-dichloro-2-phophonomethyl-acrylic acid (DCO) bound in the active site.

Although phosphoenolpyruvate carboxylase (PEPC) is one of the carbon-fixing enzymes in the anaerobic dicarboxylate cycle, PEPC is not oxygen sensitive and is present in many aerobic organisms, most notably in the mesophyll of C4 plants.^45^ The enzyme catalyzes the formation of oxaloacetate from PEP using bicarbonate as substrate. Like RuBisCO, PEPC features a Mg^2+^ in the active site. The Mg^2+^ is coordinated by a glutamate, an aspartate, and four water molecules (Figure 2).

**Scheme 6:**
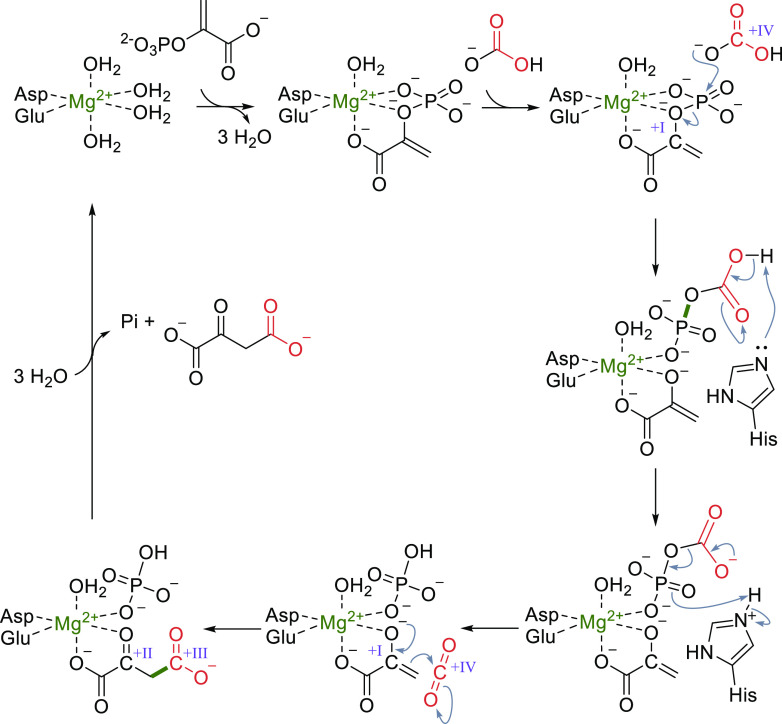
Catalytic Cycle of PEPC; Changes of the Oxidation State of Selected Carbons Are Given in Roman Numerals

Upon coordination of PEP to the Mg^2+^ as the first step in the catalytic cycle, three water molecules are released (Scheme 6). Next, bicarbonate enters the active site, triggering formation of a pyruvate enolate anion and carboxyphosphate via nucleophilic attack on the phosphate group of PEP. Carboxyphosphate is subsequently deprotonated by a conserved histidine, which is located on a flexible loop (Figure 2).^33,51,108^ Deprotonation causes elimination of the phosphate group producing CO_2_ at the active site. As a last step, the enolate anion reacts with CO_2_, thereby forming oxaloacetate. During the reaction, CO_2_ is located in a hydrophobic pocket excluding water from the active site and thereby suppressing unwanted protonation of the reactive enolate.^33^ Similarly, as in RuBisCO, the problem of reducing CO_2_ without using external reductants is solved by oxidation of the substrate (Scheme 6). However, in contrast to RuBisCO, PEPC does not suffer from the competing oxygenation reaction due to the different mechanism of carboxylation, which requires bicarbonate to undergo a series of transformations before the carboxylation step occurs. Some of the required catalytic steps, such as the nucleophilic attack on the phosphate group of PEP, are impossible with O_2_ as a substrate. As this step leads to formation of the reactive enolate while placing CO_2_ in a hydrophobic pocket in close proximity to the reactive species, side reactions with oxygen and protons (from water) are strongly suppressed. The example of PEPC nicely illustrates how specificity for CO_2_ can be achieved using enzyme mechanism as “gatekeeper”.

##### 3.1.1.3 Acetyl-CoA Carboxylase (ACC) and Acetyl-CoA/Propionyl-CoA Carboxylase (ACPCC)

**Scheme 7:**
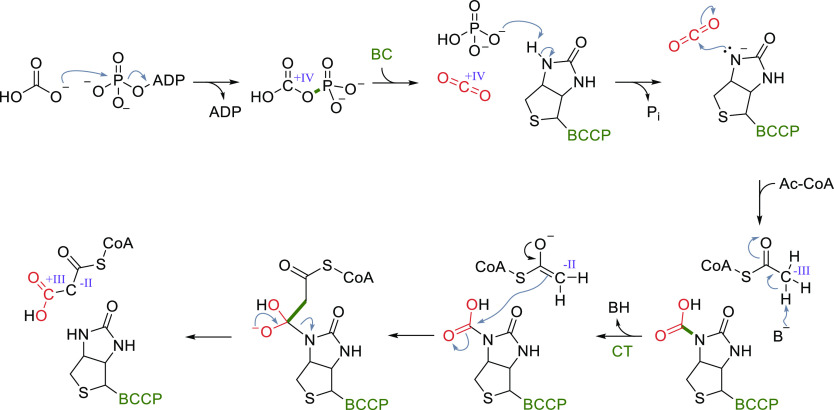
Catalytic Cycle of Acetyl-CoA/Propionyl-CoA Carboxylases Changes of the oxidation state of selected carbons are given in roman numerals. BC, biotin carboxylase; BCCP, biotin carboxyl carrier protein; CT, carboxytransferase.

Acetyl-CoA carboxylase (ACC) catalyzes the formation of malonyl-CoA, the first step in fatty acid biosynthesis and therefore ACC is found in many organisms.^109^ ACC alone, however, is insufficient for autotrophic growth on CO_2_. A characteristic feature of organisms employing either the 3HP/4HB cycle or the 3HB bicycle (section 4.1.4) is that they express an acetyl-CoA/propionyl-CoA carboxylase (ACPCC), which shows similar activity with both acetyl-CoA and propionyl-CoA.^54,110^ While ACC in eukaryotes is a single polypeptide with three domains, ACC in prokaryotes is composed of two to four subunits.^56^ The bifunctional ACPCC consists of three distinct subunits: the biotin carboxylase (BC), the carboxytransferase (CT), and the biotin carboxyl carrier protein (BCCP). In the first catalytic step, BC catalyzes formation of carboxyphosphate from bicarbonate and ATP (Scheme 7).^56^ Carboxyphosphate is proposed to spontaneously decompose into CO_2_ and phosphate. The phosphate then serves as a general base, deprotonating the biotin, which is subsequently carboxylated by reaction with CO_2_.^34^ Therefore, by investing one ATP, the enzyme gains specificity by producing a high local concentration of CO_2_ in the active site. This high local CO_2_ concentration prevents unwanted side reactions such as protonation. The carboxylation of CoA esters occurs in the CT domain. Although the exact mechanism is unknown, it likely features an acetyl-CoA enolate or a carbanion, which attacks carboxybiotin to from malonyl-CoA or methylmalonyl-CoA as product. As in RuBisCO and PEPC, reduction of CO_2_ is achieved by a formal oxidation of the substrate.

#### 3.1.2 CO_2_-Converting Enzymes with External Reductant

##### 3.1.2.1 2-Ketogluterate:Ferredoxin Oxidoreductase (2KFOR) and Pyruvate:Ferredoxin Oxidoreductase (PFOR)

The enzymes 2-ketoglutarate:ferredoxin oxidoreductase (2KFOR) and pyruvate:ferredoxin oxidoreductase (PFOR) (also referred to as 2-ketoglutarate synthase and pyruvate synthase in literature) are very similar in many aspects and both belong to the family oxoacid:ferredoxin oxidoreductases.^58^ Both 2KFOR and PFOR are thiamine pyrophosphate (TPP)-dependent enzymes that require ferredoxins as redox partners. They both use CoA esters as substrates and produce α-keto acids as products.

**Scheme 8:**
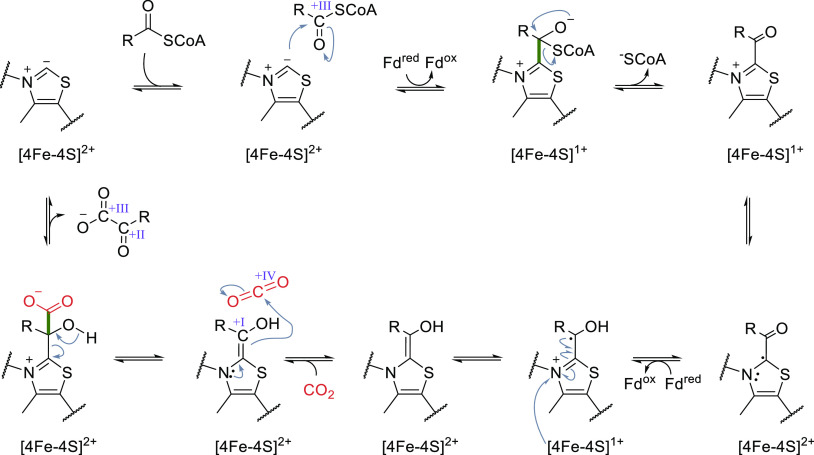
Catalytic Mechanism of Oxoacid:Ferredoxin Oxidoreductases; Oxidation States of Selected Carbons Are Highlighted in Roman Numerals

**Figure 3:**
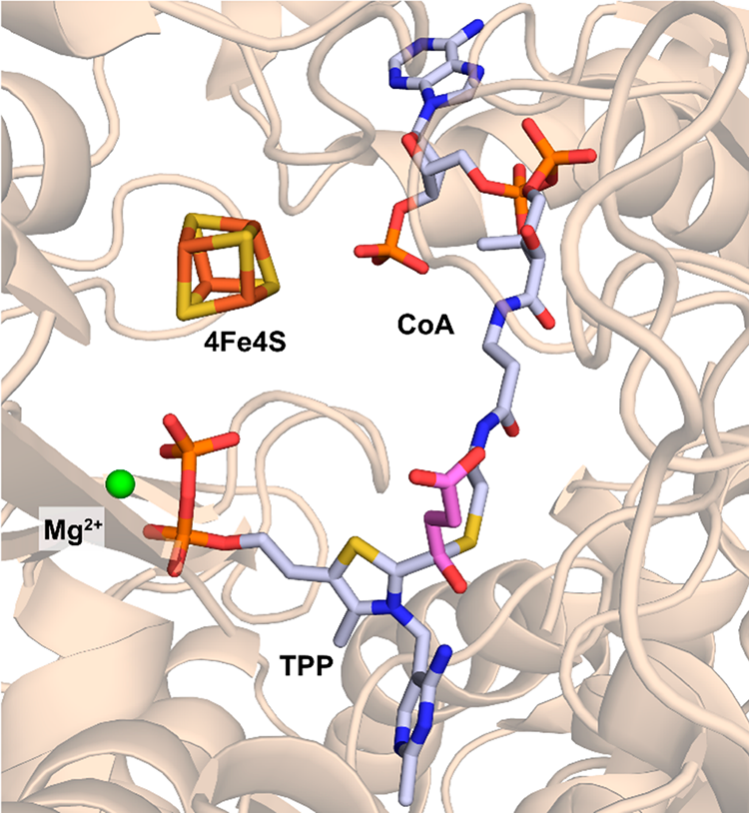
Active site of 2KFOR from *Magnetococcus marinus* with a TPP-bound succinyl-CoA intermediate present (PDB 6N2O). The carbon atoms of succinate are highlighted in pink.

The first step in the reaction mechanism (Scheme 8) is the activation of TPP by deprotonation. After reduction of the enzyme’s 4Fe-4S cluster by ferredoxin, TPP acts as nucleophile and attacks the CoA ester, forming a tetrahedral intermediate, which readily eliminates CoA. Concomitant electron transport from the 4Fe-4S cluster to the TPP intermediate results in formation of a TPP-centered radical intermediate. Reduction of the iron sulfur cluster by another ferredoxin triggers radical recombination resulting in formation of an intermediate, which can react with CO_2_. Deprotonation of the hydroxyl group leads to elimination of the product and regeneration of the resting state. The reaction mechanism of these TPP-dependent enzymes has been studied in detail, and intermediates along the reaction coordinate have been characterized spectroscopically^36−38^ and crystallographically (Figure 3).^111,112^ The reducing equivalents necessary to reduce CO_2_ are transferred from ferredoxins.

##### 3.1.2.2 Isocitrate Dehydrogenase (IDH)

Isocitrate dehydrogenase (IDH) is a canonical enzyme of the TCA cycle. While all isocitrate dehydrogenases catalyze the oxidative decarboxylation of isocitrate, only some are able to catalyze the reductive carboxylation of 2-oxoglutarate.^59,60^ As an enzyme from core metabolism, IDH is present in all organisms and often several isoforms exist in the same host. In general, two classes of IDHs occur: the NAD^+^-dependent IDHs and the NADP^+^-dependent IDHs. While IDH is a well-studied enzyme, most reports focus on the decarboxylation reaction.

**Scheme 9:**
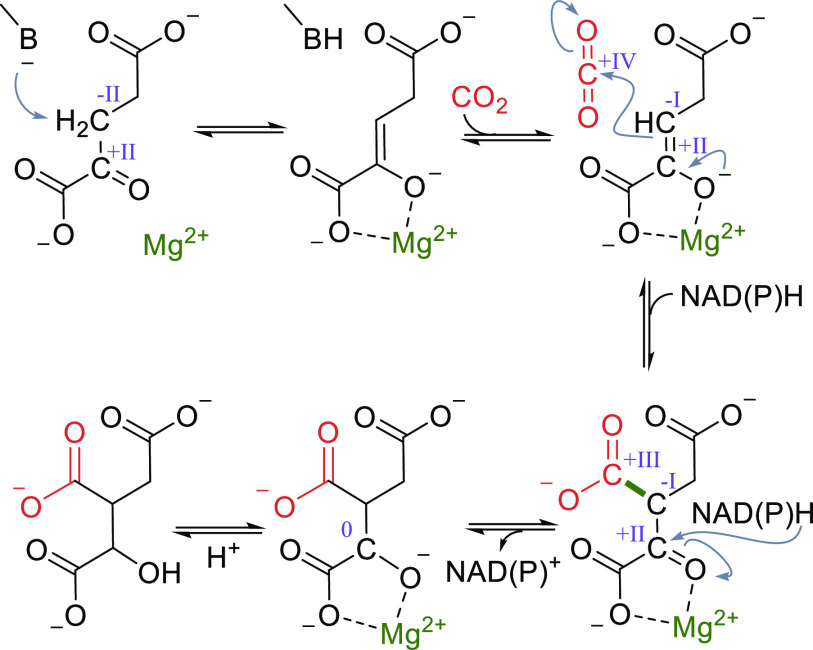
Catalytic Cycle of Isocitrate Dehydrogenase When Run in Reductive TCA Cycle; Changes of the Oxidation State of Selected Carbons Are Given in Roman Numerals

The mechanism of the reductive carboxylation catalyzed by IDH is shown in Scheme 9 and resembles to some extent the mechanism of RuBisCO. Similarly to RuBisCO, IDH contains a Mg^2+^ (or in some cases Mn^2+^) ion in the active site which coordinates the enolate of 2-oxoglutarate in a bidentate fashion. The enolized substrate subsequently reacts with CO_2_ to form the carboxylated 2-oxoacid oxalosuccinate. Finally, reduction of this intermediate by NAD(P)H yields isocitrate. Interestingly, the carboxylation step with concomitant formal reduction of the CO_2_ occurs before the NAD(P)H is consumed. The external reductant is required for the final reduction of the α-keto acid functionality.

##### 3.1.2.3 Glycine Cleavage System (GCS)

**Figure 4:**
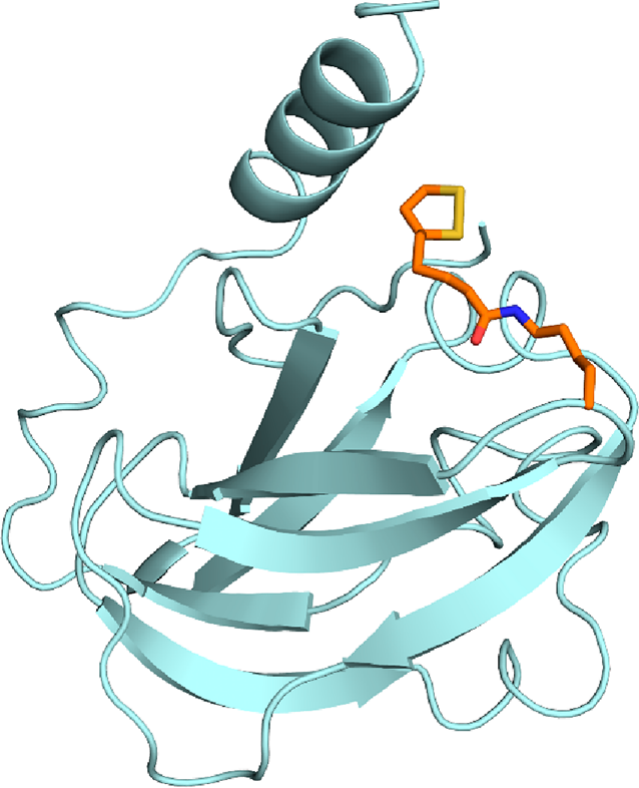
Crystal structure of the H-protein of the glycine cleavage system of *Pisum sativum* (PDB 1HPC) with the lipoic acid anchored to a lysine (highlighted in orange).

The glycine cleavage system (GCS) catalyzes the reversible transformation of glycine, tetrahydrofolate (THF), and NAD^+^ to the products 5,10-methylenetetrahydrofolate (5,10-mTHF), ammonia, NADH, and CO_2_.^24^ When acting in the forward direction, the GCS generates NADH and 5,10-mTHF that is an essential intermediate in cellular C1 metabolism (section 5). In plants, the GCS is additionally involved in the recycling of 2PG, which is a side product produced by RuBisCO.^98^ The GCS is a complex composed of four different proteins: the L-protein, the T-protein, the P-protein and the H-protein.^24^ The H-protein is responsible for shuttling intermediates to the L-, T-, and P-protein and has a lipoic acid moiety that is covalently attached to a lysine residue (Figure 4). Besides its prevalent role in glycine and serine catabolism, natural^62^ and synthetic^113−116^ examples have recently highlighted that the GCS can operate in reverse to support autotrophic growth on C1 substrates.

**Scheme 10:**
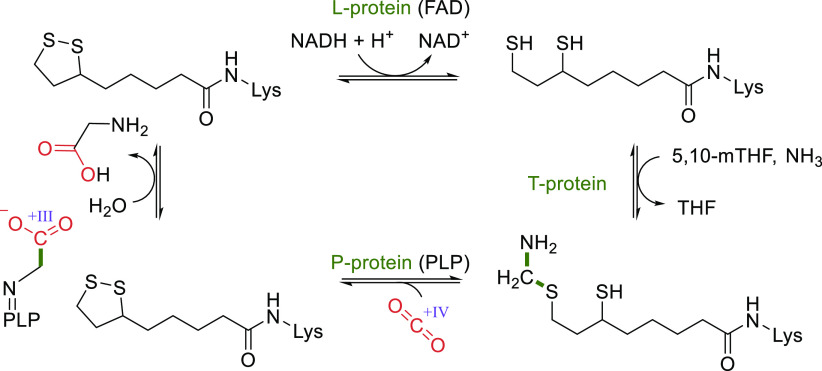
Mechanism of the Glycine Cleavage System; Changes of the Oxidation State of Selected Carbons Are Given in Roman Numerals.

During autotrophic growth, the GCS needs to operate in the reverse direction.^117−119^ The catalytic cycle in this direction starts by the L-protein-catalyzed reduction of the H-protein’s lipoic acid moiety using NADH (Scheme 10). Then, the T-protein catalyzes the reaction of the reduced lipoic acid with 5,10-mTHF and ammonia to form an aminomethyl lipoate intermediate.^24^ Finally, the pyridoxal-5′-phosphate (PLP)-dependent P-protein catalyzes the reductive carboxylation step in which CO_2_ is reduced, while the lipoic acid in the H-protein is reoxidized to form a disulfide. Recently, a detailed molecular dynamics-based study investigated the protection and T-protein-mediated release of the aminomethylation on the lipoate arm.^120^ This provided crucial insights into the mechanisms responsible for guiding the reaction along the desired reaction trajectory and showed that aminomethylation release from the lipoate arm is the rate-limiting step of the GCS reaction.^120^ Knowledge of the mechanisms and limitation of GCS can hopefully be leveraged for targeted engineering aiming to improve its carbon fixation potential.

##### 3.1.2.4 Formate Dehydrogenase (FDH) and Formylmethanofuran Dehydrogenase (FMFDH)

Formate dehydrogenase (FDH) and formylmethanofuran dehydrogenase (FMFDH) are the only known enzymes besides CO dehydrogenase (CODH, covered in the next section) that directly reduce CO_2_. While FDH catalyzes the reaction of CO_2_ to formate, FMFDH first produces formate and then directly converts it to formylmethanofuran by channeling formate through a tunnel from one active site to the next.^27^ FMFDH is found in methanogens, while FDH is found in acetogens. However, FDH is also present in other organisms, where it mainly catalyzes the oxidation of formate to CO_2_, thereby generating reducing equivalents. These can be used in formatotrophic growth using the reductive glycine pathway or the CBB cycle.

**Figure 5:**
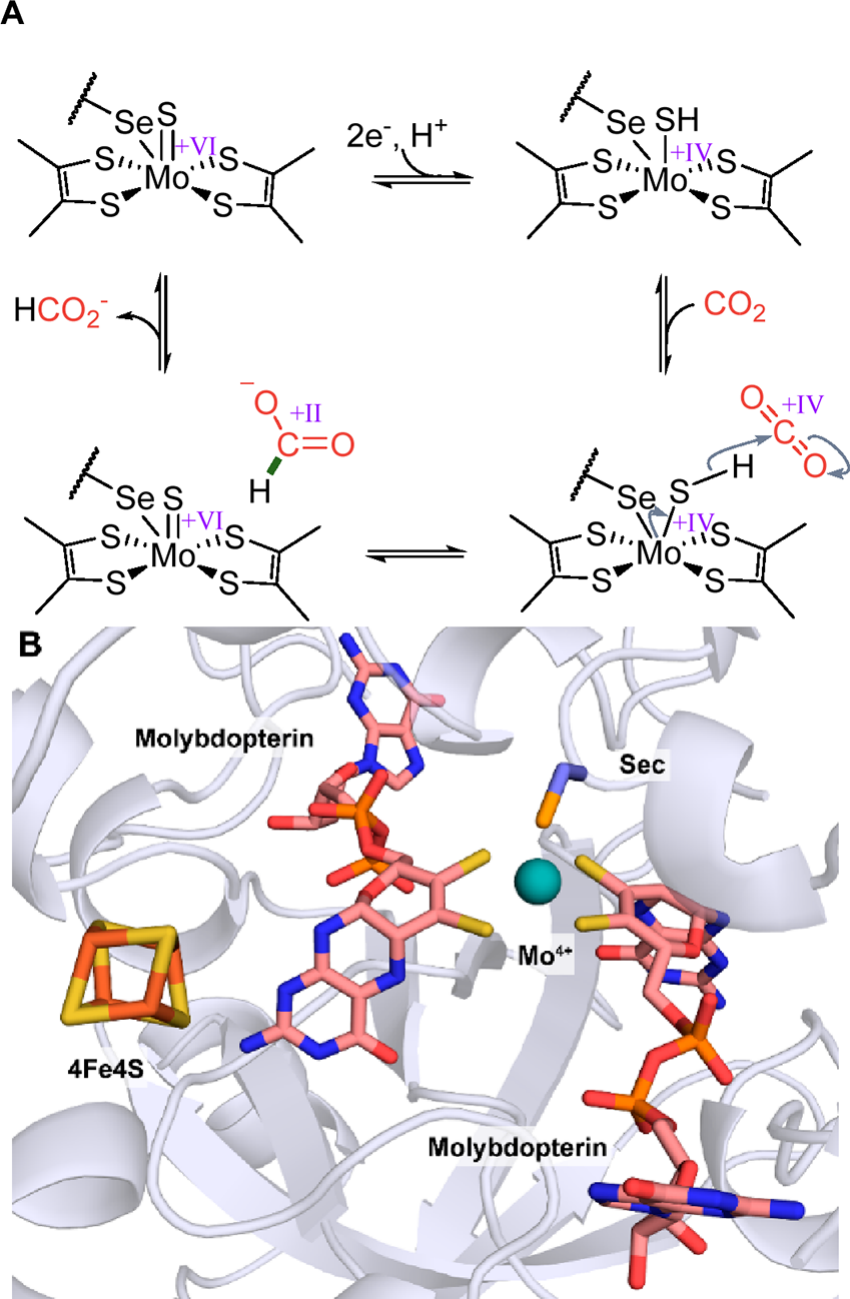
Molybdenum-dependent formate dehydrogenase (FDH). (A) Proposed catalytic cycle of molybdenum-dependent FDHs. (B) Active site of the reduced molybdenum-dependent FDH from *Escherichia coli* (PDB 1AA6).

In general, two classes of FDHs exist: NAD^+^-dependent FDHs and metal-dependent FDHs. Under physiological conditions, NAD^+^-dependent FDHs can only operate in the oxidative direction, converting formate to CO_2_. Metal-dependent FDHs and FMFDHs contain molybdenum or tungsten metal centers.^121^ In metal-dependent FDH, the central molybdenum or tungsten atom is coordinated by six ligands: four sulfur atoms from two pyranopterin ligands, a sulfur or selenium atom from a cysteine or selenocysteine, respectively, and another sulfido ligand (Figure 5).^122^ Although they usually more efficiently work in the oxidative direction, metal-dependent FDHs can reduce CO_2_. Three options exist for metal-dependent FDHs to provide sufficient reducing power: they can either use ferredoxins only, ferredoxins in combination with NADPH using an electron bifurcation mechanism,^123,124^ or they directly use molecular hydrogen (hydrogen-dependent CO_2_ reductases).^125,126^ Alternatively to using strong reducing agents, the enzymatic reaction can also be powered using electrochemical methods.^127,128^

The currently proposed reaction mechanism for the CO_2_ reduction catalyzed by the molybdenum-containing enzymes is shown representatively in Figure 5A and presumably works analogously in tungsten enzymes.^129,130^ First, the molybdenum center is reduced and the sulfido ligand is protonated forming a sulfohydryl group. Next, CO_2_ enters the active site. In a subsequent hydride transfer from the sulfohydryl group to CO_2_, formate is formed while the metal center is oxidized from Mo(IV) to Mo(VI). Although alternative mechanisms have been proposed, the mechanism shown in Figure 5A corroborates several important findings and therefore seems to be most plausible. First, it has been shown that the sulfido ligand is essential for activity,^131^ and *E. coli* has a dedicated cellular machinery composed of a cysteine desulfurase and a sulfur transferase to transfer the ligand into the active site.^65^ Second, FDH efficiently catalyzes the oxidation of formate, however the C_α_ carbon of formate is not acidic. Therefore, mechanisms involving a proton transfer seem unlikely. Lastly, EPR studies have found that during formate oxidation the C_α_ hydrogen atom of formate is transferred to the coordination shell of molybdenum, as evidenced by a strong coupling of the hydrogen with the molybdenum metal center.^64,66^ These observations are consistent with formation of a sulfohydryl intermediate.

##### 3.1.2.5 CO Dehydrogenase (CODH)

**Figure 6:**
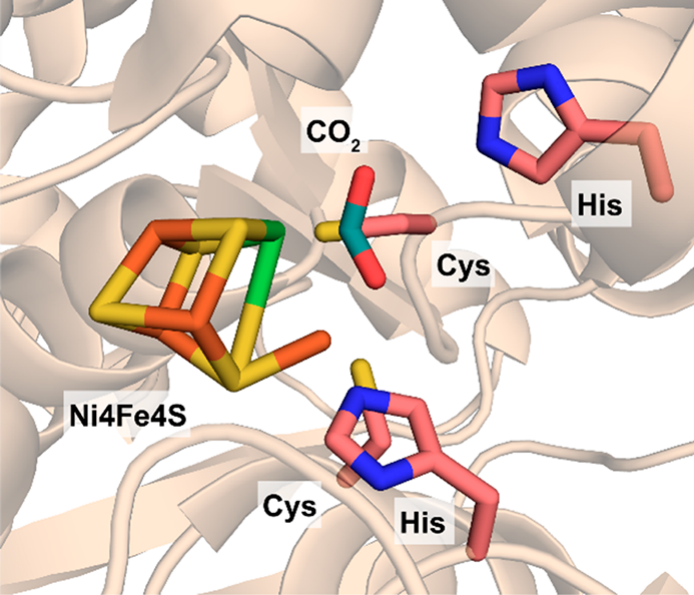
Active site of the [NiFe] CO dehydrogenase from *Carboxydothermus hydrogenoformans* with bound CO_2_ (PDB 3B52).

Two classes of CODHs are known: oxygen-sensitive nickel-iron [NiFe] CODHs and air-stable molybdenum-copper [MoCu] CODHs.^26^ While both classes can oxidize CO, only [NiFe] CODHs are able to reduce CO_2_, as the molybdenum-containing enzymes lack sufficient reducing power. The active site of the [NiFe] enzyme contains a modified 4Fe4S cluster where one iron atom is replaced with a nickel atom and an additional iron atom coordinates to a sulfur atom, forming a Ni4Fe4S complex as shown in Figure 6.^69^

**Scheme 11:**
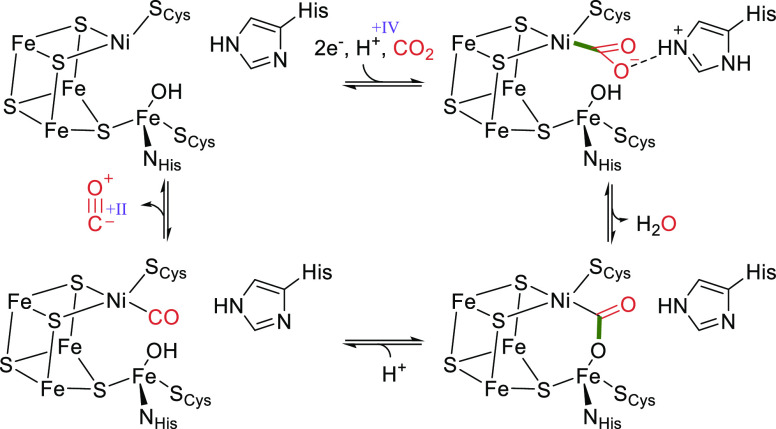
Catalytic Cycle of [NiFe] CO Dehydrogenases

The catalytic cycle in the direction of CO_2_ reduction starts with the transfer of two electrons from redox partners such as ferredoxins to the Ni4Fe4S cluster (Scheme 11). CO_2_ subsequently binds to the active site nickel atom and the anionic species is stabilized via a hydrogen bond with a protonated histidine residue which is suggested to function as general acid/base.^132^ Next, water is eliminated and an intermediate bridging the iron and nickel is formed. Cleavage of a C–O bond results in formation of an intermediate, which has been determined to be a Ni(II) species. Upon dissociation of CO, the resting state is regenerated.^26^

CODH directly reduces CO_2_ using reducing equivalents supplied by the cell (e.g., ferredoxins). A major drawback of the direct reduction of CO_2_ to CO is that CO itself is a very potent reducing agent. As a result, all CODHs catalyze the oxidation of CO more efficiently than the reverse reaction. For example, CODH from *Clostridium thermoaceticum* catalyzes CO oxidation with a *k*_cat_ of approximately 1500 s^–1^, while the reduction of CO_2_ is more than 2 orders of magnitude slower.^68^ To circumvent this problem, CO production is tightly linked to production of acetyl-CoA by coupling CODH to acetyl-CoA synthase.^133,134^

##### 3.1.2.6 Enoyl-CoA Carboxylase/Reductase (ECR)

Enoyl-CoA carboxylase/reductases (ECR) are not used in natural CO_2_ fixation pathways but rather in a pathway that enables the carbon-positive assimilation of acetate, namely the ethylmalonyl-CoA pathway.^61^ Beyond their involvement in primary metabolism, members of the ECR family can additionally act in secondary metabolism for the production of unusual extender units for polyketide synthases.^135,136^

Members of the ECR family are of interest, as they contain crotonyl-CoA carboxylase/reductase (CCR), the fastest known natural carboxylases (Table 1) and were used as the core enzymes for several non-natural CO_2_ fixation cycles^137^ (see section 4). CCRs catalyze the NADPH-dependent carboxylation of enoyl-CoA esters to produce alkyl-malonyl-CoA esters. In the absence of CO_2_, CCRs catalyze the simple reduction of enoyl-CoA esters into the corresponding saturated CoA esters as a side reaction with up to 10% catalytic efficiency.

**Scheme 12:**
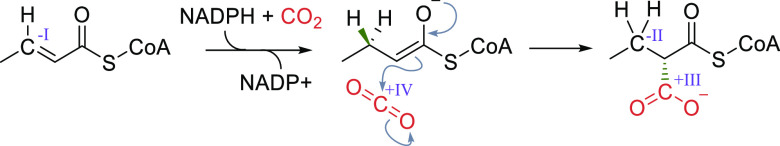
Catalytic Cycle of Crotonyl-CoA Carboxylation by Crotonyl-CoA Carboxylase/Reductase

**Figure 7:**
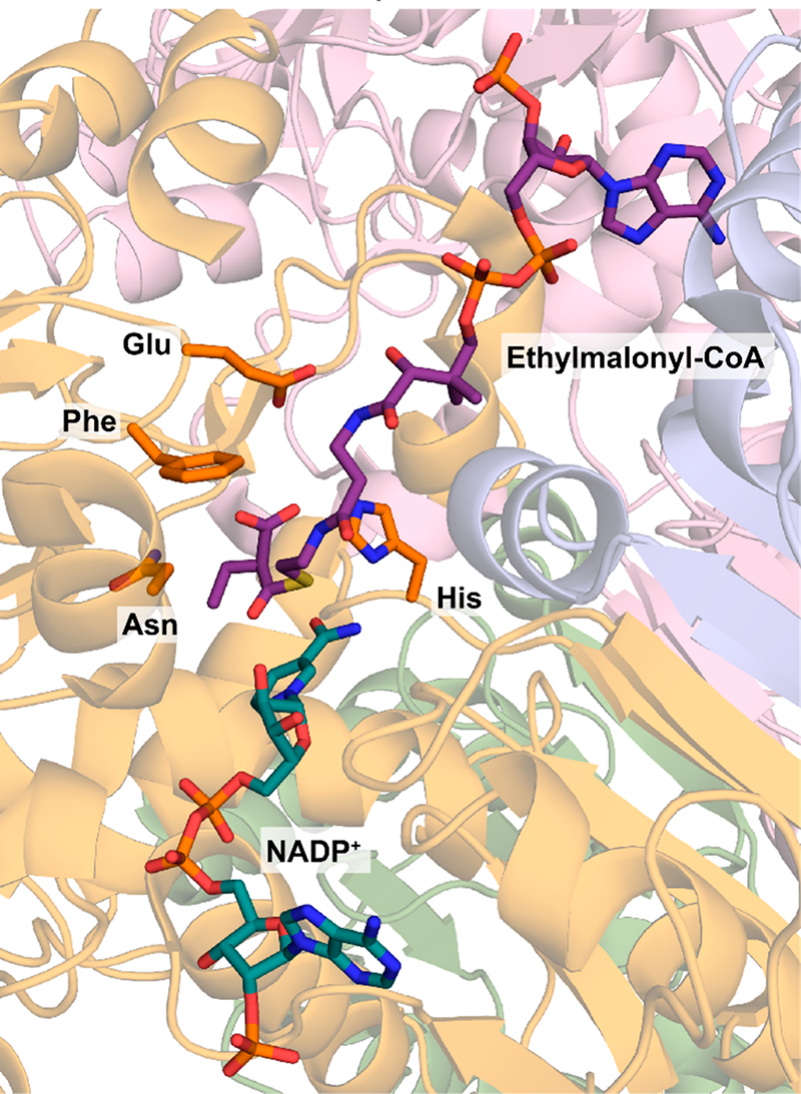
Active site of *Kitasatospora setae* crotonyl-CoA carboxylase/reductase (PDB 6OWE) with ethylmalonyl-CoA, the product of the carboxylation reaction, and NADP^+^ bound in the active site. Key active site residues necessary for accommodation of CO_2_ (His, Asn, Phe, and Glu) are highlighted in orange.

The first step in the reaction mechanism is a hydride transfer from pro-(4*R*)-NADPH to the *re*-face of the β-carbon of the enoyl-CoA ester, generating a nucleophilic enolate anion (Scheme 12).^32^ Thereafter, either CO_2_ or a proton is added, mainly in *anti*-fashion to the *re*-face at the α-carbon, to yield an alkyl-malonyl-CoA ester or a saturated CoA ester. Recent studies have shown in great detail that four amino acids: His, Asn, Glu, and Phe, form a CO_2_-binding pocket in the active site of CCR, placing the gaseous substrate in close proximity to the enolate intermediate. The hydrophobic environment provided by the phenylalanine efficiently excludes water from the active site and therefore limits unwanted irreversible protonation of the enolate intermediate (Figure 7).^138^ Furthermore, the active sites of ECRs feature elements of “negative catalysis” that guide the reaction along a defined coordinate and prevent the formation of undesired side products.^139^ In the enoyl-thioester reductase Etr1p, for example, a conserved threonine increases the energetic barrier to an alternative reaction coordinate and thus prevents the formation of an undesired covalent adduct between NADPH and the enoyl-CoA substrate.^140^ Importantly, mutation of this conserved threonine only results in a minor loss of catalytic activity while the enzyme’s error rate is drastically increased. This suggests a role of this threonine as a catalytic gatekeeper that controls the accessibility of a given reaction coordinate with little influence on the energy barrier of the desired reaction.^142^

Besides the mentioned inhibitory adduct, a different NADPH/enoyl-CoA thioester adduct has been observed when CO_2_ was omitted from the reaction or when active site residues were mutated (so-called “C2-adduct”). This C2-adduct has been used extensively to probe the ECR’s catalytic cycle, and it was shown to be accepted by ECRs, where it directly serves as an activated species in carboxylation reactions.^138,140,141^ Thus, it was speculated that the C2-adduct serves as a storage form of the high-energy enolate when no resolving CO_2_ electrophile is available. This mechanism could increase the overall reactivity of ECRs relative to other enolate-based carboxylases (such as RuBisCO), as it slows down the back-conversion of the activated enolate to the starting substrate.^138^

### 3.2 Reversing Decarboxylases for CO_2_ Fixation

Besides using natural carboxylases, another option to enzymatically incorporate CO_2_ into molecules is by operating decarboxylases in the reverse direction.^142−144^ However, due to the lack of a strong thermodynamic driving force, decarboxylases require an excess of carboxylating source (CO_2_ or bicarbonate) to shift the equilibrium toward the carboxylation side. Many of these biocatalysts operate in the secondary metabolism and/or detoxification. The robustness and broad substrate tolerance of several of these enzymes make them suitable for biocatalytic applications. Single enzyme systems have been developed for the enzymatic Kolbe–Schmitt reaction,^12,145^ among them prenylated flavin (prFMN) dependent (de)carboxylases,^146^ bivalent metal-dependent (de)carboxylases, and cofactor-independent (de)carboxylases,^10^ including a new group of enzymes based on a catalytic dyad mechanism.^147^ Furthermore, decarboxylases have been developed for the carboxylation of styrenes, polyaromatics, and heteroaromatic compounds. Recently, their synthetic applications have been reviewed by Leys et al.^10^ as well as Tomassi et al..^11^ Here we focus on mechanistic and structural aspects. In addition, TPP-dependent keto acid decarboxylases have been applied in the reversed direction to produce α-keto acids from aldehydes and CO_2_.^39,148,149^

#### 3.2.1 Bivalent Metal-Dependent (De)carboxylases

**Scheme 13:**
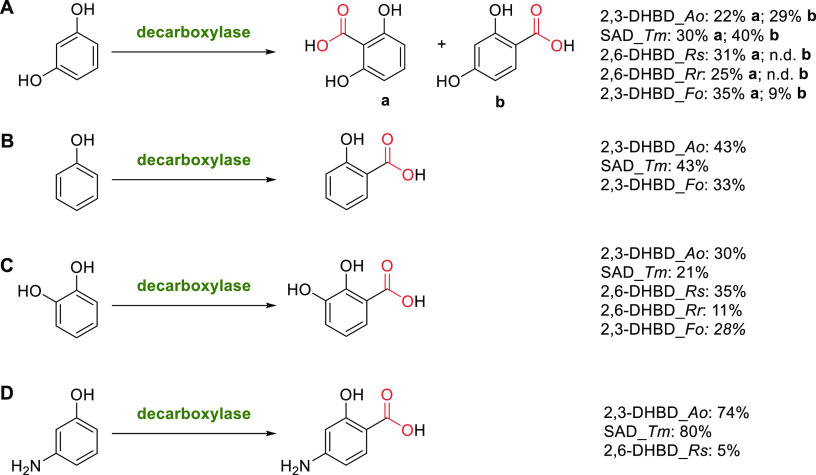
Enzymatic *ortho*-Carboxylation of (A) Resorcinol, (B) Phenol, (C) Catechol, and (D) *meta*-Aminophenol by Different Bivalent Metal-Dependent Decarboxylases^142,152,158,164,167,170^ The displayed numbers correspond to conversions to the respective product. n.d. = not determined or not found.

Due to their broad substrate scope, dihydroxybenzoic acid decarboxylases (DHAD, also termed carboxyvanillate decarboxylases,^150^ γ-resorcylic acid decarboxylases,^151^ salicylic acid decarboxylases,^152^ isoorotate decarboxylases,^153^ or orsellinic acid decarboxylases^154^) are a synthetically interesting class of catabolic decarboxylases.^11,12,14^ The enzyme family’s natural role is mostly the detoxification of phenolic acids via decarboxylation. However, native substrates have been identified in only a few cases.^150,153^ The intriguing feature of this enzyme class is that they also catalyze the carboxylation of phenols, forming phenolic acids, which represents a biochemical counterpart to the Kolbe–Schmitt reaction.^14^ In contrast to the Kolbe–Schmitt process, the enzyme class exhibits absolute selectivity for carboxylation at the *ortho*-position of phenolic substrates (and the reversed decarboxylation of the corresponding *o*-phenolic acid).^11,12^ An early example demonstrating the activity of a bivalent metal-dependent decarboxylase in the carboxylation direction was the formation of γ-resorcylic acid (2,6-dihydroxybenzoic acid) from resorcinol (1,3-dihydroxybenzene) applying 1 M KHCO_3_^–^, catalyzed by the 2,6-dihydroxybenzoic acid decarboxylase (2,6-DHBD_*Rr*) from *Rhizobium radiobacter* (cf. Scheme 13A).^142^

The enzymes belong to the amidohydrolase family and share the superfamily’s characteristic (β/α)_8_-TIM barrel fold. The catalytic activity of all characterized orthologues depends on a divalent metal ion, acting as a cofactor in the active site.^11,12^ Interestingly, the identity of this metal ion varies within the enzyme family and the individual enzymes tend to show high selectivity for their respective cofactor (Zn^2+^, Mn^2+^, and recently, Mg^2+^, have been described so far, compare Table 2).^155,156^

**Table 2:** Bivalent Metal-Dependent (De)carboxylases

enzyme	source/organism	PDB	catalytic Asp	M^2+^	substrate types	ref
2,3-dihydroxybenzoic acid decarboxylase (2,3-DHBD_*Ao*)	*Aspergillus oryzae*	7A19	Asp293	Mg^2+^	phenols, resorcinols, catechols, naphthols, aminophenols, styrenes, coumaric acids and esters, phloretic acids, polyphenols	(29,152,155,157−163)
2,3-dihydroxybenzoic acid decarboxylase (2,3-DHBD_*Fo*)	*Fusarium oxysporum*	7BP1 (substrate bound)	Asp291	Zn^2+^	phenols, resorcinols, catechols	(164,165)
2,6-dihydroxybenzoic acid decarboxylase (2,6-DHBD_*Rs*)^c^	*Rhizobium* sp. (MTP-10005)	2DVU (substrate bound)	Asp287	Zn^2+^	phenols, resorcinols, catechols, naphthols, aminophenols, styrenes, coumaric esters, polyphenols	(48,144,151,152,158,160,166)
2,6-dihydroxybenzoic acid decarboxylase (2,6-DHBD_*Ps*)	*Polaromonas* sp. (JS666)	4QRO (inhibitor bound)	Asp287	Mn^2+^	decarboxylation of γ-resorcylate and derivatives^b^	(156)
2,6-dihydroxybenzoic acid decarboxylase (2,6-DHBD_*Rr*)^c^	*Rhizobium radiobacter* WU-0108		Asp287^a^	nd	phenols, resorcinols, catechols, polyphenols	(142,167)
salicylic acid decarboxylase (SAD_*Tm*)	*Trichosporon moniliiforme*	6JQW	Asp298	Zn^2+^	phenols, resorcinols, catechols, naphthols, aminophenols, styrenes, coumaric esters, polyphenols	(152,158,160,168−170)
5-carboxyvanillate decarboxylase (LigW_*Sp*)	*Sphingomonas paucimobilis* SYK-6	4ICM (substrate bound)	Asp296	Mn^2+^	phenols, guajacols, coumaric acids, phloretic acids	(150,162,171,172)
5-carboxyvanillate decarboxylase 2 (LigW2_*Sp*)	*Sphingomonas paucimobilis* SYK-6		Asp313^a^	nd	phenols, guajacols, coumaric acids, phloretic acids	(150,162)
5-carboxyvanillate decarboxylase (LigW_*Na*)	*Novosphingobium aromaticivorans*	4QRN (substrate bound)	Asp314	Mn^2+^	decarboxylation of 5-carboxyvanillate^b^	(172)
iso-orotate decarboxylase (IDC_*Cm*)	*Cordyceps militaris* CM01	4HK5 (apo form)	Asp323	Mn^2+^	decarboxylation of 5-carboxy-uracil^b^	(153)

a Based on sequence alignment.

b Reaction in decarboxylation direction.

c Also: γ-resorcylate decarboxylase; nd = not determined.

**Figure 8:**
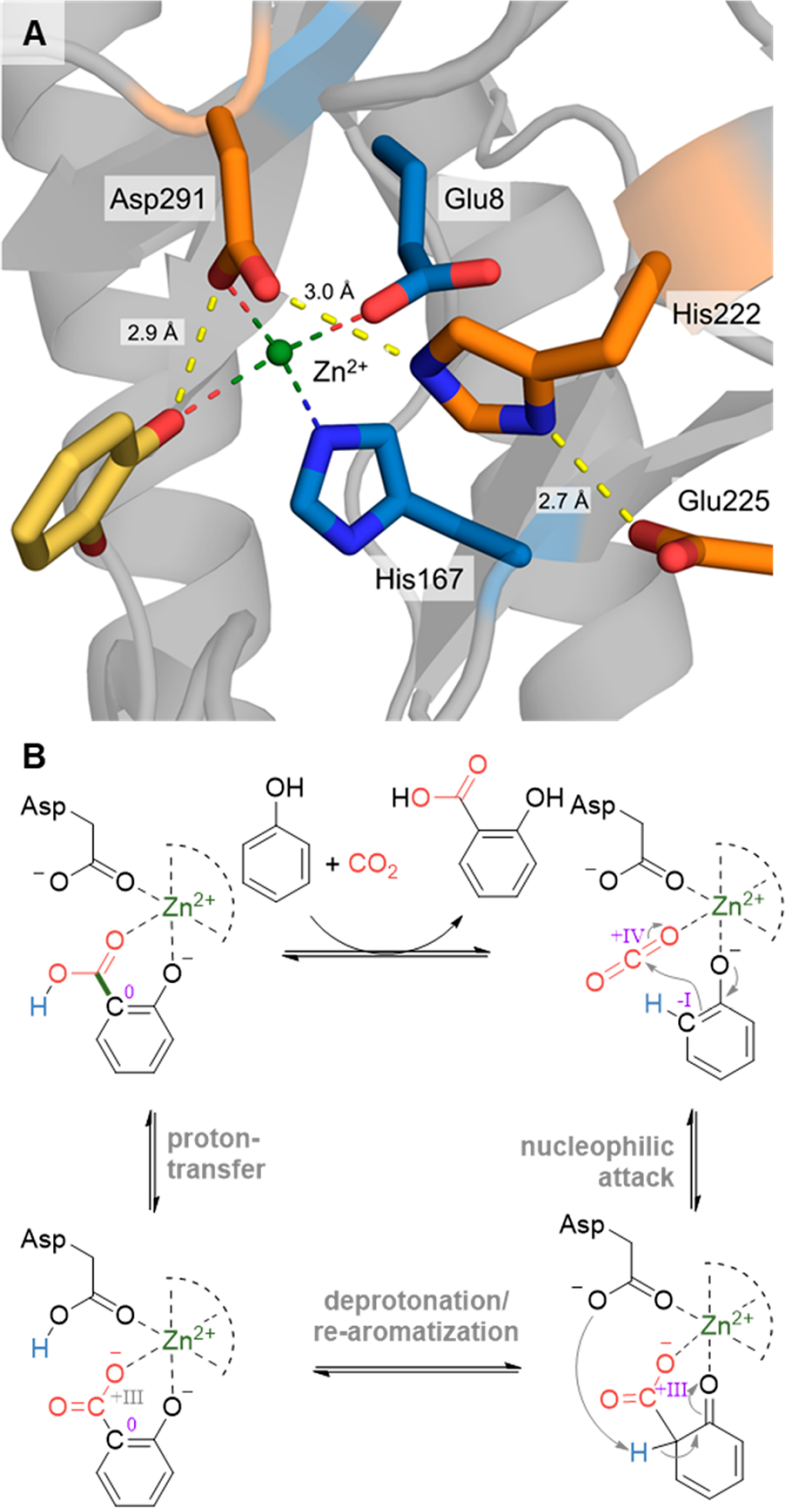
(A) View of the active site of the 2,3-DHBD_Fo from *Fusarium oxysporum*, bound substrate catechol (yellow). The catalytic Zn^2+^ is displayed as a green ball, and the complexing amino acids (Glu8, His167, and Asp291) and the catalytic triad (Asp291, His222, and Glu225) are displayed as sticks (PDP 7BP1).^165^ (B) General mechanism of the (de)carboxylation of phenol by a bivalent metal-dependent decarboxylase (Zn^2+^ is shown as example and can be Mg^2+^ or Mn^2+^ in other enzymes).

Structural and mutational studies have identified a range of amino acids crucial for activity (Figure 8A). In all crystal structures, the metal cofactor is complexed by either three or four amino acids, as for example Glu8, His167, Asp293, and three water molecules in the case of the 2,3-DHBD_*Ao* from *Aspergillus oryzae*.^155^ Mutation of these residues usually results in insoluble protein or diminished activity.^165,170,172^ Tight binding of the phenolic substrates is facilitated by hydrophobic interactions with amino acids such as phenylalanine or proline (e.g., Phe27, Pro189, Phe193, and Phe294 in the case of 2,3-DHBD_*Fo* from *Fusarium oxysporum*),^165^ and coordination of the hydroxy-group at the catalytic metal center. The residues, that perform the mechanistically crucial proton transfer (vide infra), are often arranged in a triad, consisting of asparagine, glutamine, and histidine (e.g., Asp293, His222, and Glu225 for 2,3-DHBD_*Ao*^29^ or Asp291, His222, and Glu225 for DHBD_*Fo*^165^). Mutation of these residues, for example in LigW_*Sp* (Asp296, His226, Glu229) lowered the enzyme’s *k*_cat_ by several orders of magnitude.^172^

While the mechanism of the decarboxylation reaction is well studied experimentally and by computational methods, the carboxylation reaction has only recently been investigated using quantum chemical calculations.^29,30,155,172^ In general, the steps resemble the electrophilic aromatic substitution mechanism of the Kolbe–Schmitt reaction: both CO_2_ and the deprotonated phenolic substrate bind to the bivalent metal in the active site (Figure 8B). The C–C bond is formed in a nucleophilic attack of the *ortho*-carbon onto CO_2_, resulting in a dearomatized intermediary cyclic dienone. Deprotonation of the *ortho*-position is performed by the catalytically-active Asp (Table 2) and accelerated by a His and Glu residue, which form a catalytic triad. This results in rearomatization of the intermediate, forming the deprotonated phenolic acid product bound as a chelate to the bivalent metal. Although in theory, the mechanism could feature first proton transfer followed by carboxylation, density functional theory (DFT) calculations (for both carboxylation and decarboxylation),^29,156,173,174^ and isotope labeling studies (for decarboxylation)^172^ suggest the displayed order of events.

The identity of the CO_2_-source that initially binds in the active site is still a matter of debate. While reactions under CO_2_-pressure point to bicarbonate (HCO_3_^–^),^48^ recent quantum chemical investigations, paired with exact determinations of the CO_2_ partial pressure in solution, came to the conclusion that CO_2_ is the molecule that is utilized by the enzyme.^29^ Furthermore, mass spectrometry provided evidence that the initial product of the decarboxylation reaction is CO_2_, rather than HCO_3_^–^.^174^ However, the source of the carboxylate might be different for different enzymes.

Due to the uncomplicated setup, the majority of biocatalytic carboxylation reactions using this enzyme class are performed using bicarbonate salts (mostly the potassium salt but also others were evaluated^166^) as a CO_2_ source.^152,162,164,166^ High concentrations between 1 and 3 M are usually applied to drive the equilibrium. Note that such high bicarbonate concentrations either require exceptionally concentrated buffers, or are applied as unbuffered solution at its pH of 8.3.^48^ Alternatively, the reaction is run by direct supply of CO_2_, either via bubbling^175^ or at high pressure under CO_2_ atmosphere.^29,48,152,159,163^ Note the addition of ascorbic acid allows suppression of spontaneous oxidation of resorcinol- and catechol-derivatives and therefore to run the reaction under air.^48^

There is a small number of “classic” carboxylation substrates (Scheme 13) that most members of the enzyme family were characterized with, namely resorcinol (A), phenol (B), catechol (C), and *meta*-aminophenol (D).^142,152,158,164,167,170^ The second hydroxy group of the dihydroxybenzenes serves as additional handle, allowing better binding of the substrate in the active site. Due to its application as antituberculous agent, *para*-aminosalicylic acid (D), was identified early as a promising target compound.^159,169,170^

The enzymes also carboxylate bulkier compounds such as naphthols, styrenes, *p*-coumaric acids, esters, and polyphenols.^11,12^ The minimal structural requirements for a substrate is a phenolic hydroxy group with an accessible *ortho*-position.^12^Table 2 gives a brief overview of generic substrate types that the individual enzymes have been characterized with.

Carboxylations catalyzed by metal dependent decarboxylases proceeded with absolute regioselectivity, with the exception of resorcinol, which is carboxylated at the two *ortho*-positions in roughly equal amounts (22% and 29%, respectively, Scheme 13A).^158^

Although the enzymes are highly regiospecific, they accept a range of bulky substituents at the phenolic *ortho*- and *meta*-positions. A number of examples, including coumaric acid and ester, phloretic acid, the phenyl pyruvic ester, and even polyphenols such as resveratrol.^160,162,167^

#### 3.2.2 Cofactor-Independent (De)carboxylases

Phenolic acid decarboxylases (PADs) represent a further class of enzymes from secondary metabolism which have been successfully applied in biocatalytic carboxylation reactions.^12,152,176^ It is noteworthy that PADs do not require any cofactor or metal ion for catalysis. In nature, they are involved in the biodegradation of cinnamic acid derivatives such as ferulic, coumaric, and caffeic acids, the latter derived from the oxidative breakdown of lignin.^177,178^ Several enzymes from bacterial sources which share a sequence identity within a range of ∼40–80% have been shown to be able to carboxylate *para*-hydroxystyrene derivatives regioselectively at the β-atom of the side chain to yield the corresponding cinnamic acid derivatives exclusively in (*E*)-configuration (Table 3).^176^ In order to enable PADs to run the reaction in the reverse carboxylation direction, elevated concentrations of the CO_2_ source such as bicarbonate are required.^152,176^

**Table 3:** Overview of Cofactor-Independent Decarboxylases

enzyme	source/organism	PDB	catalytic Glu	ref
phenolic acid decarboxylase (PAD_*Lp*)	*Lactobacillus plantarium*	2W2A	Glu71	(176,179,180)
phenolic acid decarboxylase (PAD_*Ba*)	*Bacillus amylodiquefaciens*			(176,181)
phenolic acid decarboxylase (PAD_*Bl*)	*Bacillus licheniformis*			(176)
phenolic acid decarboxylase (PAD_*Bs*)	*Bacillus subtilis*	2P8G (4ALB, Tyr19Ala)	Glu64	(176,182,183)
phenolic acid decarboxylase (PAD_*Mc*)	*Mycobacterium colombiense*			(176)
phenolic acid decarboxylase (PAD_*Ms*)	*Methylobacterium* sp.			(176)
phenolic acid decarboxylase (PAD_*Ps*)	*Panteo* sp.			(176)
ferulic acid decarboxylase (FDC_*Es*)	*Enterobacter* sp.	3NX1, 3NX2, 4UU3	Glu72	(176,184)

**Figure 9:**
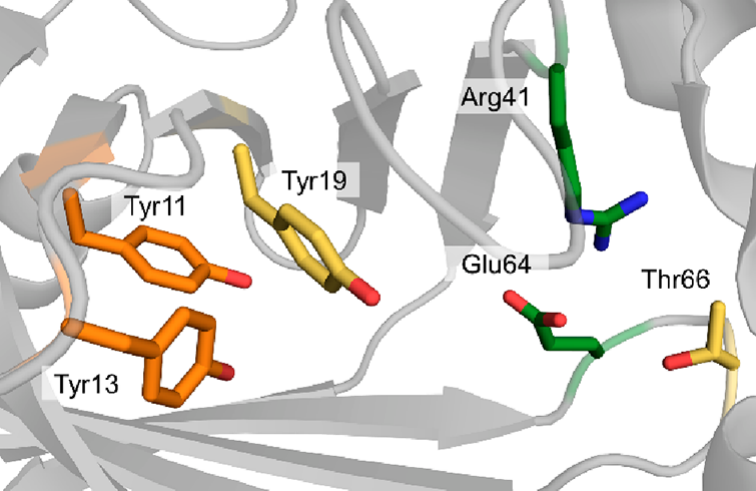
Active site of the phenolic acid decarboxylase from *Bacillus subtilis*. Amino acid residues involved in the hydrogen-bonding network of the substrate (Tyr11, Tyr13) and the carboxylating source (Tyr19, Tyr66) as well as the glutamate residue acting as the catalytically important general acid are displayed as sticks (PDP 2P8G).

**Scheme 14:**
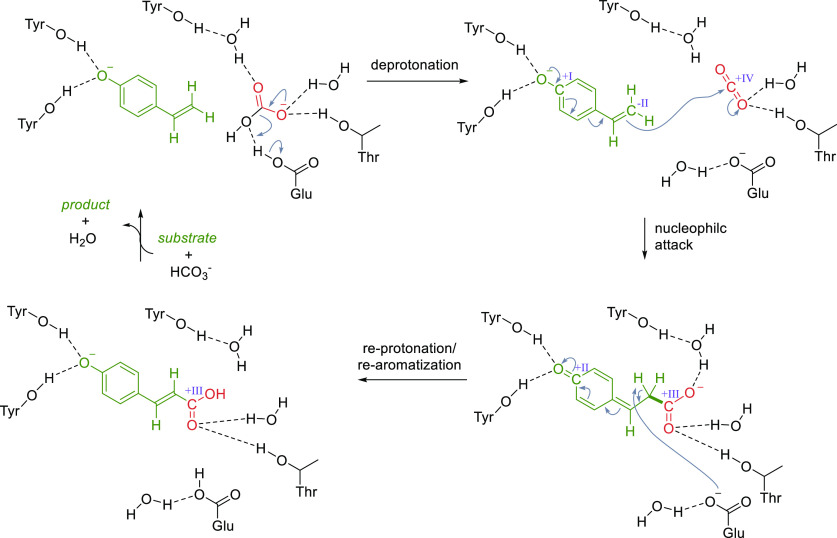
Catalytic Cycle of the Carboxylation, Catalyzed by Phenolic Acid Decarboxylases, Shown for *para*-Vinylphenol by a Cofactor-Independent Phenolic Acid Decarboxylase^31,185^

In particular, the structure of the phenolic acid decarboxylase from *Bacillus subtilis* (Figure 9),^182^ combined with quantum chemical calculations using a large active site model (>300 atoms), has facilitated a detailed proposal of the reaction mechanism which proceeds via classical acid–base catalysis (Scheme 14).^31,185^ A highly conserved glutamate residue (e.g., Glu64 in PAD_*Bs*) acts as general acid, transferring a proton to bicarbonate to generate carbon dioxide as the actual carboxylating agent. Note that the reaction with CO_2_ was calculated to be energetically much more feasible than the reaction with bicarbonate.^31^ The β-carbon atom of the styrene side chain then performs a nucleophilic attack on carbon dioxide to yield a quinone methide intermediate. This step is supported by two tyrosine residues (e.g., Tyr11, Tyr13 in PAD_*Bs*), which interact with the *para*-hydroxy group via hydrogen bonding. Reprotonation of the glutamate residue, which goes in hand with the rearomatization, yields the final (*E*)-cinnamic acid derivatives.

**Figure 10:**
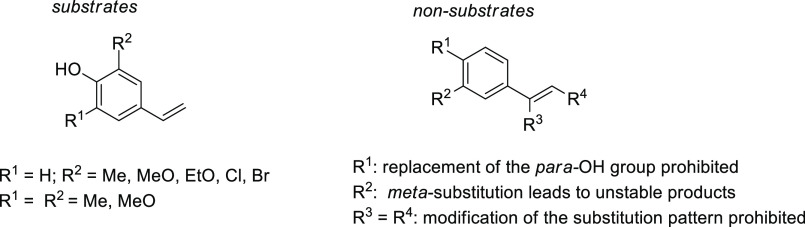
General substrate scope of the side chain carboxylation of styrene derivatives by phenolic acid decarboxylases.^176^

Biocatalytic characterization studies (such as substrate scope, reaction conditions, etc.)^166,176^ were performed to evaluate the potential of phenolic acid decarboxylases as carboxylation tool. Compared to the regio-complementary *ortho*- and *para*-selective decarboxylases (see sections 3.2.1 and 3.2.3), PADs display a more limited substrate tolerance as well as a narrow operational window concerning the reaction parameters (substrate concentration, pH-, and temperature range). Their independence of a cofactor as well as the lack of alternative chemical methods for the side chain carboxylation of styrenes (except a Pd-catalyzed method for substituted 2-hydroxystryrens)^186^ are strong arguments for their consideration as biocatalytic carboxylation tool. However, potential styrene-type substrates need to fulfill various features in order to be accepted by enzyme candidates that have been characterized so far: A fully conjugated system along the substrate and a *para*-hydroxy group are both mandatory to facilitate the required resonance stabilization of the negative charge via the quinone methide intermediate (Figure 10).

Substituents in the position *ortho* to the hydroxy group are well tolerated independent of their electronic nature. Therefore, conversions up to 35%, in the case of the *ortho*-methoxy monosubstituted hydroxystyrene, were achieved.^176^ Modification of the substitution pattern in the α- or β-position of the styrene side chain or replacing the catalytically relevant *para*-OH group (e.g., by H, Cl, OMe, NH_2_), is not accepted and leads to a loss of enzymatic activity.

#### 3.2.3 prFMN-Dependent (De)carboxylases

**Scheme 15:**
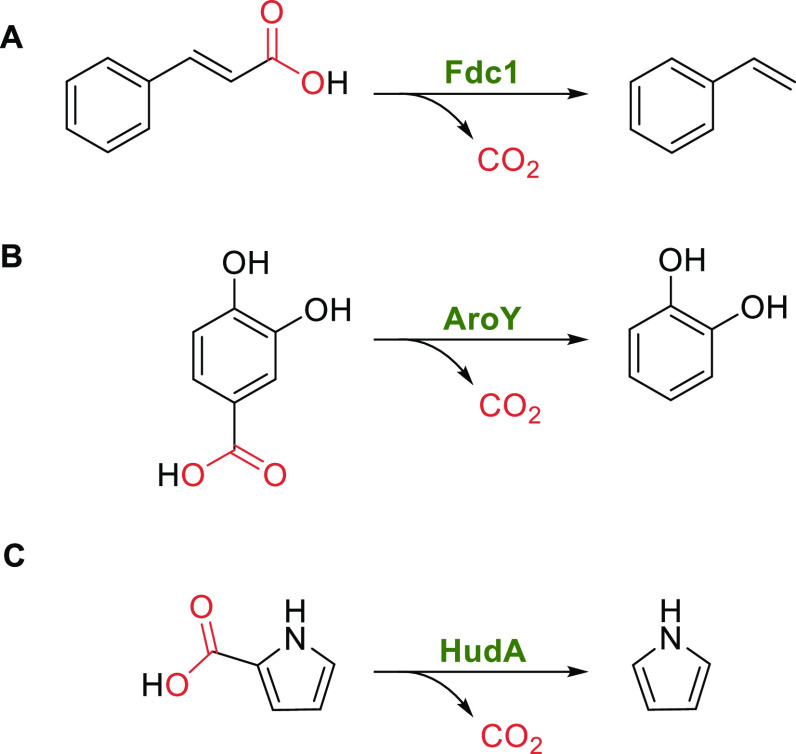
Overview on prFMN-Dependent Decarboxylation Reactions: (A) Decarboxylation of Cinnamic Acids by Ferulic Acid Decarboxylases; (B) Decarboxylation of Phenolic Substrates by AroY Type Enzymes; (C) Decarboxylation of Heteroaromatic Substrates by Pyrrole-2-carboxylate Decarboxylase from *Pseudomonas aeruginosa*

In the recent decades, a number of decarboxylases with intriguing reactivities have been characterized, catalyzing e.g., *para*-decarboxylation of phenolic carboxylic acids,^187^ decarboxylations of polyaromatic hydrocarbons (PAHs),^188^ and heteroaromatics.^189,190^ However, it took further research until these reactions and the underlying mechanisms were fully understood. After the discovery of prenylated FMN (prFMN) by Leys et al.^191^ in 2015, the cofactor was confirmed to be present in several other members of the UbiX-UbiD family and it was shown to be associated with decarboxylase function.^35,146,192−194^ The substrate scope of different subfamilies of prFMN-dependent decarboxylases is quite diverse (Scheme 15) and encompasses cinnamic acids (forming styrenes), phenolic carboxylic acids (forming phenols), and heteroaromatic carboxylic acids, (forming heteroaromatic molecules). Furthermore, these species are (de)carboxylated using distinct mechanisms, all involving covalent binding of the substrate to the cofactor. In order to provide a comparative overview of the action of these subclasses, this section is divided into further subsections, discussing the different enzymes side-by-side.

##### 3.2.3.1 Prenylated FMN (prFMN) Biosynthesis and Maturation

**Scheme 16:**
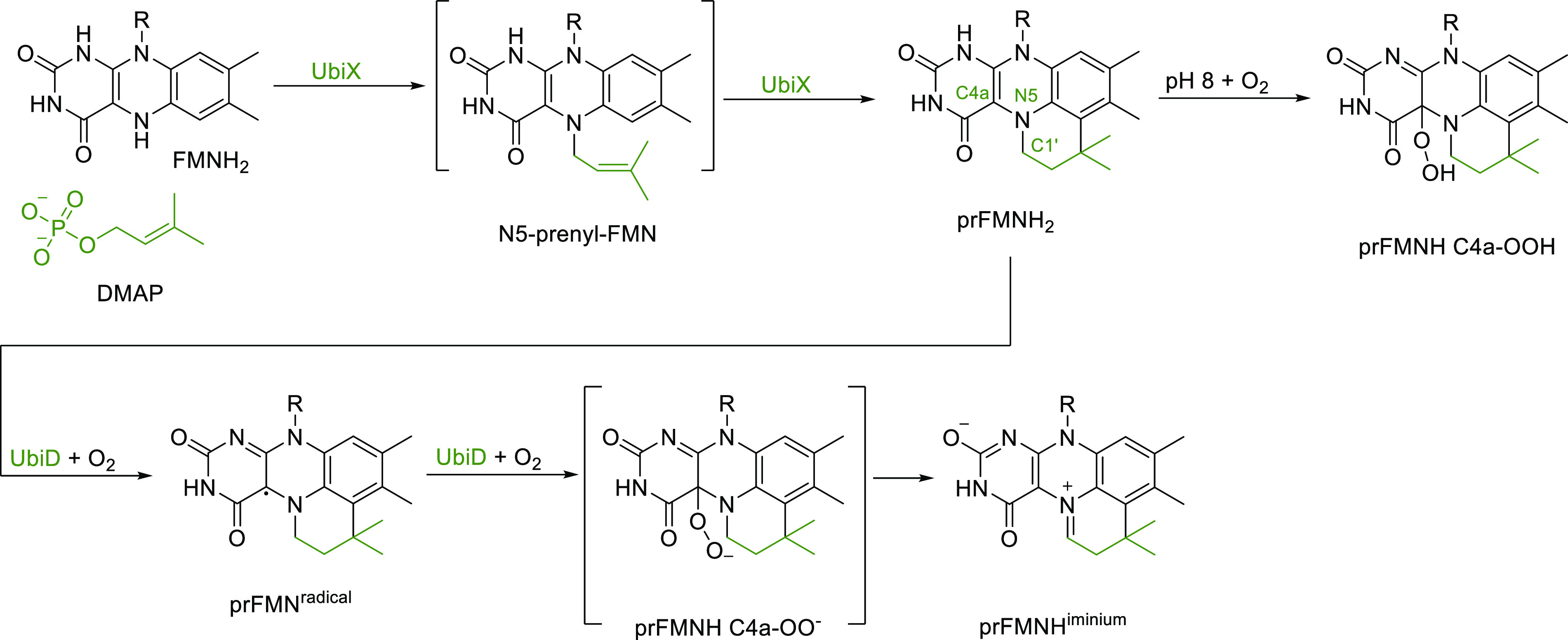
Biosynthesis of the prFMN Cofactor (prFMNH_2_) by the UbiX Enzyme Oxidative maturation of the bound cofactor is required to reach the catalytically active iminium form (prFMNH^iminium^). Free prFMNH_2_ in the presence of oxygen degrades to prFMN-hydroperoxide (prFMNH C4a-OOH).

In nature, the enzymes UbiX and UbiD are involved in the biosynthesis of ubiquinone, a cofactor responsible for electron transport in proteobacteria and eukaryotes.^195^ UbiX is a flavin prenyltransferase responsible for the prenylation of FMN by connecting a diemethylallyl moiety to FMN. This creates a fourth nonaromatic ring via a mechanism resembling class I terpene cyclases (Scheme 16).^195−199^ UbiD and its homologues in turn bind the prFMN cofactor in their active site, enabling reactions such as the decarboxylation of ferulic acid catalyzed by the UbiD homologue ferulic acid decarboxylase (Fdc).^200^ Note that PAD1 and Fdc are isofunctional to UbiX/UbiD.^200^

In detail, UbiX is a dodecameric metal-independent enzyme, with monomers that represent a typical Rossmann fold. FMN and two additional sulfate ions bind at the interface of two subunits.^197^ UbiX utilizes either dimethylallyl monophosphate (DMAP) or dimethylallyl pyrophosphate (DMAPP) for the prenylation of N5, which is followed by an intramolecular Friedel–Crafts type alkylation of the flavin’s C6.^198^ Due to blocking of the cofactor’s N5 position, the usual flavin chemistry as well as photocatalysis are not possible with prFMN.^35,192^

The prFMNH_2_ biosynthesized by UbiX binds to Fdc1, a fungal UbiD homologue.^201^ In the enzyme, the cofactor is coordinated by the metal ions Mn^2+^ and K^+^ with its phosphate moiety.^187,191^ After binding, the cofactor requires oxidative maturation via a radical and a peroxo-species to produce the catalytically active iminium species (Scheme 16).^194,198,199^ Oxidation of unbound prFMNH_2_ results in formation of prFMN-hydroperoxide, a dead-end species that does not assist in enzyme activity.^202^ The catalytically active prFMNH^iminium^ cofactor is highly sensitive. For example, exposure to light causes tautomerization to the ketimine form inducing irreversible enzyme inactivation.^203^

In Fdc, Arg173 (*A. niger* nomenclature) is a key residue for cofactor maturation because mutation to alanine or lysine results in accumulation of a prFMN^radical^ species. Nevertheless, Arg173Ala displays low levels of activity, indicating that cofactor maturation still occurs, albeit significantly slower.^203^ As oxygen is required for prFMNH^iminium^ formation, its maturation mechanism for homologues active in anaerobic hosts needs to proceed via an alternative pathway that is independent of O_2_. Fe^2+^ is suspected to play a pivotal role in anaerobic oxidative prFMN maturation.^203−206^

Production of active UbiD requires either heterologous coexpression of UbiX and UbiD or reconstitution of apo-UbiD with prFMNH_2_ and its subsequent activation. Several protocols for in vitro and in vivo FMN prenylation and maturation have been described.^207−209^

##### 3.2.3.2 Summary of Known prFMN-Dependent Decarboxylases with Confirmed Carboxylation Activity

In order to understand the evolutionary history of the prenylated cofactor, prFMN-dependent decarboxylases have been subjected to phylogenetic analysis.^10,12,35,146,192^ Shen et al. reported a phylogenetic tree containing more than 200 homologues of TtnD, a prFMN dependent decarboxylase acting in the tautomycetin biosynthetic pathway. The analysis revealed three main clusters that fit to the enzyme’s substrate specificities: decarboxylases acting on (i) aromatic carboxylic acids, (ii) cinnamic acids, and (iii) aliphatic α,β-unsaturated acids.^210^ The number of putative prFMN-dependent decarboxylases without confirmed prFMN cofactor is still immense. As this review focuses on CO_2_ fixation, the following discussion will highlight enzymes, which not only perform decarboxylation reactions but also catalyze carboxylation reactions.

Table 4 presents a selection of characterized prFMN dependent decarboxylases that were tested for the reversible decarboxylation, along with their preferred substrate classes.

**Table 4:** Selected prFMN Dependent Decarboxylases That Were Applied for Carboxylation

enzyme	source organism	PDB	substrate type	ref
ferulic acid decarboxylase (*An*Fdc)	*Aspergillus niger*	4ZA4 (prFMN in iminium form), 4ZA7 (with α-methyl cinnamic acid bound)	styrenes	(191,211−213)
phenolphosphate carboxylase	*Thauera aromatica*		phenolic compounds	(18,80)
*Sc*Fdc	*Saccharomyces cerevisiae*	4S13, 4ZAC (prFMN in iminium form)	aryl and heteroaryl styrenes	(212,214−217)
HmfF	*Pelotomaculum thermopropionicum*	6H6X (with prFMN), 6H6V (with FMN)	heteroaromatic substrates	(218)
*Kp*AroY AroY (3,4-dihydroxybenzoic acid/protocatechuic acid decarboxylase)	*Klebsiella pneumoniae* (*Aerobacter aerogenes*)	5O3M	catechols	(187,219)
*Ec*AroY	*Enterobacter cloacae*	5O3N, 5NY5	catechols	(187,220)
VdcCD (vanillate and 4-hydroxybenzoate decarboxylases)	*Streptomyces* sp. D7		catechols	(219)
PYR2910	*Bacillus megaterium*		heteroaromatic substrates	(190,221−224)
PA0254 (HudA)	*Pseudomonas aeruginosa*	7ABN, 7ABO, 4IP2	heteroaromatic substrates	(224,225)
*An*InD	*Arthrobacter nicotinanae* FI1612	7P9Q	heteroaromatic substrates	(189,226)
PhdA (phenazine decarboxylase	*Mycobacterium fortuitum*		polyheteroaromatic substrates	(227)
*Pf*FDDC	*Paraburkholderia fungorum* KK1		heteroaromatic substrates	(228)

##### 3.2.3.3 Structure and (De)carboxylation Mechanisms

UbiD and its homologues catalyze an impressive range of (de)carboxylation reactions with different mechanisms. The better described decarboxylation mechanisms are outlined in detail below, including, (i) the decarboxylation of cinnamic acids catalyzed by Fdc type enzymes, which proceeds via a 1,3-dipolar cycloaddition,^25,191^ (ii) the *para*-decarboxylation of phenolic substrates featuring an intermediate which covalently links the prFMN cofactor and the quinoide substrate,^187^ and similarly, (iii) the decarboxylation of heteroaromatic carboxylic acids that proceeds *via* a substrate-prFMN intermediate formed in an electrophilic aromatic substitution, catalyzed by the HudA type enzymes.^224^

###### (i) 1,3-Dipolar Cycloaddition Catalyzed by Fdc Type Enzymes

**Figure 11:**
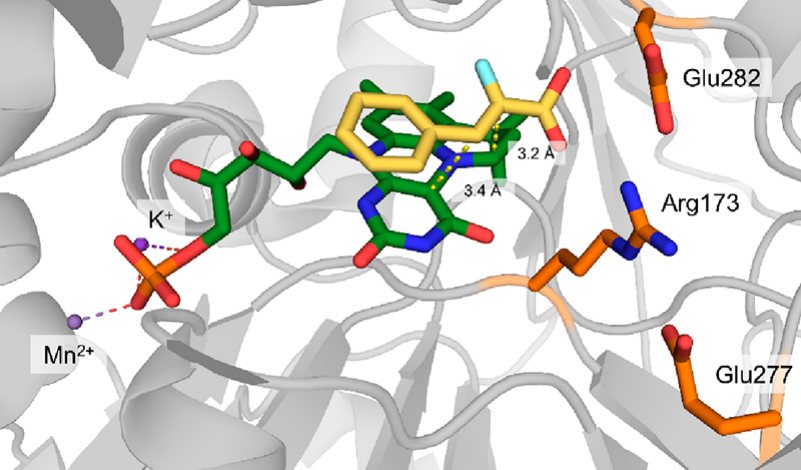
Crystal structure of the active site of Fdc1 from *A. niger* (PDB 4ZAB) with α-fluoro cinnamic acid (yellow). The substrate is positioned with its α-carbon near prFMN C1′ and with its β-carbon on top of prFMN C4a. The residues Arg173, Glu277, and Glu282 (orange) constitute the catalytic triad.

The (de)carboxylation of cinnamic acids catalyzed by Fdc type enzymes proceeds via a unique cycloaddition. First, the cinnamic acid substrate enters the active site with its carboxylic acid group pointing into the CO_2_/Glu282 binding pocket. The substrate’s Cα is located 3.2 Å away from the prFMN C1′ and the Cβ is in close proximity to the cofactor’s C4a (3.4 Å) (Figure 11).^211^

**Scheme 17:**
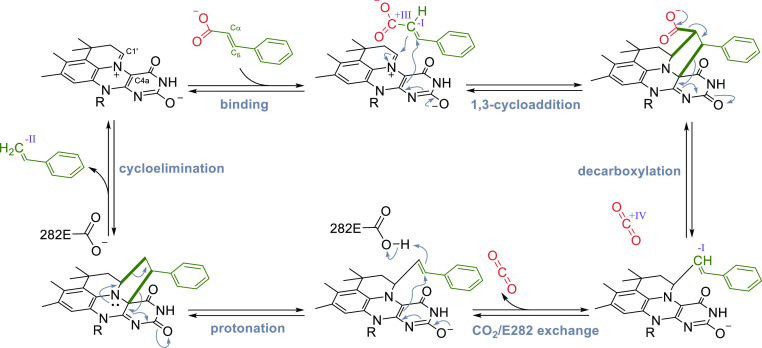
Proposed Reaction Mechanism for Ferulic Acid Decarboxylases Decarboxylating Cinnamic Acid-Type Substrates Based on a Dipolar 1,3-Cycloaddition

After correct positioning of the substrate, a 1,3-dipolar cycloaddition of the substrate and the prFMN occurs, thereby forming a new cycle (Scheme 17).^191^ Subsequent decarboxylation leads to ring opening and strain release. CO_2_ then leaves the active site and Glu282 can trigger protonation of the substrate leading to formation of another cyclic intermediate. Mutation of Glu282 to Gln causes loss of decarboxylation activity, while the Glu282Asp variant retains its activity. After protonation, the rate-limiting step, cycloelimination, happens using loss of ring strain as the driving force. Elimination results in product release and restoration of the prFMN cofactor.^25,203,215,229^ DFT calculations support the 1,3-dipolar cycloaddition mechanism and revealed α-hydroxycinnamic acid inhibition by keto–enol tautomerization.^213^

The highly conserved RX_n_EX_4_(E/D) motif among UbiD family members, constituting the catalytic triad (e.g., Arg173, Glu277, and Glu282 in *A. niger*) is displayed in Figure 11. The active site of Fdc was found to be highly complementary to the substrate–prFMN^iminium^ complex, causing the cycloadduct products to experience considerable strain, which guarantees fast progress in the reaction. Reducing this strain by mutagenesis resulted in decreased reaction rates.^25,203,229^

###### (ii) Nucleophilic Attack of Phenolic Substrates, Forming a Covalent Bond between the Substrate Quinoide and prFMN by AroY-Type Enzymes

**Scheme 18:**
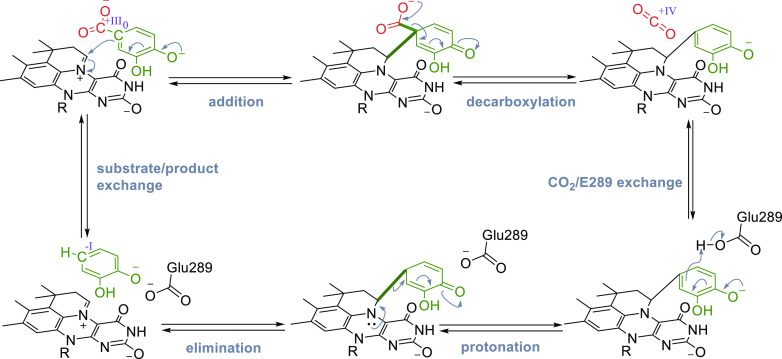
Proposed Reaction Mechanism for Decarboxylation of Phenolic Substrates by AroY-Type Enzymes

**Figure 12:**
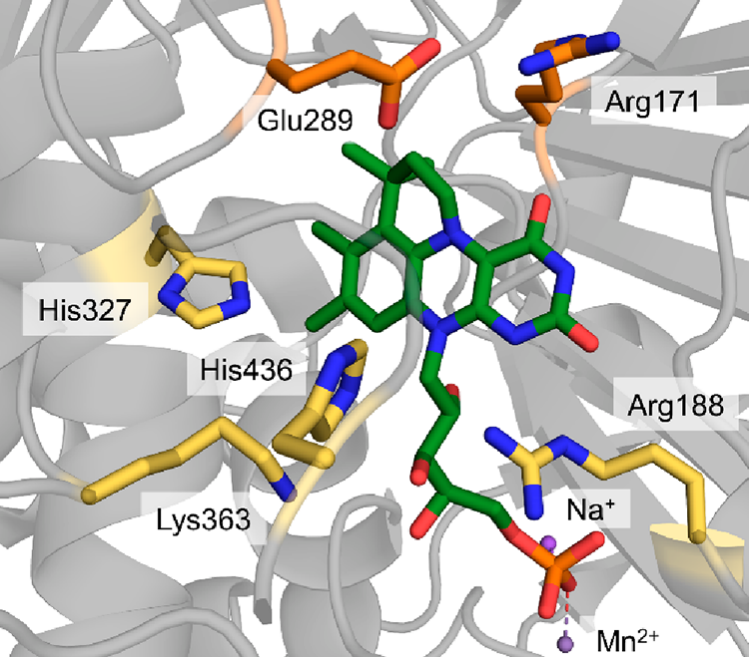
Crystal structure of EcAroY reconstituted with prFMN without substrate (PDB 5O3N). The amino acids marked in orange (Arg171, Glu289) contribute with hydrogen bonding to the substrate carboxylate moiety, and residues colored in yellow (Arg188, His327, Lys363, His436) are in hydrogen bonding proximity to the hydroxy group of the substrate according to DFT calculations.

In contrast to the dipolar cycloaddition, a distinct mechanism is proposed for AroY-type enzymes, involving a nucleophilic attack of the aromatic substrate on the C1′ of the prFMN. In this group of enzymes, a cycloaddition mechanism is unlikely due to the high strain that would be generated in the intermediate.^187^ In AroY, phenolic acid substrates are positioned in the active site with the substrate’s nucleophilic α-carbon on top of the isoalloxazine’s N5 atom. According to DFT calculations, Arg171 and Glu289 are in hydrogen bonding distance to the phenolic acid’s carboxylate moiety. In addition, the residues Arg188, His327, Lys363, and His436 are involved in hydrogen bonding interactions with the hydroxy functional group of the catechol-type substrates. The additional hydroxy group on the substrate aids its correct orientation and plays a pivotal role in increasing the nucleophilicity of the substrate’s α-carbon. After substrate binding, the reaction is proposed to proceed via nucleophilic attack to form a quinoid-like intermediate that is covalently bound to prFMN. This triggers decarboxylation, leading to a phenol intermediate that is covalently bound to the cofactor (Scheme 18). Finally, protonation, most likely mediated by Glu289 similar to the mechanism of Fdc from *A. niger*,^187^ leads to elimination of the substrate from the cofactor. Besides mutation of the key amino acid residues highlighted in Figure 12, also substitution of Leu438, Glu223, His189, and Phe183 results in loss of decarboxylating activity, suggesting their involvement in catalysis. Overall, this mechanism closely resembles an electrophilic aromatic substitution with CO_2_ being the leaving group.^187^

###### (iii) Nucleophilic Attack of Heteroaromatic Substrates on prFMN, via an Electrophilic Aromatic Substitution Catalyzed by the HudA Type Enzymes

**Scheme 19:**
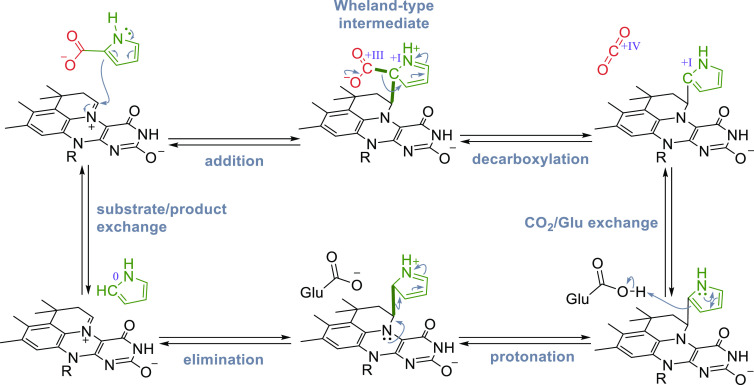
Proposed Electrophilic Aromatic Substitution for Decarboxylation via HudA-Type Enzymes

The pyrrole-2-carboxylate decarboxylase from *Pseudomonas aeruginosa* (HudA) accepts heteroaromatic compounds like pyrroles and furans as substrates. Decarboxylation is proposed to proceed via an electrophilic aromatic substitution on the pyrrole’s C2 carbon, forming a Wheland-type intermediate with the pyrrole ring oriented parallel to the prFMN^iminium^ plane (Scheme 19).^224^

**Figure 13:**
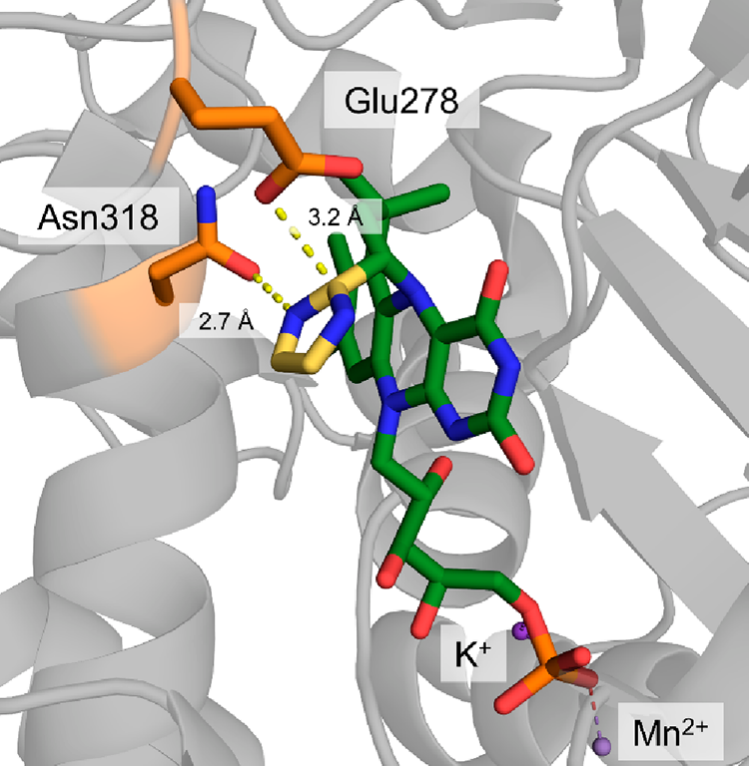
Crystal structure of pyrrole-2-carboxylic acid decarboxylase HudA from *Pseudomonas aeruginosa* with the reversible inhibitor imidazole bound in a covalent prFMN-imidazole adduct (PDB 7ABN). A key role is assigned to Glu278 and Asn318 in the decarboxylation of heteroaromatic compounds.

The enzyme was crystallized in two different conformations. While the apo-structure assumes an open conformation, cofactor and substrate binding induce structural changes leading to a more closed conformation. Similar results have been obtained in *An*Fdc.^224,230^ Imidazole is a competitive inhibitor, binding to residue Asn318 as seen in the crystal structure (Figure 13). Mutation of this residue to nonpolar amino acids causes a drop to very low conversions, which supposedly is a result of reduced cofactor binding.^231^ Apart from the above-mentioned *An*Fdc and *Pa*HudA crystal structures, most UbiD enzymes have been crystallized in the “open” state. In the case of *An*InD, a hexameric light and oxygen sensitive indole-3-carboxylate decarboxylase from *Arthrobacter nicotinae*, small-angle X-ray scattering (SAXS) investigations revealed the existence of several open and closed conformations in solution.^224^

##### 3.2.3.4 Substrate Scope

The most studied prFMN-dependent enzymes are fungal ferulic acid decarboxylases, namely the orthologue originating from *Aspergillus niger* (*An*Fdc) and its homologue from *Saccharomyces cerevisiae* (*Sc*Fdc), both able to catalyze the decarboxylation of structurally and electronically diverse styrenes. In contrast to phenolic acid decarboxylases (PADs), Fdcs do not require a *p*-hydroxy moiety, and substrates with weak electron-withdrawing and electron-donating substituents are generally well accepted. In addition to cinnamic acid derivatives, unsaturated aliphatic carboxylic acids are decarboxylated by Fdcs. Leys et al.^212^ summarized the structural substrate requirements to be an acrylic acid functionality connected to an expanded π-system, as for instance (hetero)aromatic moieties or further double bonds. In addition, the presence of strongly electron-donating groups decreases reaction rates and the (*E*)/(*Z*)-configuration of the unsaturated C=C bond has an influence on the outcome.^212^

**Scheme 20:**
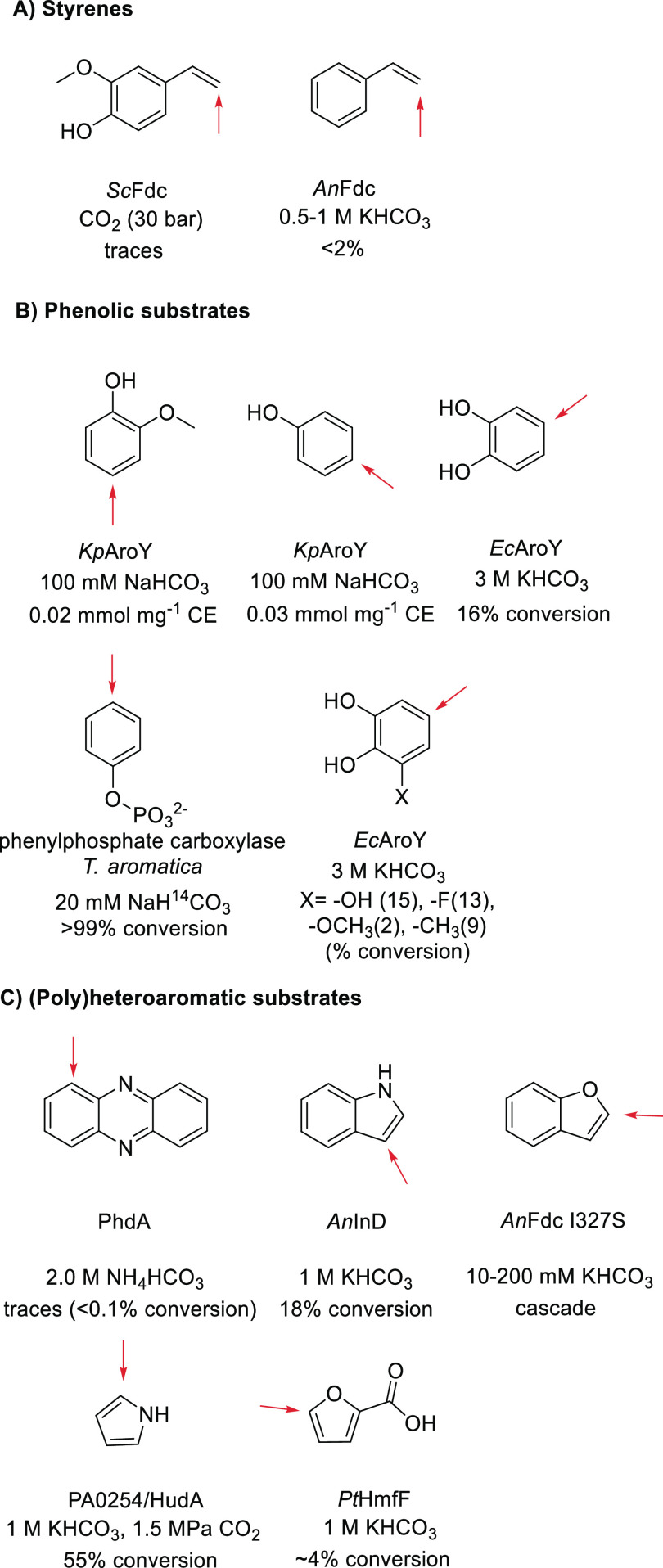
Substrates That Were Accepted for Carboxylation by prFMN-Dependent Enzymes Red arrows indicate the position of carboxylation.^80,187,189,211,212,218,219,224,227^ For each substrate the carboxylating enzyme, the carboxylating agent and the results in terms of carboxylation activity indicated by conversion are shown (see also Table 4).

Several prFMN-dependent enzymes have been reported also to be able to run the reaction in the reverse carboxylation direction in the presence of an appropriate carbon dioxide source at elevated concentrations. A variety of aromatic compounds are accepted as substrates which are divided in: (A) styrene-type,^211,212,214,217,232^ (B) phenol-type,^80,187,188,206,218,219,228,233−235^ and (C) heteroaromatic substrates (Scheme 20).^190,221,223,224,227^ However, except for the carboxylation of the activated phenylphosphate substrate catalyzed by *T. aromatica* phenylphosphate carboxylase, which was well tolerated by the enzyme (99% conversion), only poor to moderate results in terms of carboxylation power were observed.

#### 3.2.4 TPP-Dependent Keto Acid Decarboxylases

**Scheme 21:**
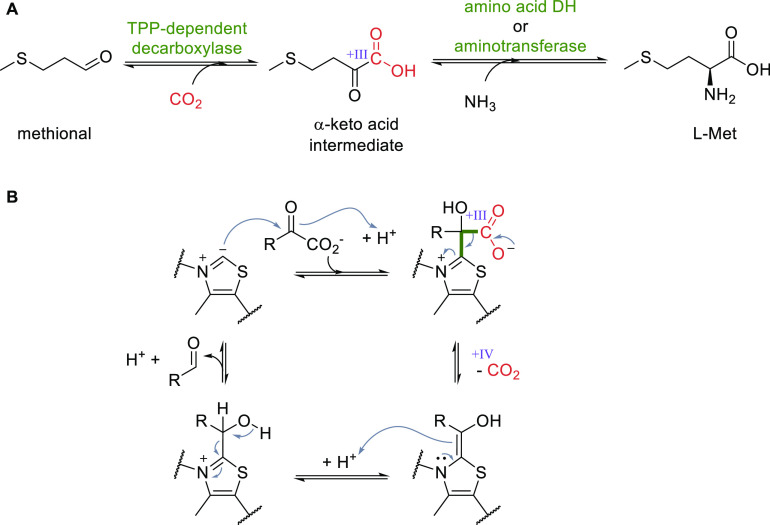
Application of TPP-Dependent Decarboxylases for Carboxylations (A) Two-step enzymatic cascade towards the synthesis of L-Met starting from methional via a carboxylation and subsequent amination step. (B) Catalytic cycle of the decarboxylation of keto acids by TPP-dependent decarboxylases.

TPP-dependent keto acid decarboxylases such as pyruvate decarboxylase Pdc1 from *Saccharomyces cerevisiae*,^148,236^ phenylpyruvate decarboxylase Aro10 from *Saccharomyces cerevisiae*,^237^ and the branched-chain decarboxylase KdcA from *Lactococcus lactis*(238) represent further examples of catabolic enzymes that are utilized as biocatalysts for the synthesis of valuable, industrially relevant products. By simply reversing the Ehrlich pathway, which in nature is responsible for the degradation of various amino acids,^239^ the latter are synthesized, starting from aldehydes. l-Methionine (L-Met) besides other amino acids (l-Leu, l-Ile) was obtained via a two-step enzymatic cascade.^39^ In the case of L-Met, the cascade is initiated by the carboxylation of methional, to yield the corresponding α-keto acid intermediate, followed by a subsequent amination step catalyzed by either an amino acid dehydrogenase or aminotransferase (Scheme 21A). The application of pressurized gaseous CO_2_ (∼2 bar) and the pull by the amine-forming enzyme are required to move the equilibrium of the energetically-unfavored carboxylation to the product side.^39^Scheme 21B displays the TPP-dependent mechanism for the natural decarboxylation reaction, involving an attack of the TPP on the keto acid, decarboxylation, protonation of the formed enol, and finally cleavage of the aldehyde from TPP. For details regarding the cascade, refer to section 4.3.^39^

Furthermore, pyruvate decarboxylase from *Saccharomyces cerevisiae* was used for the carboxylation of acetaldehyde (100 μM) to yield pyruvic acid (up to 81% conversion) using bicarbonate (500 mM, sodium carbonate buffer) as carboxylating source.^148^

#### 3.2.5 Decarboxylases from Tannin Degradation

**Scheme 22:**
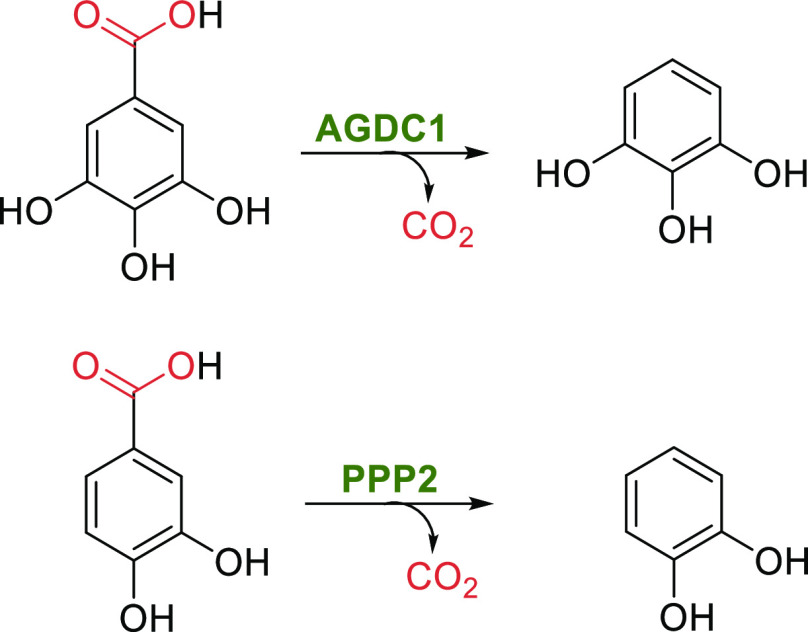
Decarboxylation of Gallic Acid and Protocatechuic Acid by the Two Fungal Enzymes AGDC1 and PPP2^147^

Very recently, a new class of decarboxylases was discovered, which does not depend on a cofactor (compare the PADs discussed in section 3.2.2). The enzymes are involved in tannin degradation, converting gallic acid and protocatechuic acid to pyrogallol and catechol, respectively (Scheme 22).^147^ AGDC1 from *Arxula adenivorans* and PPP2 from *Madurella mycetomatis* both form trimers and do not require any organic cofactor. Instead, the enzymes only rely on acid–base catalysis that facilitates the stabilization of the reaction’s transition state. Each trimer contains one potassium ion, coordinated 3-fold by the Glu88-residue of each monomer, overall forming a distorted octahedral coordination.^147^ Although this new group of nonoxidative decarboxylases remains to be investigated for carboxylation reactions, it holds great potential to further expand the substrate scope accessible to carboxylases.

### 3.3 Reaction Engineering of Enzymatic Carboxylation Reactions

Many of the discussed enzymatic carboxylation reactions suffer from low productivities and incomplete conversions, mostly due to the high amount of energy that is required to utilize CO_2_ (vide supra). Nature overcomes this issue by using cofactors to provide reaction energy, such as NAD(P)H and ATP (compare section 2).^14^ However, in case ATP- or NAD(P)H-dependent enzymes are applied in biotechnology or synthesis, recycling of the costly cofactors by dedicated cofactor regeneration systems, either in vivo or in vitro, becomes crucial to render the process economically feasible. In contrast, the equilibrium of cofactor-independent carboxylases is often shifted to the product side by supplying the CO_2_-source (often bicarbonate) in large excess (compare section 3.3.1).^10,12^ While the strategies for pushing the equilibrium of enzymatic carboxylation reactions are discussed in a recent review in great detail,^10^ this section aims to give a more general overview of such methods and puts them in the context of reaction engineering.

**Scheme 23:**
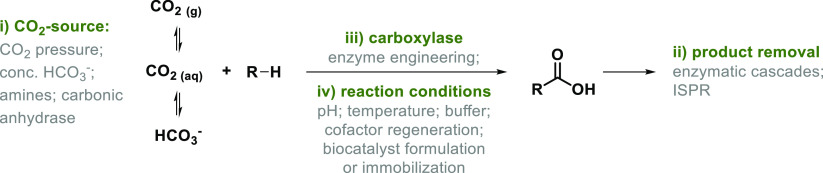
Reaction Engineering of Biocatalytic Carboxylation Processes

In general, enzymatic carboxylations offer several opportunities for reaction engineering (Scheme 23), including (i) supply of CO_2_ in order to push the equilibrium, (ii) removal of the carboxylation product from the reaction (equilibrium) to exert a pull force onto the reaction, e.g., via the addition of further enzymes in a cascade reaction (compare section 4.1) or in situ product removal methods (ISPR), (iii) improving the catalyst itself via enzyme engineering or immobilization, and (iv) optimization of reaction conditions such as pH, temperature, buffer composition, and other related parameters, including the regeneration of cofactors.

#### 3.3.1 CO_2_ Source

For many carboxylases and reversed decarboxylases, it is still unclear whether dissolved CO_2 (aq)_, or bicarbonate is utilized by the enzyme.^9,12^ As the different species contributing to the total CO_2_ concentration (CO_2 (total)_) are in equilibrium, the identification of the carboxylation cosubstrate is not straightforward and often computational methods in combination with kinetic measurements are used to investigate which species initially binds in the active site.^29,30,48^ For carboxylations, reaction rates are often measured at different bicarbonate concentrations and compared to results obtained under CO_2_ atmosphere at different pressures.^39,48,163^ However, due to the fast interconversion of the different species contributing to CO_2(total)_ (i.e., CO_2 (g)_, CO_2 (aq),_ HCO_3_^–^, CO_3_^2–^, cf. Scheme 23), such experiments often lead to ambiguous results. For reaction engineering, the spontaneous interconversion of the different CO_2_ species is beneficial, as it enables both bicarbonate and CO_2(g)_ to be used as carboxylation agent.

Bicarbonate is usually used in concentrations greater than 1 M (up to saturation at approximately 3 M) (refs (9, 148, 152, 162, 164, 166, and 187) ). Increasing the concentration of bicarbonate has been shown to improve the free energy of the transformation.^29^ Evaluation of different bicarbonate sources revealed similar performance, as long as the counterion is not too chaotropic in the Hofmeister series. Following this trend, K^+^, Na^+^, Cs^+^, bicarbonate based ionic liquids, and quaternary amines such as NH_4_^+^ or choline are well accepted by bivalent metal dependent decarboxylases and phenolic acid decarboxylases.^159,166^

An attractive option to supply a carboxylation reaction with CO_2_ is adding it directly as gas, either by applying pressure or a gas stream.^9,10,12,39^ Increasing CO_2_ pressure goes in hand with an increased CO_2_ solubility (e.g., 0.04 mol L^–1^ at 1 bar, 0.7 mol L^–1^ at 25 bar; 1.13 mol L^–1^ at 50 bar, and 1.5 mol L^–1^ at 100 bar, all at 20 °C),^240^ while higher temperatures lead to a decreased solubility (e.g., 0.9 mol L^–1^ at 12.4 °C, 0.7 mol L^–1^ at 20 °C, 0.6 mol L^–1^ at 31.04 °C, 0.5 mol L^–1^ at 40 °C, all at 25 bar).^240^ Note that different concentrations of CO_2_ lead to varying concentrations of bicarbonate, resulting in a change of pH at different pressures. While some carboxylases have been reported to be irreversibly deactivated at higher pressure,^163^ others tolerate pressures greater than 80 bar,^159,187^ which allows application of supercritical CO_2_ for carboxylations.^10,48,79,241−243^

Methods applied for CO_2_ capture and storage can be used to increase the effective concentration of CO_2_ in solution for biocatalytic applications. For example, aqueous medium can be exchanged or supplemented by solvents that dissolve higher concentrations of CO_2_ (e.g., 2-(isopropylamino)ethanol and 2-amino-2-methyl-1-propanol).^166,241^ Alternatively, amines can be used to capture CO_2_ in the form of ammonium bicarbonate salts. A range of different primary-, secondary-, and tertiary amines have been applied to sequester CO_2(g)_ serving as substrate for 2,3-DHBD_*Ao*, a bivalent metal-dependent decarboxylase.^159^ Carbon dioxide was provided either under pressure (50 bar) or at atmospheric pressure via bubbling aeration. In both cases, the sequestering amine was added in a concentration of up to 1 M.^159^ In a follow up study, the system was further extended to supplying CO_2_ as very fine bubbles using small diameter spargers in the presence of 3 M triethylamine for CO_2_ sequestration. This method provides CO_2_ with a high volume-specific surface area and therefore increases mass transfer, which allowed reaching of 26% conversion of catechol to the corresponding carboxylate, using the enzyme 2,6-DHBD_*Rs* and a substrate loading of 80 mM.^175^ An attractive alternative to batch carboxylation reactions, is running the reactions in continuous flow. Similar to batch, it is possible using this technology to provide CO_2_ at high pressure. In an exemplary process, pyrrole was carboxylated to pyrrole-2-carboxylate at a pressure of 65 bar and a flow rate of 1.5 mL min^–1^, using the immobilized carboxylase from *B. megaterium* (PYR2910). The final process achieved a space-time yield of 24 ± 7 μmol h^–1^, which is a 25-fold improvement over the corresponding batch protocol.^223^ In another example, utilizing immobilized RuBisCO, HCO_3_^–^ was applied as carbon source in continuous flow.^244^

Carbonic anhydrases (CAs) belong to the fastest known enzymes and accelerate the interconversion of bicarbonate and CO_2_ from approximately 10^–2^ s^–1^ (spontaneous) to a turnover frequency of up to 10^6^ s^–1^.^23^ This is beneficial for both enzymes utilizing CO_2_ and enzymes utilizing bicarbonate, as the concentrations of the preferred carboxylation source is kept constant due the fast CA catalyzed equilibration. While CA is found in many organisms, functioning for example in pH regulation, CA has a particularly interesting function in photosynthetic bacteria. There, CA is colocalized in the carboxysome, a carbon concentration mechanism that evolved to increase the local CO_2_ concentrations around RuBisCO, thereby effectively suppressing the oxygenase side reaction.^245^ For enzymatic applications, CAs have been used to improve the enzymatic reduction of CO_2_ to formate using formate dehydrogenases,^246,247^ they improved the carboxylation activity of a phosphoenolpyruvate carboxylase,^248^ and they are even beneficial in multienzymatic systems that convert carbon dioxide to methanol.^249,248^ In contrast, only little effect was found, when CA was combined with bivalent metal-dependent decarboxylases, suggesting that the availability of the mechanistically relevant carboxylation source was not rate determining in this setup.^10,152^

#### 3.3.2 Product Removal

An efficient way to exert an external driving force onto an enzymatic reaction system is the removal of the formed product by an additional (irreversible) reaction. Owing to the fact that biocatalysts usually require similar reaction conditions, the addition of a subsequent enzymatic step to a carboxylation reaction is often straightforward and mirrors nature’s CO_2_ fixation strategies, where carboxylation reactions are part of biosynthetic pathways or cycles.^10,12,15,137,250−256^ The efficiency of a reaction can be evaluated from a thermodynamic point of view by estimating the Gibbs free energy demand of the individual reactions. Dedicated tools allow to calculate the Δ*G* of small cascades and even entire pathways at physiological or process conditions and therefore to evaluate their thermodynamic feasibility.^42^

Enzymatic reactions that are applied for product removal either directly functionalize the newly formed carboxylate moiety or derivatize one of the product’s substituents that is mechanistically relevant in the (de)carboxylation.^10,137,253,255^ An example for the first case is the application of carboxylic acid reductases and other enzymes for the further conversion of cinnamic acids or aromatic acids, that were produced via carboxylation reactions catalyzed by ferulic acid decarboxylase from *Aspergillus niger* (*An*Fdc) and its variants.^211^ The removal of the produced carboxylic acids from the equilibrium via this cascade allowed reduction of the required excess of bicarbonate and to overall increase the conversations from below 15%^187^ (without the cascade) to conversions that are greater than 90%.^211^ An example for the latter case is methionine synthesis by a sequence of carboxylation and amination. Carboxylation is performed using a TPP-dependent branched-chain decarboxylase, and amination is catalyzed by a transaminase or an amino acid dehydrogenase. The amination of the α-keto moiety of the carboxylation product converts it into a nonsubstrate for the decarboxylase, making the reaction irreversible and yielding the desired methionine in 40% conversion.^39^ Similar processes are further discussed in section 4.3.

Alternatives to the mentioned enzymatic systems are chemical derivatization or scavenging of the carboxylation products, i.e., by ISPR. One option is the use of dedicated adsorbents for binding/desorption cycles of the carboxylation products.^161^ Such a system has been developed using the anion exchange resin Dowex 1 × 2 (Cl) for adsorption of 2,6-dihydroxy-4-methylbenzoic acid produced via carboxylation of orcinol, using the decarboxylase from *Aspergillus oryzae* (2,3-DHBD_*Ao*). The method was used at a 400 mL scale and produced 878 mg of highly pure product requiring no additional purification steps.^161^

Chemical modification of carboxylation products was required to assay the off-equilibrium acetyl-CoA carboxylation activity of the PFOR from *Desulfovibrio africanus* and *Sulfolobus acidocaldarius*.^47^ The energetic barrier for carboxylation was overcome by derivatization of the generated pyruvate with semicarbazide, forming pyruvate semicarbazone.^47^ The reduced ferredoxins required by PFOR as electron carrier, were regenerated using a photobiocatalytic system.

An alternative ISPR was developed by capitalizing on the fact that some benzoic acid derivatives form insoluble salts with quaternary ammonium counterions.^257^ Thus, these salts can be used to precipitate the carboxylation products and therefore remove them from the reaction equilibrium. A systematic evaluation of different quaternary ammonium salts for the precipitation of 2,4-dihydroxybenzoic acid via carboxylation of resorcinol at 10 mM concentration, identified tetrabutylammonium bromide as an ideal supplement. Adding 50 mM of this salt, allowed increasing of the conversion from 37% (no salt) to 97% using the decarboxylase 2,6-DHBD_*Rs*. Interestingly, precipitation is selective for the target product acid and the regio-isomer was not found.^257^ However, choosing the precipitation agent is nontrivial, as different benzoic acids require different ammonium salts. For example, in contrast to 2,4-dihydroxybenzoic acid, precipitation of 1,2-dihydroxybenzoic acid required dodecyldimethylbenzylammonium counterions.^257^ Importantly, product precipitation can also be combined with other methods to increase conversion. Combining tetrabutylammonium bromide as precipitation agent, together with trimethylamine for CO_2_ sequestration, allowed increase the carboxylation yield of an 80 mM solution of resorcinol from 7% to 43%. However, elevated triethylamine concentrations also increased the solubility of the precipitated salts requiring further reaction optimization.^257,258^

#### 3.3.3 Engineering of the Biocatalyst

One of the most straightforward methods to improve a biocatalytic process is direct engineering of the catalyst itself. However, enzyme engineering can only change the kinetics of the catalyzed reaction and has no effect on the reaction’s thermodynamics. Especially carboxylation reactions often reach equilibrium and enzyme engineering cannot influence conversion beyond this point, as the forward and reverse reactions are equally fast. However, generating variants of carboxylases allowed to increase their stability, activity, and to broaden their substrate scope. The most prominent example by far is RuBisCO, as a tremendous amount of mutational studies, including directed evolution approaches, have targeted the enzyme with the goal to increase its activity or its specificity toward CO_2_.^9,49,259,260^ Besides acceleration of their catalytic function, the substrate scope of enzymes can be altered using enzyme engineering. In an impressive example, a minimal carboxylation activity of a biotin-dependent propionyl-CoA carboxylase from *Methylorubrum extorquens* toward glycoyl-CoA was increased more than 50-fold by rational design paired with directed evolution.^55^ Likewise, a double mutation of the enzyme SAD_*Tm* (Y64T-F195Y) fine-tuned the active site for better binding and higher activity toward the substrate *para*-aminosalicylic acid (cf. Scheme 13).^169,170^ In some cases, enzymes that previously exhibited completely different functions can be modified to perform carboxylation reactions. Recently, the latent carboxylation activity of the propionyl-CoA synthase from *Erythrobacter* sp. NAP1, as well as the promiscuous carboxylase activity of an acrylyl-CoA reductase from *Nitrosopumilus maritimus*, were improved to synthetically relevant levels using rational design.^46^

Studies such as the latter one underline that rational engineering involving careful studies of the enzyme’s mechanism and structure still is an efficient way to create beneficial variants. However, high-throughput and computational methods are on the verge of being broadly applicable and will solve many challenges in enzyme engineering.^261−264^ Note, that within this review, the most important variants of the individual enzyme classes are discussed in their respective subsections.

#### 3.3.4 Optimization of the Reaction Conditions and the Formulation of the Biocatalyst

Identification of the ideal reaction conditions is crucial, especially for enzymatic reactions, as the operational window of enzymes is usually quite narrow, i.e., biocatalysts often do not tolerate extreme temperatures, solvent concentrations, pH values, or pressure. In case several enzymes are combined into a reaction cascade, either a good compromise of the individual catalysts preferred reaction conditions has to be found, or the cascade is performed in a stepwise fashion, by sequential addition of catalysts (see section 4.2). Besides the obligatory checks for the ideal reaction temperature, pH, and buffer composition, some parameters are of special interest for carboxylases, including the supply and form of the carboxylation source (for details, see discussion in section 3.3.1).

As the formulation of the biocatalyst significantly affects the applicable reaction conditions, this parameter must be especially considered. Enzymes can be provided as living cells (in vivo), as purified enzymes, or in any formulation in between, including their application as resting cells, lyophilized whole cells and cell-free extracts. By using methods of immobilization, enzymes can be covalently or noncovalently attached to a solid support. These different formulations affect the catalyst’s stability (total turnover numbers, e.g. at different temperatures or levels of cosolvent) and activity (turnover number).

Decarboxylases are often produced in *E. coli* and applied as cell-free lysates. If the cell’s background does not interfere with the catalyzed reaction, purification is only required for mechanistic investigations and kinetic experiments.^11,12^ For more complex systems, such as artificial synthetic pathways, purified enzymes are used in most cases in order to minimize side reactivities and to allow for careful balancing of the individual enzymatic activities.^137,253−255^ As a special case, enzymes from the UbiD family depend on the prenylated FMN cofactor. Their heterologous expression requires a host that is able to catalyze formation and maturation of this cofactor (e.g., *E. coli*).^187^

Whereas the immobilization of CA has been applied in larger scale,^23^ only few carboxylases have been immobilized yet. Immobilization often increases enzyme stability, but more importantly, it also allows enzyme recovery and reuse by simple filtration. Immobilization strategies range from covalent immobilization over the use of adsorbents to encapsulation/entrapment methods.^265−267^ However, the outcome of a specific immobilization method is often unpredictable, and different immobilization conditions and methods need to be evaluated empirically. For example, for the immobilization of the Mn^2+^-dependent phenylphosphate carboxylase from *Thauera aromatica*, several supports, including zeolites, pumice, and polyacrylamide, were tried unsuccessfully. Finally, using low-melting agar as support yielded an active preparation that was stable for more than one week.^18^ The carboxylase from *B. megaterium* (PYR2910) was adsorbed onto a polyallylamine ion-exchange resin to allow its application as stationary phase in a continuous flow process.^223^ Another example is the immobilization of a acetyl-coenzyme A carboxylase on sepharose.^268^ RuBisCO from spinach leaves was covalently immobilized on a nylon membrane and on agarose^269^ or on polydopamine and further applied in a microfluidic reactor.^244^ Also, the coimmobilization of carboxylases, together with other enzymes forming an enzymatic cascade, has been explored. The cascade, catalyzing the formation of ribulose 1,5-bisphosphate either from ribose-5-phosphate or glucose, and its subsequent carboxylation by RuBisCO, was immobilized on self-assembled synthetic amphiphilic peptide nanostructures.^270^ In another example, CA and phosphoenolpyruvate decarboxylase covalently coimmobilized on microbeads were applied to produce oxaloacetate from PEP and could be reused up to 20 times without a reduction of activity.^271^ Similar coimmobilization strategies have been repeatedly applied to entrap FDH together with other enzymes to form enzymatic cascades for converting CO_2_ to methanol.^272,273^ Immobilization methods also allow the linkage of redox enzymes to electrodes, facilitating direct electron transfer. In one study, ferredoxin-NADP^+^ reductase from *Synechococcus* sp. and crotonyl-CoA carboxylase/reductase from *Methylobacterium extorquens* were coimmobilized on a glassy carbon electrode in a viologen-modified hydrogel.^274^ Viologen facilitates electron transfer from the electrode to the ferredoxin, which regenerates NADPH that in turn is consumed by the carboxylase. This system, producing ethylmalonyl-CoA from crotonyl-CoA, reached a total turnover number of 117.^274^

The supplementation of the reaction medium with cosolvents, such as organic solvents, ionic liquids, or other additives, can have a positive effect on the reaction rates and the enzyme stability.^166,181,241,275^ Interestingly, the use of dedicated CO_2_-capture solvents is not necessarily beneficial.^166^ Therefore, the identity and amount of the supplied solvent needs to be empirically optimized for individual enzymes.

Many classes of carboxylation enzymes require stoichiometric amounts of cofactors, such as ATP or NAD(P)H. For example, the activation of carboxylic acids as CoA thioesters is an ATP-dependent process that produces the substrates for enoyl-thioester reductases/carboxylases or glycolyl-CoA carboxylase.^9,10,12,14^ To avoid consumption of stoichiometric amounts of the costly cofactors, regeneration systems can be implemented.^276,277^ Besides traditional coupled enzyme systems, also whole cells, electrochemical methods, or even photosynthetic membranes encapsulated in microfluidic droplets were shown to drive regeneration of ATP and NADPH.^274,278^

## 4 CO_2_ Fixation Pathways and Cascades

CO_2_ is freely diffusible in air and soluble in water. Therefore, its concentration is stable in most environments with very little diurnal or seasonal fluctuations. Its constant availability makes CO_2_ an attractive carbon source, especially for sessile organisms such as plants. As a result, nature has developed several, evolutionarily independent ways to utilize CO_2_ as a carbon source.

To date, seven natural autotrophic carbon fixation pathways have been elucidated in detail: the Calvin–Benson–Bassham (CBB) cycle,^279^ the reverse TCA (rTCA)/reverse oxidative TCA (roTCA) cycle,^280−282^ the Wood-Ljungdahl (WL) pathway,^283^ the reductive glycine pathway,^62,284^ the dicarboxylate 4-hydroxybutyrate (dicarboxylate) cycle,^285^ the 3-hydroxypropionate 4-hydroxybutyrate (3HP/4HB) cycle,^286^ and the 3-hydroxypropionate (3HP) bicycle.^287^ Although these pathways have been extensively reviewed,^256,288,289^ we want to give a concise overview serving as a starting point for subsequent discussions.

In addition to the naturally evolved carbon fixation pathways, several synthetic cascades have been developed that rely on carboxylases as key enzymes. Herein, we differentiate these systems into cascades that produce key cellular metabolites from CO_2_ (section 4.2) and into cascades that in contrast utilize the synthetic potential of (de)carboxylases to produce fine chemicals (section 4.3).

Synthetic carbon fixation pathways are designed as alternative to natural pathways. Very often, synthetic pathways aim to kinetically or thermodynamically outcompete natural pathways or provide other advantages such as ease of incorporation into host strains or oxygen tolerance. While some of them have been successfully demonstrated in vitro, they ultimately need to be transferred into in vivo systems to prove that they can sustain life. Like their natural counterparts, artificial CO_2_ fixation pathways often have a cyclic topology, are typically complex, and consist of many enzymes (typically more than ten different enzymes). The successful realization of these systems therefore requires careful planning, selection, and characterization of the biocatalysts, as well as optimization of their interplay. Section 4.2 summarizes the current state of methodologies to overcome this challenge. Synthetic cascades for CO_2_ utilization in contrast focus on the addition of CO_2_ as C1 building block to a target molecule and mostly consist of two to five enzymes. Because such systems are often linear and less complex, typically higher productivities and concentrations are achieved.

For a comprehensive overview, both natural and synthetic CO_2_ fixation pathways are listed in Table 5 (further details can be found in the Supporting Information). Synthetic CO_2_ utilization pathways are summarized in Table 6.

**Table 5:** Overview of Natural and Synthetic CO_2_ Fixation Cycles^a^

pathway	primary product	normalized product	ATP	NAD(P)H	FADH2	Fdx^2–^	ATP eq/CO_2_ (acetyl-CoA)	Δ_r_*G*,°^0^ (kJ/mol/)/CO_2_(acetyl-CoA)	O_2_ sensitivity	status	ref
CBB cycle	glyceraldehyde-3P	acetyl-CoA	7	4	0	0	8.5	–102.8 ± 6.4	no, but side reactivity	natural	(279)
reverse (o)TCA	acetyl-CoA	acetyl-CoA	2(1)	2	1	1	5.5(5)	–34.3 ± 13.3 (−19.5 ± 13.3)	yes	natural	(256,281,282,290)
WL pathway (acetogens)	acetyl-CoA	acetyl-CoA	1	2	0	2	5.5	–27.2 ± 13.0	yes	natural	(291)
rGlycine^b^	pyruvate	acetyl-CoA	2	2	0	2	6	–42.0 ± 13.0	yes (if using Fdx for FDH)	natural	(62,113,292)
DC cycle	acetyl-CoA	acetyl-CoA	5	2	1^c^	1	7	–72.4 ± 8.9	yes	natural	(285)
3HP/4HB cycle	acetyl-CoA	acetyl-CoA	4	4	0	0	7	–58.5 ± 6.2	no	natural	(286)
3HP bicycle	pyruvate	acetyl-CoA	5	5	–1	0	8	–81.3 ± 7.0	no	natural	(287,293)
CETCH 5.4	glyoxylate	acetyl-CoA	2	8	–4	0	8	–59.7 ± 13.5	no	synthetic (in vitro)	(137,254,278)
HOPAC	glyoxylate	acetyl-CoA	6	6	–2	0	9	–103.5 ± 8.8	no	synthetic (drafted)	(137)
rGPS-MCG	acetyl-CoA	acetyl-CoA	5	5	–1	0	8	–80.9 ± 7.0	no	synthetic (in vitro)	(294)
POAP	oxalate	acetyl-CoA	6	3	0	1	8	–94.6 ± 8.5	yes	synthetic (in vitro)	(253)
GED	glyceraldehyde-3P	acetyl-CoA	7	4	0	0	8.5	–102.8 ± 6.4	no	optimized in host	(70)

a CBB, Calvin–Benson–Bassham; reverse (o)TCA cycle refers to both the reverse TCA cycle employing citrate-ATP lyase and the reverse oxidative TCA cycle employing citrate synthase in the reverse reaction. Values for the reverse oxidative TCA cycle are listed in parentheses. WL, Wood–Ljungdahl; rGlycine, reverse glycine; DC, dicarboxylate/4-hydroxybutyrate; 3HP/4HB, 3-hydroxypropionate/4-hydroxybutyrate; 3HP, 3-hydroxypropionate bicycle; CETCH 5.4, crotonyl-CoA/ethylmalonyl-CoA/hydroxybutyryl-CoA cycle version 5.4; HOPAC, hydroxypropionyl-CoA/acrylyl-CoA cycle; rGPS-MCG, reductive glyoxylate and pyruvate synthesis and malyl-CoA-glycerate cycle; POAP, PYC-OAH-ACS-PFOR cycle; GED, Gnd–Entner–Doudoroff cycle.

b Ferredoxin is assumed to be the electron donor for formate dehydrogenase in this pathway.

c Fumarate reductase in the dicarboxylate cycle uses FADH_2_ as electron donor. Fdx, ferredoxin; FDH, formate dehydrogenase. For ATP per CO_2_ conversions, a P/O ratio of 2.5 for Fdx^2–^, 2.5 for NAD(P)H, and 1.5 for FADH_2_ was assumed. Products were normalized for the production of acetyl-CoA via conversions presented in Schwander et al.^137^ and in Supporting Information, Table 1. Fdx^2–^ refers to the use of two separate single electron-transferring ferredoxins (i.e., a 2-electron reduction).

**Table 6:**
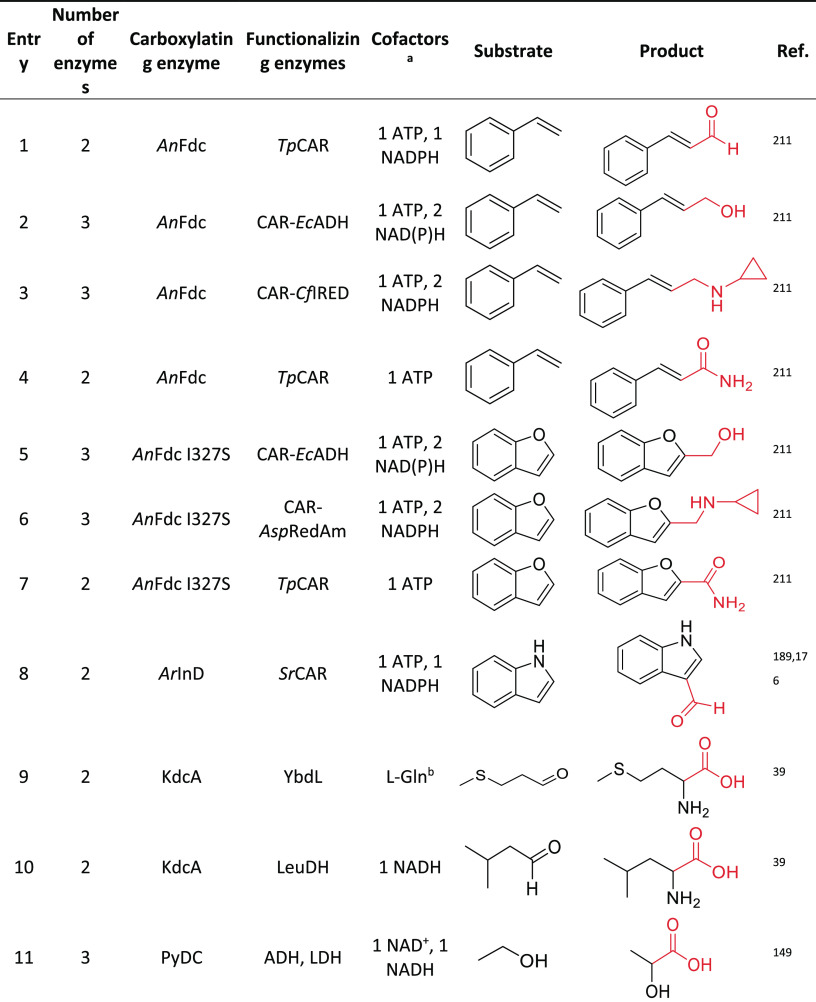
Overview of Biocatalytic Cascades for Synthetic CO_2_ Utilization^a^

a AnFdc, ferulic acid decarboxylase from *Aspergillus niger*; CAR, carboxylic acid reductase; EcADH, alcohol dehydrogenase from *Escherichia coli*; CfIRED, imine reductase from *Cystobacter ferrugineus*; TpCAR, carboxylic acid reductase from *Tsukamurella paurometabola*; AspRedAm, reductive aminase from *Aspergillus oryzae*; SrCAR, carboxylic acid reductase from *Segniliparus rugosus*; ArInD, indole-3-carboxylic acid decarboxylase from *Arthrobacter nicotianae*; PyDC, pyruvate decarboxylase; LDH, lactate dehydrogenase; KdcA, decarboxylase from *Lactococcus lactis*; YbdL, methionine aminotransferase from *Escherichia coli* K12; LeuDH, leucine dehydrogenase from *Lysinibacillus sphaericus* ATCC 4525; PEPC, phosphoenolpyruvate carboxylase; CA, carbonic anhydrase.

b As none of the carboxylating enzymes in this table require either ADP, NAD(P)H, or Fdx, the cofactor equivalents required in the full cascade are given.

c One equivalent of l-glutamine is required as amino donor by the aminotransferase.

### 4.1 Natural CO_2_ Fixation Pathways

#### 4.1.1 CBB Cycle

**Figure 14:**
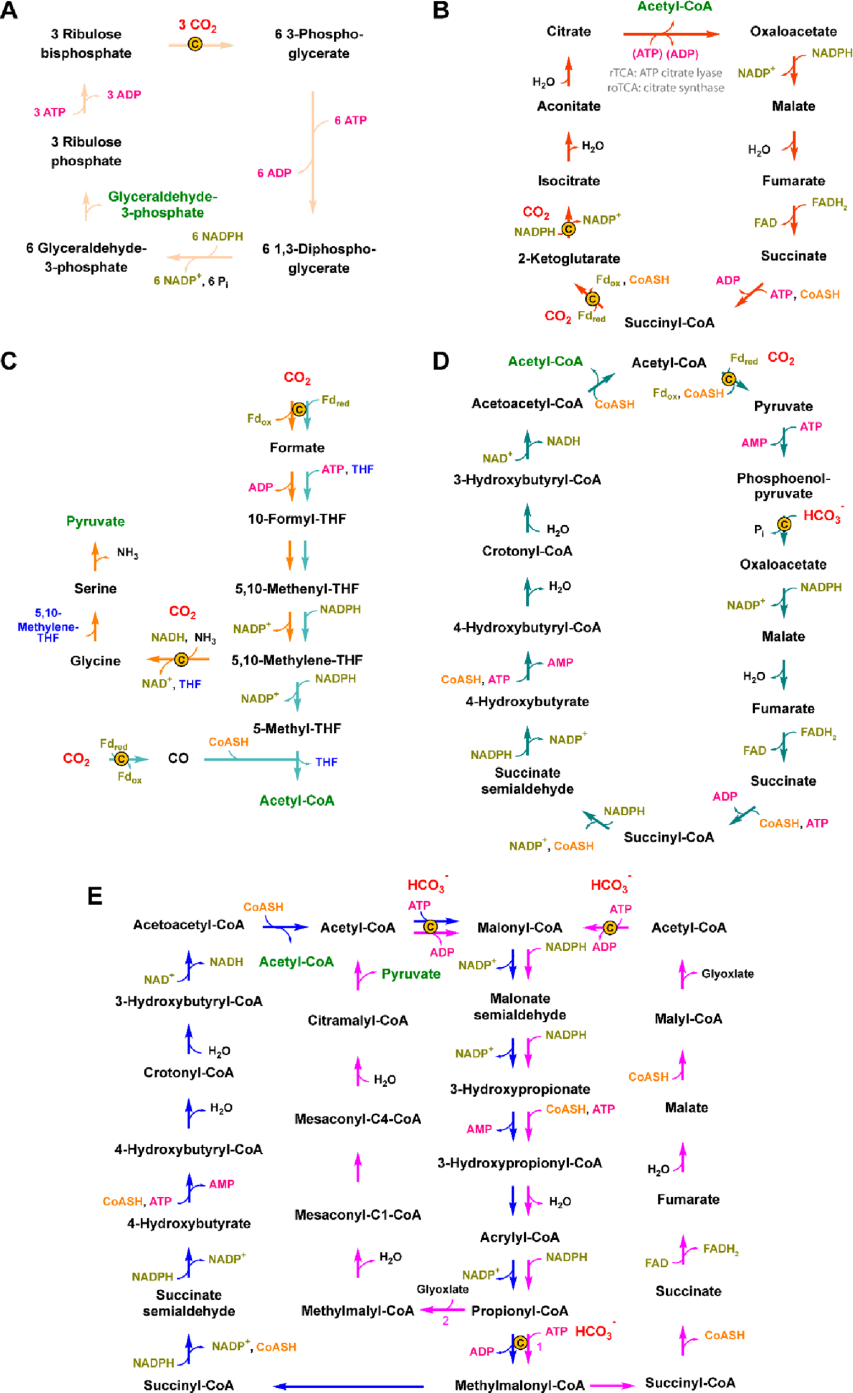
Natural carbon fixation cycles. (A) CBB cycle, (B) rTCA and roTCA cycle, (C) acetogenic WL pathway (turquoise) and reductive glycine pathway. (D) dicarboxylate cycle (orange). (E) 3HP/4HB cycle (blue) and 3HP bicycle (magenta). Carboxylation steps are highlighted.

The CBB cycle (Figure 14A) is nature’s predominant carbon fixation pathway. It occurs in plants, cyanobacteria, algae, and other photosynthetic bacteria.^86^ Almost all organic matter existing today was once fixed by the CBB cycle. The central carbon-fixing enzyme in the CBB cycle is RuBisCO (see section 3.1.1.1) that catalyzes carboxylation of RuBP to form two molecules of 3PG. Via 1,3-bisphosphoglycerate, glyceraldehyde-3-phosphate and a series of transaldolase reactions, 3PG is converted into ribulose-5-phosphate which is phosphorylated by phosphoribulokinase, forming RuBP and completing the cycle. Overall, three full turns of the cycle produce one molecule of glyceraldehyde-3-phosphate.

#### 4.1.2 rTCA Cycle

The rTCA cycle (Figure 14B and Table 5) was first discovered in green sulfur bacteria and is also present in eubacteria.^256^ It is essentially the reverse reaction of the TCA cycle and hence produces acetyl-CoA as final product. The two CO_2_-fixing steps are catalyzed by the enzymes 2-ketogluterate synthase and isocitrate dehydrogenase (cf. section 3.1.2). It has long been thought that citrate synthesis from oxaloacetate and acetyl-CoA, the key step of the oxidative TCA cycle (oTCA), is irreversible. Therefore, the reverse reaction present in the rTCA is either catalyzed by the reversible ATP-dependent citrate lyase (ACL)^295^ or the step is separated into two steps catalyzed by citrylyl-CoA synthetase and citrylyl-CoA lyase.^296,297^ In 2018, two pioneering studies showed that citrate synthases can also operate in the reverse direction, thereby constituting the reverse oxidative TCA cycle (roTCA).^281,290^ Although it has long been put forward that the rTCA cycle is strictly anaerobic, it can operate under microaerobic conditions.^298^

#### 4.1.3 WL and Reductive Glycine Pathway

Both the WL (Figure 14C, turquoise; Table 5) and the reductive glycine pathway (Figure 14D, orange) are linear pathways, which distinguishes them from all other CO_2_-assimilation pathways. Two different variants of the WL pathway are known: the acetogenic^291^ and the methanogenic.^67^ Both pathways share the same intermediates, with the only difference being that methanogens use methanofuran-based cofactors, while acetogens use tetrahydrofolate (THF) as cofactor. The WL pathway is strictly anaerobic as both CO_2_-fixing enzymes, CODH (see also section 3.1.2.5) and FDH/FMFDH (see also section 3.1.2.4), are highly sensitive to oxygen. The pathway fixes two molecules of CO_2_ and produces acetyl-CoA as final product, but it can also be used to metabolize other C1 compounds (see section 5).

The reductive glycine pathway (Table 5) was initially proposed as a hypothetical carbon fixation pathway for growth on formate.^117,119,299^ However, recently it was proposed that the phosphite oxidizing deltaproteobacteria *Candidatus Phosphitivorax anaerolimi* might use this pathway for carbon assimilation.^284^ In 2020, the pathway was demonstrated to sustain autotrophic growth in the sulfate-reducing bacterium *Desulfovibrio desulfuricans*.^62^ Although the glycine cleavage system, which is the pathway’s key carbon-fixing enzyme, is insensitive to oxygen, for fully autotrophic growth on CO_2_, 5,10-methylene tetrahydrofolate (5,10 mTHF) is required. Production of 5,10 mTHF can be achieved using formate as starting material, which in turn can be produced by the oxygen-sensitive enzyme FDH. Therefore, aerobic autotrophic growth on CO_2_ using the reductive glycine pathway is not possible.

#### 4.1.4 Dicarboxylate, 3HP/4HB Cycle, and 3HP Bicycle

The dicarboxylate cycle (Figure 14D and Table 5) is found in anaerobic archaea and, like the rTCA cycle, it includes oxygen-sensitive enzymes but tolerates microaerobic conditions.^300^ It fixes two molecules of CO_2_ via PEPC and PFOR (see sections 3.1.1.2 and 3.1.2.1) and yields acetyl-CoA as final product.

The 3HP/4HB cycle (Figure 14E, blue; Table 5) and the 3HP bicycle (Figure 14E, magenta; Table 5) occur in aerobic *Sulfolobales* and green nonsulfur bacteria respectively,^256^ have common intermediates and share the same carboxylating enzyme, ACPCC (see also section 3.1.1.3), which catalyzes both carboxylation steps. Both pathways can operate under aerobic conditions. However, while the 3HP/4HB cycle fixes two molecules of CO_2_ per round and produces acetyl-CoA as output, the 3HP bicycle fixes three CO_2_ molecules and produces the C3 compound pyruvate instead. A defining feature of the 3HP bicycle is that it is composed of two cycles which share several common steps (synthesis of propionyl-CoA from acetyl-CoA). One branch of the cycle produces glyoxylate, which the other branch uses as a substrate. All three pathways start from acetyl-CoA and produce succinyl-CoA as an intermediate. The 3HP/4HB cycle and the 3HP pathway use the same enzymatic transformations to produce succinyl-CoA from acetyl-CoA, while the dicarboxylate cycle is distinct. However, transformation of succinyl-CoA to acetyl-CoA is highly similar in the dicarboxylate and the 3HP/4HB cycle, while the 3HP bicycle is clearly different.

### 4.2 Development and Current State of Synthetic CO_2_ Fixation Pathways

Moving beyond naturally occurring CO_2_ fixation cycles, synthetic biologists have recently shifted their attention to the design, realization, and implementation of synthetic CO_2_ fixation cycles and linear cascades.

#### 4.2.1 Motivation

Although nature has evolved several different pathways for the capture of CO_2_ (outlined above), it has only populated a very small fraction of the possible solution space.^301^ Notably, many of the existing solutions represent only a local optimum and are still limited by the inefficiencies of the respective pathways and their enzymes. By designing, realizing, and implementing de novo CO_2_ fixation cycles, scientists aim to harness the favorable properties of highly efficient carboxylases, while designing thermodynamically- and energy-efficient (i.e., low ATP investment) reaction networks that regenerate the CO_2_ acceptor. Such synthetic CO_2_ fixation cascades are designed with the goal of augmenting natural CO_2_ fixation pathways in vivo, completely replacing natural CO_2_ fixation pathways in vivo, or providing a platform for the synthesis of compounds from CO_2_ in vitro or in vivo. Additionally, these synthetic CO_2_ fixation pathways can find applications in synthetic biology for the creation of synthetic autotrophic cells. In the near future, the development of synthetic, self-regenerating CO_2_ fixation pathways can provide an efficient solution for the synthesis of tailor-made fine chemicals for which nature has not evolved dedicated biosynthetic pathways from renewable resources. Overall, synthetic CO_2_ fixation pathways hold promise to increase, optimize, and enable the conversion of the greenhouse gas CO_2_ into valuable chemical compounds.

#### 4.2.2 Design

**Figure 15:**
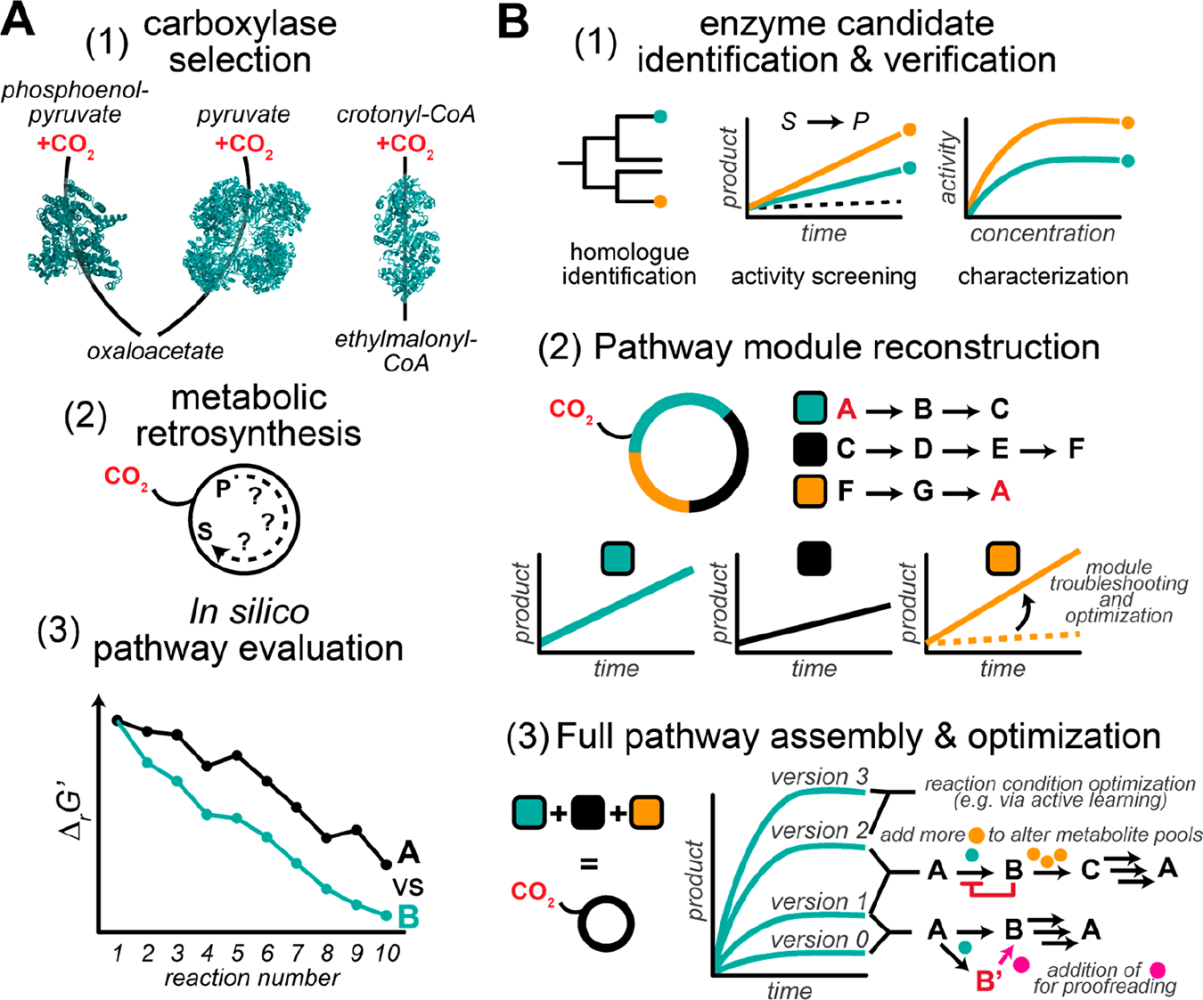
Design and realization of synthetic CO_2_ fixation pathways. (A) The theoretical considerations for the creation of new-to-nature pathways are (1) selection of a suitable carboxylase, (2) design of a pathway around the chosen carboxylase, and (3) pathway evaluation. (B) The experimental workflow for the assessment and optimization of new-to-nature (carboxylation) pathways entails (1) characterization of single enzymes, (2) reconstruction of pathway modules, and (3) full in vitro pathway assembly and optimization.

Synthetic CO_2_ fixation cycles or CO_2_-utilizing cascades are generally designed around carboxylases that exhibit desired properties, such as high rates of carboxylation or high energy-efficiency. Prospective carboxylases are sourced from nature and often are members of the PEPC, pyruvate carboxylase, or ECR enzyme families (Figure 15A(1)).^302,302^ Carboxylases of these families exhibit catalytic efficiencies that are orders of magnitude higher than those of the average enzyme, as well as those of carboxylases found in natural CO_2_ fixation cycles, such as RuBisCO.^81,302,303^ With the chosen carboxylase at hand, synthetic pathways are designed in a “metabolic retrosynthesis” phase,^137^ the aim of which is to identify a set of chemical reactions that efficiently converts the product(s) of the CO_2_-fixing reaction back to the substrate, while producing an organic output molecule that can be further metabolized (Figure 15A(2)). The efficiency and feasibility of drafted reaction cascades is then quantified based on a combination of (i) reaction cascade kinetics, (ii) energetic efficiency, (iii) thermodynamic feasibility, and (iv) difficulty of implementation (Figure 15A(3)).^137,255,294,299^

Reaction cascade kinetics (i) are most often evaluated using the rate-limiting step concept,^304^ where pathways are designed to contain fast rate-limiting steps, ensuring high flux. This is combined with a pathway specific activity metric, which describes the maximum theoretical rate of product formation by a given total protein mass of a pathway and thus quantifies the efficiency of all employed enzymes.^299^ In addition, recent studies have implemented Max–Min Driving Force (MDF) calculations to determine pathways that require low enzyme loadings to catalyze a unit of flux.^294,305−307^ While MDF evaluations contain no kinetic information, they can help to predict efficient pathways based on their thermodynamic driving force when comprehensive kinetic data is not available for all enzymes involved.^305^

Pathway energetic efficiencies (ii) are determined by how many reducing (e.g., NAD(P)H, FADH_2_, ferredoxins) and energy carrier (e.g., NTPs, CoA esters, phosphate-esters) equivalents are consumed per assimilated CO_2_ under physiologically relevant conditions (pH and ionic strength).^299^ Standardized energetic efficiencies are often directly compared between drafted and natural CO_2_ fixation cycles and thus help select efficient reaction cascades.^137,253^

Thermodynamic efficiencies (iii) are closely intertwined with a pathway’s energetic efficiency (ATP/NADPH equivalent cost). Pathways are designed to be exergonic, free of high thermodynamic barriers, and composed of reactions with high thermodynamic driving force.^305,308^ Thermodynamic feasibilities are evaluated by either calculating a pathway’s (or a pathway module’s) Gibbs free energy profile (Δ*rG*′) by, e.g., using eQuilibrator^43,42^ or by MDF analysis.^294,305^

Lastly, implementation feasibilities (iv) are a key determinant of pathway selections. This metric aims to quantify practical considerations such as the number of required enzymes, their oxygen-tolerance, availability of catalysts for all desired reactions, compatibility with host–organism metabolism, and the specificity of the utilized catalysts.

Because these evaluation criteria are applied to highly diverse pathways with strongly varying implementation goals, no single quantitative metric can be derived to parametrize evaluation outcomes. Efficiency and feasibility thus have to be evaluated on a pathway-to-pathway basis. This is nicely highlighted by the PYC-OAH-ACS-PFOR (POAP) cycle (Table 5),^253^ which assimilates CO_2_ in a four-enzyme cycle with the oxygen-sensitive and thermophilic PFOR (see section 3) at its core. The oxygen-sensitivity and high temperature optimum of PFOR immediately render this pathway unsuited for implementation into mesophilic, aerobic organisms, yet exactly these qualities, as well as its short nature, make it perfectly suited for an implementation as a(n) (auxiliary) CO_2_ fixation pathway in anaerobic thermophiles.

Despite the flexible nature of the evaluation criteria, an end-goal specific evaluation step of the drafted CO_2_ fixation cycle is highly important, which is nicely highlighted in the creation of the crotonyl-Coenzyme A (CoA)/ethylmalonyl-CoA/hydroxybutyryl-CoA (CETCH) cycle (Table 5).^137^ Before realizing the CETCH cycle, Schwander et al. evaluated a total of seven drafted synthetic CO_2_ fixation cycles built around the carboxylase crotonyl-CoA carboxylase/reductase. Not only was the CETCH cycle the most exergonic pathway among drafts with similar energetic efficiencies, but it also represented the shortest pathway (11 enzymes required) and enzyme candidates were available for all desired reactions. This in-depth evaluation during the initial pathway design phase was crucial for the successful in vitro realization of the first completely synthetic CO_2_ fixation cycle.

Beyond cyclic CO_2_ fixation pathways, recent efforts also focused on the design of linear CO_2_ fixation cascades to either augment natural metabolism^55^ or to enable the synthesis of products directly from CO_2_.^255^ The recently described three-enzyme tartronyl-CoA (TaCo) pathway,^55^ for example, converts glycolate into glycerate, while assimilating one molecule of CO_2_ in the process. In doing so, the TaCo pathway enables a carbon-positive conversion of 2PG, the product of photorespiration, into an intermediate of central metabolism. This enables the replacement of the naturally carbon-releasing photorespiration^309^ by a synthetic, carbon-positive alternative that augments natural metabolism. The TaCo pathway was designed to be short and efficient, yet it included a carboxylation step that is not catalyzed by any known enzyme (namely the carboxylation of glycolyl-CoA to (*S*)-tartronyl-CoA). However, chemically similar reactions, such as the carboxylation of propionyl-CoA to methylmalonyl-CoA, are catalyzed by naturally occurring biotin-dependent carboxylases, which is why Scheffen et al. deemed the re-engineering of a naturally occurring carboxylase into a glycolyl-CoA carboxylase possible.

Lastly, synthetic CO_2_ fixation cascades have recently been combined with chemical catalysts which reduce CO_2_ prior to further chemoenzymatic conversion.^255,310^ These hybrid systems increase the chemical and enzymatic reaction space available during pathway design phases, as enzymes for the conversion of reduced C1 species can be used (see section 5). Furthermore, initiating chemoenzymatic cascades using formate or methanol as starting material, increases the theoretical energetic efficiency of CO_2_-to-product conversions, due to the high energy efficiency of chemical CO_2_ reductions.^311,312^ Reaction cascades based on reduced C1 species will be reviewed in section 5, yet the design, evaluation, and realization principles closely resemble those used to create purely chemoenzymatic CO_2_ fixation cascades.

#### 4.2.3 Realization and Implementation

After designing a reaction cascade, enzyme activities for individual reactions or selected pathway modules are validated in vitro prior to full cascade assembly. This stepwise reconstitution and validation serves to break down the complex reaction cascade into smaller sections, which are easier to validate and troubleshoot. Synthetic CO_2_ fixation cascades are generally first realized in controlled in vitro environments, prior to in vivo implementation attempts, because reaction conditions can be tightly defined, controlled, and adapted in in vitro systems.

This stepwise in vitro realization principle is nicely highlighted in the establishment of the reductive glyoxylate and pyruvate synthesis and malyl-CoA-glycerate (rGPS-MCG) cycle (Table 5).^294^ The rGPS-MCG cycle employs the highly efficient PEPC and CCR to synthesize acetyl-CoA from two CO_2_ molecules while regenerating the carboxylase substrates in a multistep cascade. During its realization, Luo et al. first validated the activity of each enzyme individually or sequentially (depending on the availability of read-out modules). Thereafter, the cycle was split into three sections, each of which was assembled separately. Thereby, the feasibility the rGPS-MCG cycle could be demonstrated. Subsequently, the rGPS-MCG cycle was fully assembled in vitro and equipped with sensing modules for online monitoring of concentrations of NAD(P)H, ATP, and FAD, as well as other reductants. This extensive sensing setup revealed inefficiencies in enzyme homologue choice, side reactivities, enzyme instabilities, and cofactor balance problems. After correcting for the identified limitations, the rGPS-MCG cycle was successfully realized in vitro and reached continuous quasi-steady state operation for up to 6 h (Figure 15B).

Similar inefficiencies were identified by continuously monitoring the ^13^CO_2_ label incorporation during the realization of the CETCH cycle.^137^ Despite initial validation of enzyme activities in a “modularized” fashion, product synthesis in the fully assembled cycle halted prematurely. Eventually, methylsuccinyl-CoA dehydrogenase (mcd) was pinpointed as problematic as it was operated using the artificial electron acceptor ferrocenium. High concentrations of ferrocenium prevented effective cycling of CETCH, which is why Schwander et al. re-engineered mcd to accept O_2_ as an alternative, noninterfering, electron acceptor.^137,313^ The same issue was encountered during the design of the rGPS-MCG cycle (described above) and circumvented by carefully fine-tuning the employed concentrations of ferrocenium.^294^ The stepwise establishment of the CETCH cycle further highlighted the need for fine-tuned reaction conditions, cofactor regeneration systems, and most importantly proof-reading enzymes that facilitate a re-entry of dead-end metabolites into the cycle. Such proof-reading reactions were used to prevent, e.g., the unwanted build-up of malyl-CoA in the CETCH cycle,^137^ or to minimize the build-up of methylsuccinate in the rGPS-MCG cycle.^294^

While cyclic pathways are especially susceptible to premature arrest due to intermediate drainage, the recent realization of the artificial starch anabolic pathway (ASAP), a linear pathway for the synthesis of starch from CO_2_, also required extensive fine-tuning efforts in order to balance enzyme reactivities and prevent inhibitory effects exerted by pathway intermediates.^255^ In ASAP, CO_2_ is chemically reduced to formaldehyde and subsequently converted to starch in a chemoenzymatic reaction cascade. Enzymes of ASAP that were allosterically inhibited by cycle intermediates had to be re-engineered to be less susceptible to inhibition and enzymes exhibiting high cofactor competition had to be engineered to be more efficient, in order to successfully realize ASAP in vitro.

Although the highlighted monitoring and optimization efforts were successful in creating in vitro CO_2_ fixation cascades, the final pathway efficiency is often still far from optimal. This is largely because the individual parts used to reconstruct these systems are derived from drastically different biological backgrounds, which makes their interactions hard to predict. Recent efforts have therefore implemented a machine learning-guided workflow called METIS^254^ to explore the vast combinatorial space of reaction conditions. By screening the efficiency of only ∼1000 different pathway variants over a total of eight rounds of active learning, the productivity of the CETCH cycle could be improved roughly 10-fold compared to the previously best pathway combination.^137,254^ In theory, workflows like METIS can be used from the get-go to identify and optimize reaction conditions for efficient realization of CO_2_ fixation cascades. These reaction condition optimizations can be paired with a novel computational tool (MEMO) developed to identify the smallest possible metabolic modules with a defined stoichiometry.^307^ MEMO can, e.g., predict short metabolic modules to regenerate the 4 NADPH, 1 ATP, and 1 acetyl-CoA consumed during operation of the CETCH cycle and thus predicts cofactor regeneration as well as carbon assimilation modules that can be tested in the realization phase. Such computational workflows have already proven to be highly useful for in vitro optimization of pathways. These in vitro realizations and optimizations are crucial steps for downstream in vivo pathway implementation or for the use as in vitro production platforms.^255,310,314,315^

Recently, Erb et al. defined five levels of metabolic engineering. Levels 1 and 2 encompass the optimization of natural pathways and the transplantation of naturally occurring pathways, whereas levels 3, 4, and 5 encompass the creation of new-to-nature pathways by recombining enzymes that catalyze their native reaction, novel reactions based on known mechanisms, or novel reactions based on novel enzymatic mechanisms, respectively.^308^ The CO_2_ fixation cascades highlighted in section 4.2.2 and 4.2.3 all represent level 3 or 4 pathways, as they are (per current knowledge) truly new-to-nature. No new-to-nature pathway (level 3 or higher) for the fixation of CO_2_, has successfully been implemented into host organisms yet. However, computational research predicts that successful implementations carry the potential to improve both natural CO_2_ fixation^137,316^ and photorespiration.^317^ Although not representing a full in vivo implementation, first steps toward integrating the CETCH cycle into complex biological systems were realized by linking the artificial CO_2_ fixation cascade to the natural photosynthetic machinery, capable of replenishing energy- and reducing-equivalents from light.^278^ These artificial chloroplast mimics are functionally equivalent to their natural counterparts and even exceed the latter in CO_2_ fixation efficiency.

To harness their full potential for biotechnology and agriculture, synthetic CO_2_ fixation cycles will need to be successfully implemented into living systems. This implementation currently poses several technical and biological challenges. First, it requires methods to encode and assemble the necessary genetic information in a compact and tunable fashion. Second, these synthetic pathways need to be integrated into the native genetic and metabolic background of the host. To this end, the current strategy of choice is the utilization of selection strains that are designed to require the output molecule of a given CO_2_ fixation cascade for growth.^70^ Such selection strains can be fine-tuned to derive different percentages of their total biomass from the output molecule that is being selected for, which results in varying degrees of selection pressure for the non-natural pathway.

While not successfully applied for synthetic CO_2_ fixation cycles yet, selection strains have been vital for the improvement and implementation of several level 1 and 2 pathways focused on establishing the ability to assimilate CO_2_ or other C1 compounds in model organisms.

Satanowski et al., for example, improved a latent carboxylation cycle, the Gnd–Entner–Doudoroff (GED) cycle, via laboratory evolution of *E. coli* selection strains (Table 5).^70^ The GED cycle is composed solely of enzymes native to the heterotrophic *E. coli*, which form a pathway that was able to supply selection strains with CO_2_-derived biomass after short-term laboratory evolution. This *E. coli*-native CO_2_ fixation pathway shows similar pathway specific activity to the CBB cycle and has the potential to improve CO_2_ fixation if better enzyme homologues for the key reaction are engineered or identified in nature.^316^

Similarly, recent efforts saw the introduction of a functional CBB cycle into *E. coli* strains that derived either all sugars^318^ or all biomass from CO_2_.^319,320^ Autotrophic growth was achieved after long-term evolution under selection for CO_2_ fixation via the CBB cycle. These efforts nicely highlight that even transplantation of natural CO_2_ fixation cycles into heterologous hosts is associated with difficulties. Many mutations, most of which were difficult to predict, were required to rebalance intracellular fluxes and enable autotrophic growth. Equally complex rewiring efforts are expected to be required for the successful implementation of truly new-to-nature CO_2_ fixation cascades.

Recently, the first “level 3” pathway, a new-to-nature pathway composed of enzymes catalyzing their native reactions, was realized in acetogenic bacteria.^321^ This pathway, the so-called acetyl-CoA bicycle, fixes carbon via PFOR,^58^ converts the produced pyruvate into hexose phosphates via gluconeogenesis and subsequently converts the hexose phosphate into the acceptor compound acetyl-CoA, while producing a surplus acetyl-CoA, via nonoxidative glycolysis. Implementation of the acetyl-CoA bicycle made use of 15 native enzyme activities of the clostridial host bacterium and required the introduction of only a single heterologous phosphoketolase to initiate the nonoxidative glycolysis part of the pathway.

Lastly, multiple natural C1 fixation pathways were recently transplanted into heterologous hosts and shown to be responsible for the assimilation of most to all biomass in specialized selection strains. These efforts include the successful introduction of the ribulose monophosphate shunt into *E. coli* strains that end up deriving either all^322^ or most^323,324^ of their biomass from methanol or formaldehyde, respectively. Similarly, growth of *E. coli* on formate and methanol as the sole carbon sources was recently established via a similar strategy that involved short-term laboratory evolution of tailor-made selection strains carrying the reductive glycine pathway.^113^ Importantly, these successful pathway examples all have reduced C1 compounds as substrates, which can also be utilized as an energy source. This arguably facilitates their implementation into heterologous hosts, as the need for an external energy source is alleviated.

Together, these successful implementations of level 1 and 2 C1-assimilation pathways highlight the challenges, requirements, and strategies associated with introduction of truly new-to-nature CO_2_ fixation cascades into living organisms.

### 4.3 Biocatalytic Cascades Using CO_2_ as C1 Building Block for Fine Chemicals

Natural and synthetic CO_2_ fixation systems usually produce compounds from central carbon metabolism, as those can in turn be used to biosynthesize all other biomolecules required by the cell. However, for purely synthetic applications, other target molecules are more attractive. To this end, decarboxylases represent an attractive enzyme class for application in biocatalytic cascades to produce synthetically relevant target molecules.^232^ Although several examples of cascade reactions involving a decarboxylation step exist, only a limited number of CO_2_ utilization cascades are reported.^10^ However, such processes hold great potential, as they allow the application of CO_2_ as C1 building block in synthesis. Furthermore, as outlined in sections 2 and 4.2, the combination of carboxylases and decarboxylases with other enzymes promises to overcome the thermodynamic limitations of the challenging carboxylation reaction.

An efficient method to drive the carboxylation reaction of prFMN dependent decarboxylases and to modify the formed carboxylic acids is combining it with carboxylic acid reductases (CAR) to transform the carboxylic acid to an aldehyde (Table 6, entry 1).^211^ The aldehyde moiety opens many possibilities for further functionalization and valorization. For example, *A. niger* decarboxylase was coupled to CARs and the alcohol dehydrogenase (ADH) from *E. coli* to produce cinnamyl alcohol (Table 6, entry 2). Using the same prFMN-CAR system in the presence of amines and an imine reductase allowed production of secondary amines, while employing a reductive aminase allowed production of amides (Table 6, entries 3 and 4). By replacing Ile327 with Ser, the substrate scope of *An*Fdc was extended for carboxylation of the heteroaromatic substrate benzofuran. This allowed production of various benzofuran derivatives (Table 6, entries 5–7).^211^ The heteroaromatic substrate scope was further expanded by application of the indole-3-carboxylic acid decarboxylase from *Arthrobacter nicotianae* for the carboxylation of indole and coupling it to CAR (Table 6, entry 8). The aldehyde product is a valuable building block for the synthesis of anticancer active pharmaceutical ingredients (APIs) like indole phytoalexins brassinin or 1-methoxyspirobrassinol methyl ether.^189^ Furthermore, the CAR from *Tsukamurella paurometabola* was exploited for NADPH-free amidation, subsequent to prFMN-dependent styrene carboxylation to yield cinnamamide.^211^

Reverse decarboxylation, i.e., carboxylation was applied in the production of the amino acids l-methionine, l-leucine, and l-isoleucine by reversing the Ehrlich pathway (see also section 3.2.4).^39^ Methional was carboxylated by KdcA, yielding an α-keto acid, which is then converted to l-methionine or l-leucine using a methionine aminotransferase or leucine dehydrogenase, respectively (Table 6, entries 9 and 10). The production of l-methionine by applying YbdL is quite costly, as it requires L-Gln as amino donor. The utilization of LeuDH is therefore advantageous, as it accepts inorganic ammonia and consumes NADH, which can easily be recycled via established methods. Applying 2-methylbutanal as a substrate results in l-isoleucine after transformation by the cascade.^39^

In another example, ethanol was converted to l-lactic acid using a three-enzyme catalytic system involving an ADH for alcohol oxidation, a pyruvate decarboxylase for the carboxylation of acetaldehyde and a lactate dehydrogenase to reduce the pyruvate to l-lactate (Table 6, entry 12). This multienzymatic system involves an internal cofactor regeneration system known as borrowing hydrogen, as the NAD^+^ that is consumed by the ADH is recycled within the cascade by the LDH, which makes the system overall redox neutral.^149^ Note, that pyruvate is a core metabolite of central carbon metabolism. Therefore, this system might be further utilized in (artificial) metabolic pathways (compare section 4.2)

## 5 Approaches to Use CO_2_ Derivatives

### 5.1 Benefits to a Bioeconomy Based on Soluble One-Carbon Compounds

As discussed above, CO_2_ is the most oxidized C1 species available in nature. Biological carbon assimilation requires its reduction to hydrocarbons, consuming energy. A comprehensive study of the conversion of C1 substrates showed that more reduced C1 compounds are assimilated at higher energetic efficiency than CO_2_, both aerobically and anaerobically (Table 7).^325^ In particular, the water-soluble C1 species formate, formaldehyde, and methanol are promising C1 compounds for a circular bioeconomy as potential mass transfer barriers are eliminated. While these molecules can be produced enzymatically by the reduction of CO_2_,^326−329^ this requires either expensive redox equivalents, usually ferredoxins and/or NAD(P)H^326−328^ or molecular hydrogen.^125,126^ Alternatively, electrochemical hydrogenation can be used for the direct production of these compounds with off-peak renewable energy as reductive power. While this approach requires high CO_2_ concentration and can suffer from low efficiency and selectivity toward the desired product, it provides carbon-neutral energy and hydrogen storage^330,331^ and allows biotechnological generation of more complex products from reduced C1 compounds. Although soluble C1 compounds are easy to store and can be made available to platform organisms at high titers, they also pose challenges: some of them are toxic to both the cultured organisms as well as humans, and they can pose fire hazards if stored in bulk. Additionally, only a limited number of organisms can naturally metabolize them, most of which are not genetically tractable or difficult to cultivate. Therefore, new strategies are developed that include on the one hand the realization of in vitro cascades, where substrate toxicity is less limiting, and on the other hand the engineering of common platform organisms toward growth on C1 compounds. Note that the assimilation of reduced C1 compounds is less dependent on carboxylases, but mainly relies on carboligases that show distinct cofactor requirements and mechanisms.

**Table 7:** Overview of Natural and Synthetic C1 Fixation Pathways^a^

pathway	starting material	primary product	ATP	NAD(P)H	ATP eq (acetyl-CoA)	status	ref
serine	FALD	acetyl-CoA	3	2	8.00	natural	(334)
mod. serine	FALD	acetyl-CoA	4	2	9.00	synthetic (in vivo)	(337)
homoserine	FALD	acetyl-CoA	1	0	1.00	synthetic (in vivo)	(338)
RuMP	FOR	GA3P	3	0	–4.00	natural	(332)
XuMP	MeOH	DHA	0	0	–6.00	natural	(333)
FORCE	MeOH	glycolate	0	0	0	synthetic (in vivo)	(339)
HWLS	FOR	DHAP	4	3	4.50	synthetic (in vivo)	(340)
ASAP^b^	MeOH	starch	2	0		synthetic (cell-free)	(255)
SACA	FALD	acetyl-CoA	0	0	0.00	synthetic (in vivo)	(341)
SMGF^c^	FOR	pyruvate	2	2	4.50	synthetic (in vivo)	(342)
lin Met	MeOH	DHAP	1	–3	–13.50	synthetic (in vivo)	(343)

a RuMP, ribulose monophosphate pathway; XuMP, xylulose monophosphate pathway; FORCE, formyl-CoA elongation pathway; HWLS, Half–Wood–Ljungdahl–Formolase pathway; ASAP, artificial starch anabolic pathway; SACA, synthetic acetyl-CoA pathway; SMGF, synergistic metabolism of glucose and formate pathway; lin Met, linear methanol assimilation pathway; DHA, dihydroxyacetone; DHAP, dihydroxyacetone phosphate; FOR, formate; FALD, formaldehyde; MeOH, methanol;

b Normalized against incorporation of one C6 sugar to starch;

c Only C1 branch;

### 5.2 The Diversity of Natural C1 Assimilation

Natural formate and methanol assimilation pathways demonstrate a surprising amount of variety. Six natural pathways have been described so far: the ribulose monophosphate (RuMP) pathway,^332^ xylulose monophosphate (XuMP) pathway,^333^ the serine cycle,^334^ the reductive glycine pathway,^119^ the WL pathway,^335^ and the CBB cycle (compare also Table 7).^336^ The latter three pathways are also CO_2_-fixing and were already discussed in sections 4.1.3 and 4.1.1.^119,335^ The reductive glycine pathway in particular has been implemented into different platform organisms, which was summarized in a recent review.^292^ In each pathway, the carbon assimilation steps are distinct from each other with respect to their mechanisms and cofactor requirements. In the following, we will discuss these natural pathways and mechanisms and the synthetic solutions in context.

### 5.3 Tetrahydrofolate (THF) Cascade

**Scheme 24:**
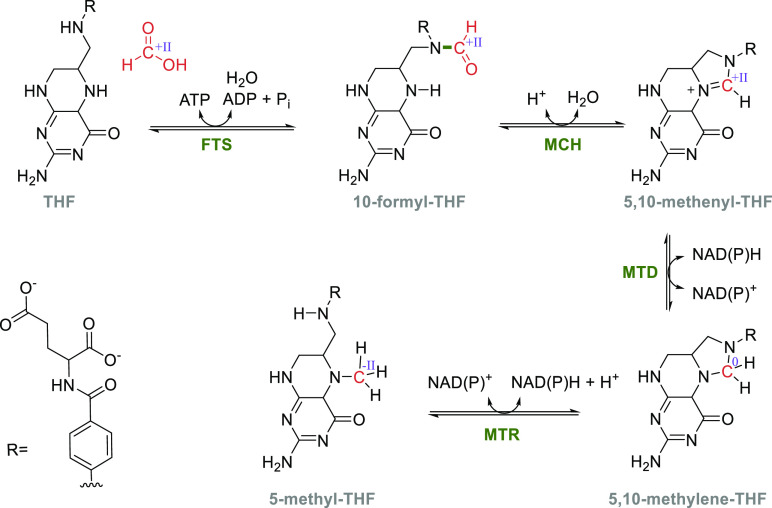
Reductive THF Cascade The carbon of formate is reduced in a stepwise fashion from oxidative state +II to −II. The oxidative state of the relevant carbon atom is indicated in roman numerals. FTS = formyl-THF synthase, MCH = methenyl-THF cyclohydrolase, MTD = methylene-THF dehydrogenase, MTR = methylene-THF reductase.

A common feature of methanol, formate, and formaldehyde assimilation is that these C1 compounds often enter the central carbon metabolism via THF- (bacteria) or H_4_MPT- (archaea)bound intermediates .^344^ As they fulfill a similar function, we will only discuss THF here. THF serves as a chemical handle to activate the C1 compound, to facilitate binding to enzymes, and to reduce substrate toxicity. The full cascade shown in Scheme 24 starts by reaction of formate with THF to formyl-THF, followed by dehydration to the circularized methenyl-THF, reduction to methylene-THF, and finally reduction to methyl-THF. Incorporation into pathways usually occurs via methylene- or methyl-THF. Notably, formaldehyde is able to spontaneously react with THF to form methylene-THF, permitting an entry into the cascade.^345^ Introduction of the partial or full THF cascade has been used to enable synthetic formate assimilation in platform organisms.^340,346^

### 5.4 WL Pathway and Metal Cofactors

**Scheme 25:**
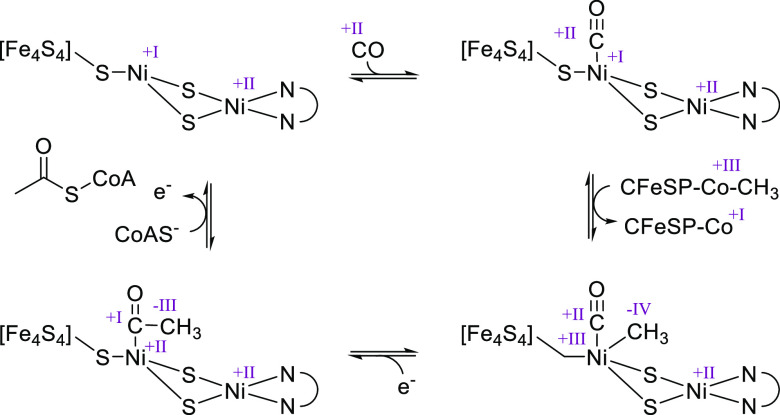
Reaction Mechanism at the NiFeS Cluster of ACS Note that the shown mechanism represents only one possibility. Alternative mechanisms involving Ni(0) species have also been proposed.^26^.

The WLpathway is found in archaea and is one of the most versatile carbon assimilation pathways, able to assimilate multiple C1 sources, including CO_2_, CO, and formate.^291,347^ Reduction of CO_2_ by FDH and CODH have been discussed in previous sections (see sections 3.1.2.4 and 3.1.2.5). Here, we will focus on the pathway’s core enzyme acetyl-CoA synthase (ACS) (Scheme 25).^26^

The WL pathway is split into a methyl and a carbonyl branch. ACS is the enzyme linking the two branches. CO generated in the carbonyl-branch can bind to the NiFeS-cluster of ACS. Within the methyl branch of the WL pathway, formate is reduced to methyl-THF via the THF cascade (Scheme 24). This methyl group is transferred to the cobalt cofactor of corrinoid iron–sulfur protein (CFeSP). ACS can accept the methyl group from CFeSP and transfer it to its nickel–iron–sulfur (NiFeS) cluster (Scheme 25). Acetyl-CoA is synthesized at the NiFeS cluster by linking the methyl group to the CO. Finally, acetyl-CoA is released from the cofactor by the transfer of the acetyl group to CoA and the cofactor is regenerated. Due to the specific cofactor requirements of its core enzymes (FDH, CODH, and ACS), transfer of the WL pathway to non-native hosts is expected to be rather challenging.

**Scheme 26:**
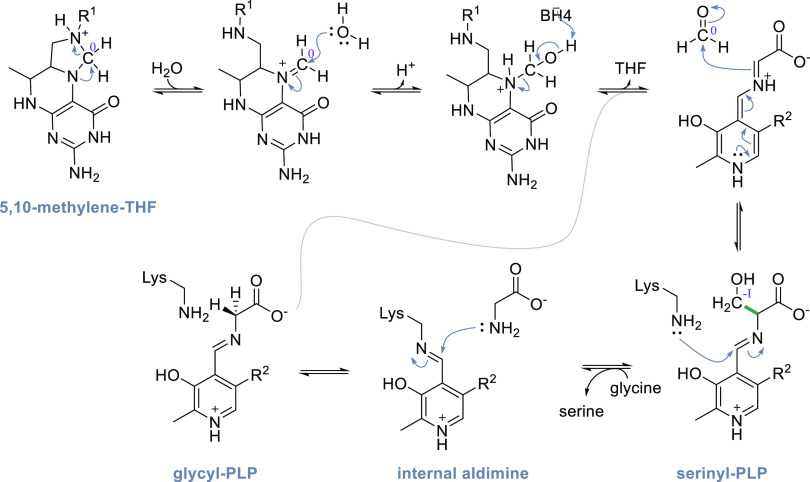
Reaction Mechanism of SHMT Mechanism for the hydrolysis of 5,10-methylene tetrahydrofolate to formaldehyde and THF followed by the aldol condensation of glycine with formaldehyde to serine.^348^ The oxidative state of the relevant carbon atoms is indicated in roman numerals.

**Scheme 27:**
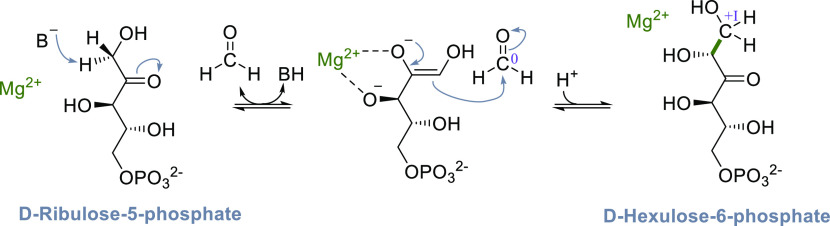
Reaction Mechanism of HPS Showing Ru5P Condensation with Formaldehyde;^354^ The Oxidation States of Relevant Carbon Atoms Are Given in Roman Numeral

### 5.5 Serine Cycle and Pyridoxal Phosphate Dependent Enzymes

**Figure 16:**
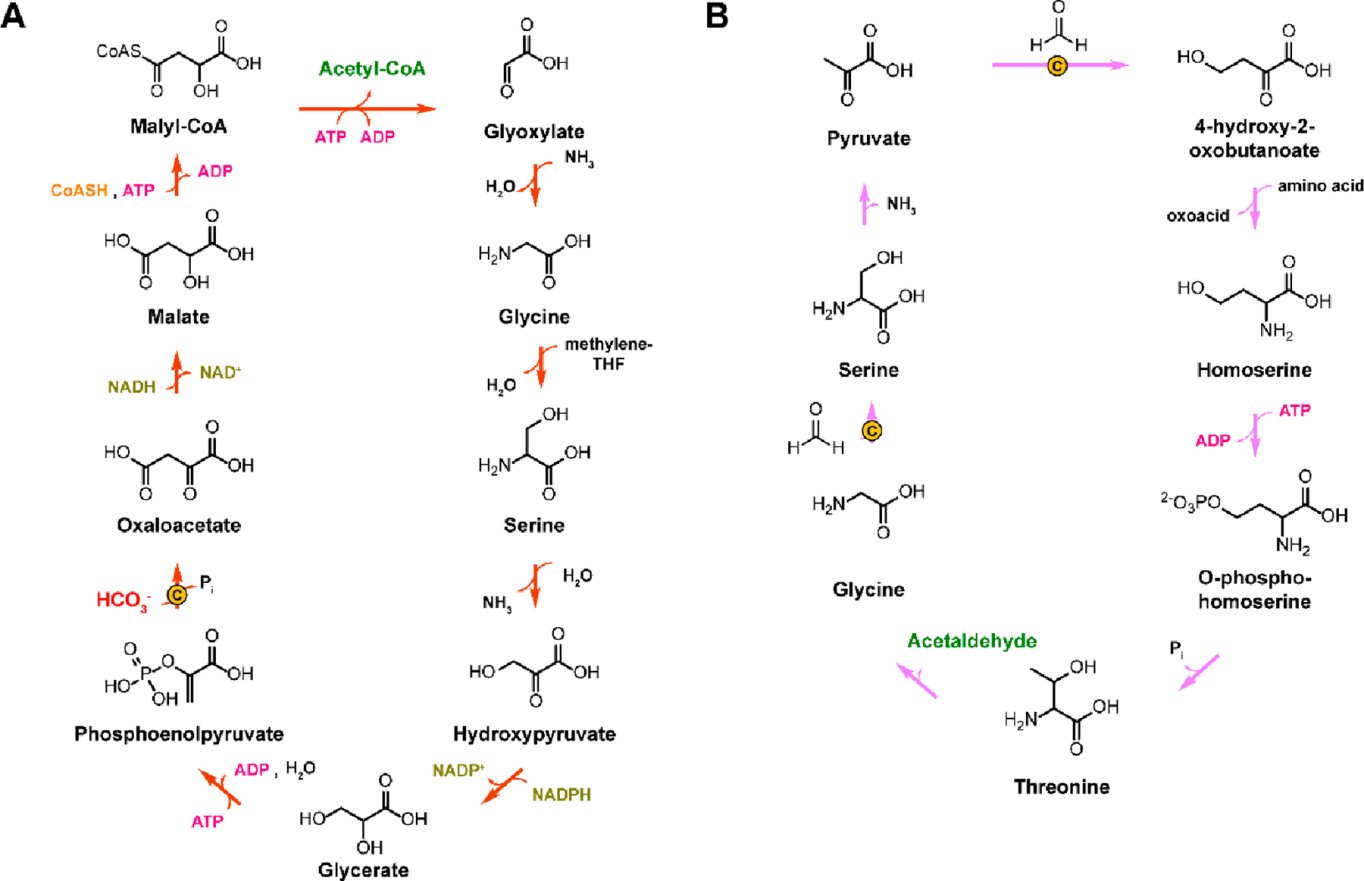
(A) Serine cycle and (B) homoserine cycle. Carboxylation/C_1_ elongation steps are highlighted.

The serine cycle naturally occurs in aerobic methylotrophic organisms, where it incorporates the two C1 units, methylene-THF (which can be derived from formate or formaldehyde) and bicarbonate to form acetyl-CoA (Figure 16). In the pathway, serine is produced from glycine and methylene-THF. Via a series of enzymatic steps, serine is converted to PEP, which is carboxylated by PEPC, thereby assimilating bicarbonate and forming oxaloacetate. Oxaloacetate is transformed to malyl-CoA by reduction and CoA ester formation. Then, malyl-CoA is cleaved, forming acetyl-CoA and glyoxylate, which is converted to glycine by serine glyoxylate aminotransferase.

**Figure 17:**
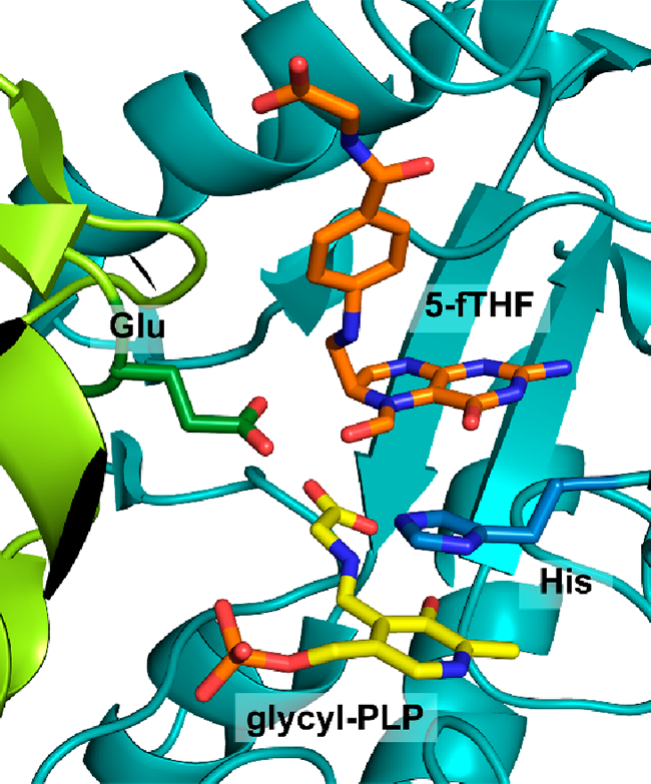
Crystal structure of *G. stearothermophilus* SHMT (PDB 1KL2(349)) with 5-formyl-tetrahyrofolate (5-fTHF; orange) and glycyl-PLP adduct (yellow). The two subunits that create the active site are indicated in different colors. The postulated general bases glutamate (green) and histidine (blue) are shown as sticks.

Serine hydroxymethyltransferase (SHMT) is the serine cycle’s key enzyme and catalyzes the PLP-dependent reaction of methylene-THF and glycine to serine (Scheme 26). While the exact mechanism and intermediates of the reaction are debated, recent studies suggest that the addition occurs as an aldol reaction with formaldehyde as proposed intermediate.^348−351^ As SHMT also accepts formaldehyde as substrate, the assumption that free formaldehyde is involved in the enzymatic mechanism seems plausible.^352^ Two computational studies investigated the reaction in the retro-aldol direction, giving insights into the enzyme mechanism.^348,351^ While both studies agree on the general mechanism, they find slightly different lowest-energy reaction paths and disagree on the identity of the general base, which has been suggested to be either a glutamate^351^ or a histidine^348^ (Figure 17). The SHMT mechanism can be separated into two half-reactions: first, the hydrolysis of methylene-THF to formaldehyde and THF, and second, the PLP-dependent aldol reaction of formaldehyde with glycine to form serine (Scheme 26). The glycyl-PLP species required for the reaction is generated via an enzyme-bound internal aldimine intermediate.

Individual steps of the serine cycle, including SHMT, have been introduced into *E. coli* to enable formate assimilation,^353^ and a modified version of the full cycle has been implemented in *E. coli* (Table 7).^337^ A more derived variant, the homoserine cycle (Figure 16B),^338^ utilizes PLP-dependent aldolases for both of its C1-assimilating steps, with both of them incorporating formaldehyde instead of methylene-THF. In the homoserine cycle, a promiscuous serine-threonine aldolase, replaces SHMT and catalyzes the C1 elongation of glycine to serine. In contrast to the serine cycle, serine is subsequently not converted to PEP but to pyruvate instead. Then, 4-hydroxy-2-butanoate aldolase catalyzes the C1 elongation of pyruvate to 4-hydroxy-2-butanoate. This metabolite is converted to threonine via homoserine and phosphohomoserine. In the last step, threonine is cleaved to from glycine and acetaldehyde, which can be converted to acetyl-CoA. Although both the modified serine cycle as well as the homoserine cycle are functional in vivo, neither is able to sustain growth of *E. coli* on formaldehyde (or methanol) as the sole carbon source thus far.^337,338^

### 5.6 RuMP Pathway and RuBisCO-like Reactions

**Figure 18:**
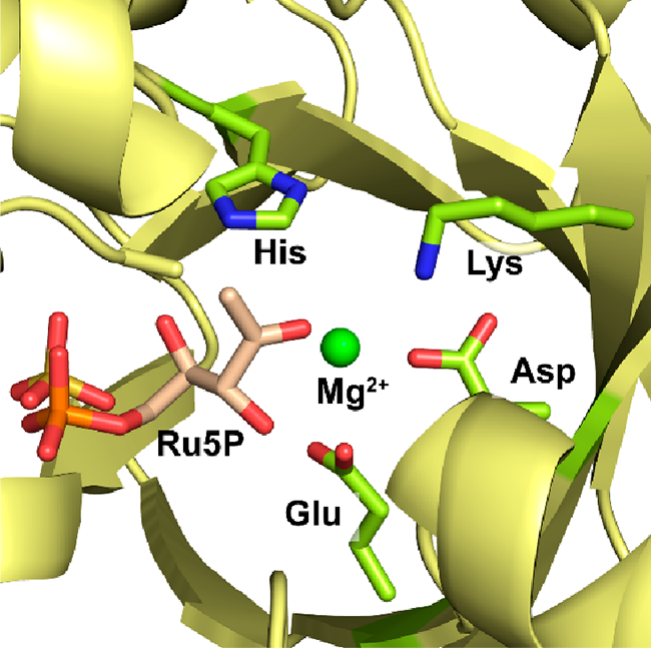
Crystal structure of *M. gastri* HPS (PDB 3AJX)^355^ with ribulose-5-phosphate (Ru5P) modeled in from *E. coli* 3-keto-l-gulonate-6-phosphate decarboxylase structure (PDB 1XBV).^354^ The Mg^2+^-coordinating charged residues, as well as the histidine base, are shown as sticks.

The RuMP pathway is an aerotolerant pathway present in methylotrophic bacteria (Table 7). Its core enzyme, 3-hexulose-6-phosphate synthase (HPS; Scheme 27), catalyzes the reaction of formaldehyde with RuMP to hexulose-6-phosphate. Hexulose-6-phosphate can be further converted to fructose-6-phosphate, thereby entering glycolysis. Similar to RuBisCO, HPS forms an enolate intermediate by abstraction of a proton and stabilizes it with a Mg^2+^ (Figure 18). The charged intermediate then performs a simple nucleophilic attack on formaldehyde, followed by protonation and release of 3-hexulose-6-phosphate.^354^

The transfer of the RuMP pathway into different host organisms has been extensively reviewed.^356^ Recently, the RuMP pathway was also combined with nonoxidative glycolysis to enable the biosynthesis of higher alcohols from methanol without requiring ATP,^357^ and the pathway was used to confer synthetic methylotrophy in *E. coli*.^322^

### 5.7 XuMP Pathway and Thiamine Pyrophosphate Dependent Reactions

**Scheme 28:**
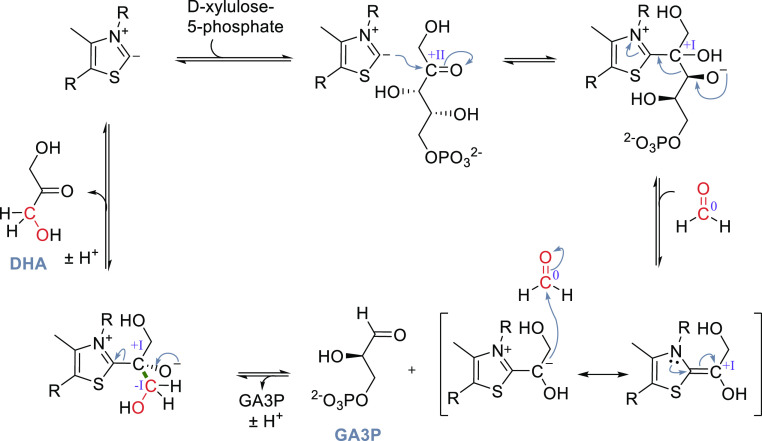
Postulated TPP-Dependent Mechanism of DAS; The Oxidation State of Relevant Carbon Atoms Are Given in Roman Numerals

The XuMP pathway is an aerotolerant pathway present in methylotrophic yeasts (Table 7). Its core enzyme is dihydroxyacetone synthase (DAS), a TPP-dependent enzyme converting xylulose-5-phosphate to glyceraledehyde 3-phosphate (GA3P) and dihydroxyacetone. While the mechanism of DAS has not been studied in detail, it is very likely that it uses a mechanism similar to that of other TPP-dependent lyases (Scheme 28).^358−360^ Deprotonated TPP performs a nucleophilic attack on the keto group of XuMP. Next, GA3P is eliminated, producing a reactive enolate/carbanion intermediate. The carbanion then acts as nucleophile, attacking formaldehyde, yielding an adduct which further reacts to form dihydroxyacetone (DHA) and TPP.

The XuMP pathway has been introduced into *Saccharomyces cerevisiae*.^361^ While DAS is a key enzyme in natural C1 assimilation, it has not been extensively used in synthetic C1 assimilation pathways. However, other TPP-dependent enzymes have been explored in synthetic C1 assimilation due to their ability to perform Umpolung reactions of C1 compounds, which are mostly electrophilic. Notably, Umpolung allows direct coupling of two C1 building blocks. In recent years, this idea has gained considerable attention, and multiple enzymes have been engineered to directly link C1 units. The first of these was formolase (FLS),^358,362^ a benzaldehyde lyase which was engineered with the help of computational protein design. FLS condenses three molecules of formaldehyde to one molecule of DHA, making it the only known enzyme to catalyze a C1-to-C3 conversion. Mechanistically, it first performs an Umpolung of one formaldehyde molecule, which allows a nucleophilic attack on the second formaldehyde molecule. The resulting glycolaldehyde-TPP intermediate acts again as a nucleophile and attacks a third molecule of formaldehyde to produce DHA as the final product.^358^ As DHA can be incorporated into glycolysis via phosphorylation, formolase can be readily integrated into metabolism. Formate assimilation via formolase has been integrated into *E. coli* using the linear methanol assimilation pathway,^343^ the Half-Wood–Ljungdahl–Formolase (HWLS)^340^ pathway and the synergistic metabolism of glucose and formate (SMGF) pathway (Table 7).^342^ In addition, a cell-free system, the artificial starch anabolic pathway (ASAP),^255^ uses formolase in the conversion of methanol to starch. In another in vitro cascade, formolase was used to link two molecules of glycolaldehyde to produce the C4 sugar erythrulose.^315^ However, pathways utilizing FLS are strongly limited by the enzyme’s low efficiency. Additionally, in vivo realization of pathways involving formolase are expected to suffer from formaldehyde’s toxicity.

Higher reaction rates are achieved in TPP-dependent additions of C1 compounds to C2 molecules. For example, benzoylformate decarboxylase (BFD) was evolved toward the production of glycolaldehyde from two molecules of formaldehyde and subsequently enabled methanol assimilation in *E. coli* via the synthetic acetyl-CoA (SACA) pathway (Table 7).^341^ The same reaction, catalyzed by glyoxylate carboligase (GCL), was used to produce ethylene glycol in a short whole-cell biocatalytic cascade.^363^

Other examples of C1-C1 bond forming enzymes include members of the enzyme families 2-hydroxyl-CoA lyase (HACL) and oxalyl-CoA decarboxylase (OXC), which were shown to catalyze the reaction of formaldehyde with formyl-CoA.^339,360,364^ HACL was implemented in *E. coli* in the formyl-CoA elongation (FORCE) pathway for the conversion of methanol to glycolate (Table 7).^339^ Meanwhile, OXC was shown to catalyze C1-elongation of a range of aldehydes, yielding the CoA esters of chemicals like lactic or mandelic acid.^359,360^

### 5.8 Pyruvate Formate-lyase: Exploiting Reverse Reactions

Pyruvate formate-lyase (PFL) is not part of any natural C1 fixation pathway, but is involved in the anaerobic glucose metabolism of bacteria. In its reverse reaction, it was shown to enable growth of *E. coli* on formate and acetate.^365^ PFL is the only known enzyme to assimilate unactivated formate. It performs this challenging task by employing a radical mechanism. However, PFL is sensitive to oxygen. To reduce the enzyme’s exposure to oxygen, it was recently successfully encapsulated in a bacterial microcompartment to create a microaerobic environment under aerobic growth conditions.^366^

**Figure 19:**
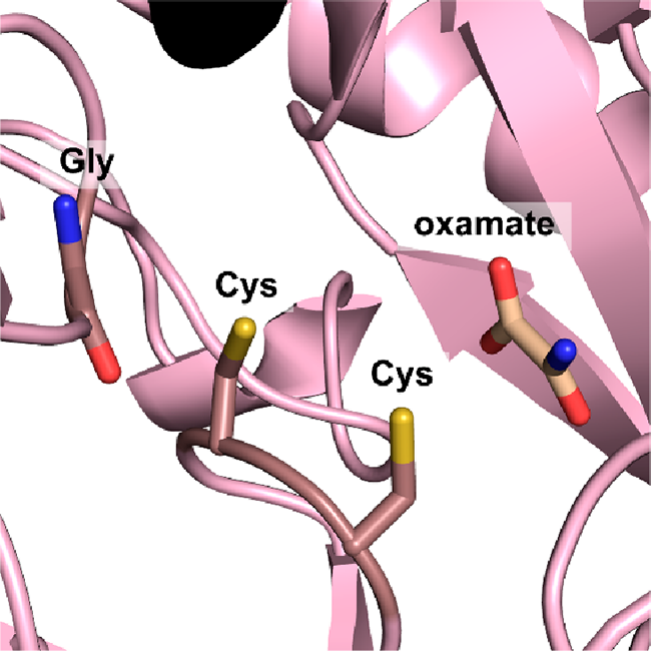
Crystal structure of *E. coli* PFL (PDB 3PFL).^367^ The relevant active site residues are shown as sticks. Oxamate (beige) is used as a substrate analogue in place of the natural substrate pyruvate.

**Scheme 29:**
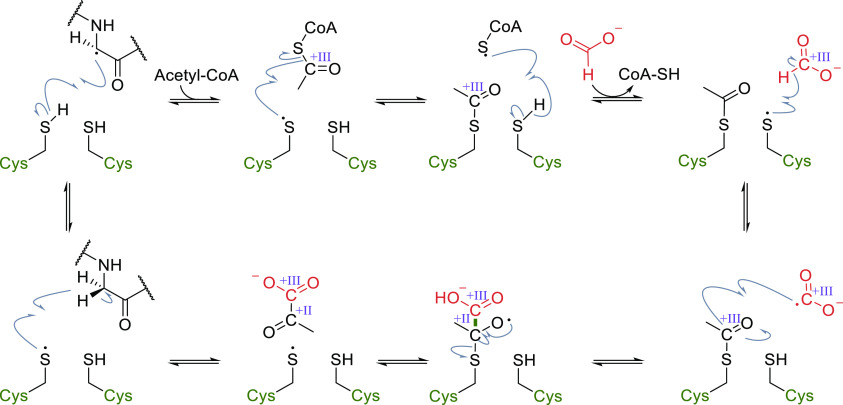
Radical Mechanism for the Condensation of Formate and Acetyl-CoA Forming Pyruvate, Catalyzed by PFL;^367^ The Oxidation State of Relevant Carbon Atoms Are Given in Roman Numerals

The radical mechanism of PFL is initiated by PFL-activating enzyme, generating a 5′-deoxyadenosine radical from *S*-adenosyl methionine. The radical is then transferred to an active site glycine of PFL (Figure 19). This glycyl radical abstracts a hydrogen atom from an adjacent cysteine residue, forming a cysteinyl radical. The radical subsequently reacts with acetyl-CoA, forming an enzyme bound acetyl-thioester and releasing a CoA radical, which is quenched by a second cysteine residue in the active site (Scheme 29). A hydrogen atom transfer from formate to this cysteinyl radical produces another radical species, which forms a bond with the enzyme-thioester, yielding a carboxyacetyl radical bound to the cysteine residue. Finally, pyruvate is released from the cysteine and the radical is transferred back to the initial glycine residue.^367^

## 6 Techno-economic Perspective

**Scheme 30:**
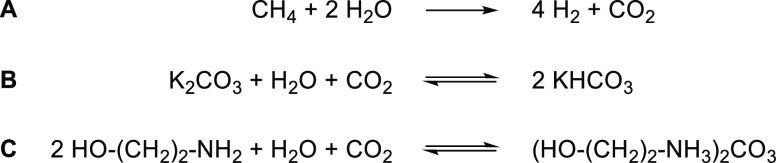
Industrial CO_2_ Production (A) CO_2_ is a byproduct of hydrogen production by steam reforming. The CO_2_ is removed from the product mixture by absorption in an aqueous solution of (B) potassium carbonate or (C) ethanolamine at high pressure and low temperature, and subsequently released by raising the temperature and lowering the pressure.

Today, the majority of the industrially used CO_2_ is generated as a side product of ammonia production. The hydrogen required for ammonia synthesis is obtained by steam methane reforming, resulting in 5.5 tons of CO_2_ for every ton of hydrogen produced (Scheme 30). The mixture of CO_2_ and hydrogen, containing approximately 18% CO_2_, is passed through a solution in which CO_2_ is absorbed with the help of either potassium carbonate or ethanolamine. The absorbed CO_2_ is released as concentrated gas upon heating the solution and subsequently liquified for storage and transport, if it is not directly used in a downstream process. Following CO_2_ release, the absorption solution must be cooled to restore its CO_2_-uptake capacity.^368^

CO_2_ capture from flue gases produced in fossil fuel-driven power plants and CO_2_ sequestration from the atmosphere operate with the same principle: selective CO_2_ absorption to a liquid or solid phase from a mixture of gases with CO_2_ concentrations between 0.04% (atmosphere) and 20% (flue gases) followed by CO_2_ release upon temperature increase. However, even considering state-of-the-art amine scrubbing technology, it is estimated that 20–30% of the power that is produced in a fossil-fuel driven power plant would be required to capture all the generated CO_2_, thereby reducing the overall efficiency.^369,370^ Capturing atmospheric CO_2_ is even more energy-intensive because absorbent materials must provide higher absorption enthalpies to efficiently capture the CO_2_ from the more dilute mixture and consequently require a proportionally higher energy input for subsequent release. In order to generate a significant reduction in atmospheric CO_2_ concentrations by way of carbon capture, the required energy must come from renewable sources.^371^ Once ample electricity produced by CO_2_-neutral processes is available, direct air capture of CO_2_ may become one of several technologies suitable to extract CO_2_ from the atmosphere.^372^ The availability of efficient processes to convert sequestered CO_2_ to industrial chemicals may increase the attractiveness of direct air CO_2_ capture.

Technologies that allow the direct use of scrubbed industrial off-gases with a CO_2_ content of up to 20% as carbon source are advantageous as enrichment and purification steps are not required. Consequently, carbon fixation reactions that can operate at low concentrations of dissolved CO_2_ or bicarbonate are techno-economically preferred. Such integrated CO_2_ capture and direct conversion processes are currently only possible with chemical reactions.^369^ The implication for biological processes is that the enzyme’s *K*_m_ value for dissolved CO_2_ or bicarbonate should be taken into consideration when designing CO_2_-utilizing biocatalysts or CO_2_ fixation pathways. Note that the *K*_m_ value for CO_2_ in plant RuBisCO homologues is on average slightly higher than the concentration of dissolved atmospheric CO_2_ in water under physiological conditions (14 μM at 400 pm).^373−375^ Therefore, when using feedstock gases with enriched CO_2_, RuBisCO enzymes would be easily substrate-saturated with respect to CO_2_. For other CO_2_-fixing enzymes such as reversed decarboxylases, the case is less clear because their *K*_m_ values for CO_2_ are not known. It would be worthwhile to determine these values to evaluate whether they are already saturated by the 6.8 mM dissolved CO_2_, resulting from using industrial exhaust gas mixtures with 20% CO_2_ content at ambient pressure, or whether a pure CO_2_ atmosphere would be required to efficiently drive the carboxylation.

The process parameters that must be met for a process to be commercially viable depend on the value of the product. For building the structural complexity of valuable active pharmaceutical ingredients (APIs), or other fine chemicals, biocatalytic reactions are particularly attractive because their innate chemo- and regioselectivity allows for shorter synthetic routes and a higher atom efficiency through the omission of activating reagents and protective groups.^376^ These advantages offset the typically higher catalyst and asset utilization costs caused by often longer reaction times and lower product concentrations compared to classical processes.^2^ Nevertheless, even for API-syntheses, product concentrations up to 100 g/L are the standard for biocatalytic reactions nowadays.^376^ It is well conceivable that with a highly active catalyst and proper reaction engineering, an enzymatic carboxylation step employing a reversed decarboxylase can contribute to the synthesis of an API or other chemicals. A promising example is the biocatalytic *ortho*-carboxylation of *meta*-aminophenol to *para*-aminosalicylic acid, which is a tuberculostatic agent using salicylic acid decarboxylase from *Trichosporon moniliiforme* (SAD_Tm; see also section 3.2.1 and Table 2); the latter was further improved by enzyme engineering (200 mM scale).^170^ Furthermore, the *ortho*-carboxylation of polyphenols such as resveratrol was used to enhance its polarity and water solubility, which are assumed to be beneficial for bioavailability.^160,158,169^

Overall, as biocatalytic reactions generally run at ambient conditions, biocatalysis can reduce a process carbon footprint, even if no carboxylation step is involved. Still, while biocatalysis is increasingly applied in industry, the number of large-scale processes is limited and therefore its impact on CO_2_ sequestration or the reduction of its emission is of minor impact on a global scale.

CO_2_ fixation on global scale hinges on the notoriously inefficient enzyme RuBisCO and is performed by algae and plants, which annually fix 10^14^ kg C.^377^ RuBisCO’s inefficiency results from its comparatively low *k*_cat_ and its poor discrimination between CO_2_ and O_2_. One hypothesis is that RuBisCO emerged during times in which the earth’s atmosphere contained very little oxygen. Once the oxygen levels had risen, the carbon-assimilation pathways involving RuBisCOs likely already had evolved, and fundamentally different molecular mechanisms could no longer evolve from scratch because they would be outcompeted by the established RuBisCO and the CBB. These arguments motivated and paved the way for the design of de novo, artificial carbon fixation pathways and their subsequent in vivo realization. Note that increasing the carbon fixation efficiency in crops even by only a few percent would have a huge impact on global CO_2_ fixation rates and also improve agricultural yields. Currently, 12 million km^2^ (3.1 billion acres) cropland are in use that capture together almost 5.5 × 10^12^ kg C annually.^378^ Although agricultural efficiency has already substantially increased in the past century (e.g., between 1961 and 2005 global land productivity increased by a factor of 2.4^379^), further developments are necessary to provide food for a growing global population to supply feedstocks for a renewable-based chemical production,^380^ and to contribute to reducing atmospheric CO_2_ concentrations through bioenergy with carbon capture and storage (BECCS).^381^ The introduction of artificial carbon fixation pathways in planta provides an exciting approach to further increase plant productivity. Although genetic engineering of plants is much more laborious than microorganisms, fundamental advances in engineering crop plants were achieved in the past few decades. While previously being limited to only one or two heterologously expressed genes (e.g., conferring tolerance to insects, diseases, or herbicides), the genetic engineering capabilities have strongly improved. Recently, a cassette containing 10 different genes constituting a metabolic pathway for the biosynthesis of polyunsaturated fatty acids and one additional gene conferring herbicide resistance were introduced into canola.^382^ The resulting canola variety has been approved for commercial use in the U.S., illustrating the technical and regulatory feasibility of introducing heterologous pathways with a size comparable to that of an average carbon fixation pathway into crop plants.

While the ultimate goal is the enhancement of crops for food and biofeedstock production, genetically accessible microalgae such as *Chlamydomonas reinhardtii* may be the primary choice for addressing fundamental questions and pathway optimization.^383^ For industrial production of biomass and chemicals, however, microalgae are challenging microorganisms because they grow slowly and to low cell densities. Their large-scale cultivation requires complicated reactor designs that provide high surface-to-volume ratios to maximize light influx while allowing good mass transfer to supply CO_2_ and nutrients to the cells. In the very long-term, cultivation of genetically engineered microalgae in open ponds is conceivable, with saltwater lagoons being particularly attractive from a sustainability perspective. However, such open cultivation is not reconcilable with current policies on containment of genetically modified microorganisms.^384^

With light-driven carbon fixation being unattainable at large scale in industrial reactors in the short term, the required energy for CO_2_ and water splitting could come from renewably generated electric energy that is provided to microorganisms or cell-free systems through electrodes. Such reactors would similarly need an extremely high surface-to-volume ratio because the range for electron transfer in an aqueous environment is so short that only the first few layers of cells colonizing an electrode could be supplied with the electrons required for CO_2_ reduction.^325,385^ Such complex reactors will not only be capital intensive but also expensive to operate, as they are difficult to clean and maintain and have poor volumetric productivities. These limitations could be overcome by separating the CO_2_ reduction step from the build-up of larger molecules.^312^ CO_2_ reduction to formic acid, formaldehyde, or methanol could be driven by chemical catalysis operating at high efficiency and volumetric productivity. These water-soluble intermediates could then be fed as carbon source to microorganisms in conventional aerobic or anaerobic fermentation setups and converted to more complex chemicals.

**Scheme 31:**
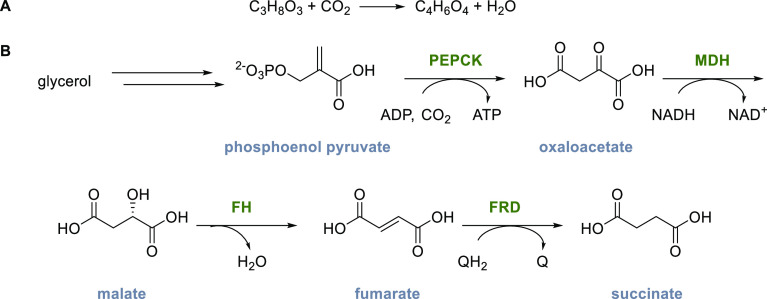
Fermentative Production of Succinic Acid from Glycerol and CO_2_ (A) Overall reaction equation. (B) Metabolic pathway of *Mannheimia succiniciproducens*.^388^ Glycerol is converted to phosphoenolpyruvate, PEP, via dihydroxyacetone. PEP, carboxykinase; PEPCK, carboxylates PEP to oxaloacetate and transfers the phosphate group to ADP to produce ATP. Malate dehydrogenase, MDH, reduces the α-keto-acid to the α-hydroxy-acid malate, which is dehydrated by fumarate hydratase, FH, to fumarate. Fumarate reductase, FRD, reduces fumarate to succinate, using quinol as hydrogen donor.

Including CO_2_ fixation steps into biosynthetic pathways can contribute to the sustainability of microbial fermentations, even beyond usage of renewable feedstocks.^386^ A prominent example is the fermentative production of succinic acid, an important building block for the synthesis of polyesters (Scheme 31). While the current global market size for succinic acid is relatively small, with 15 kt annually, it may grow substantially when the demand for biobased polymer building blocks increases.^386,387^ The key step of the most efficient metabolic route, the reductive TCA route, is carboxylation of phosphoenolpyruvate (PEP) by either PEP carboxylase or PEP carboxykinase, resulting in one equivalent of CO_2_ fixed per molecule succinic acid produced.^388^ The overall reaction is redox neutral when using glycerol as carbon source,^389^ but even if a process is not redox-neutral, a CO_2_ fixation step can contribute to a net CO_2_ consumption in fermentation.

In summary, enzymatic CO_2_ fixation steps can contribute already in the short term to improving the sustainability profile of chemicals production. The contribution can be especially favorable if the process does not require purified CO_2_, and the specific energy input needed to drive the fixation is low. The latter is particularly true for redox-neutral processes such as succinic acid production, which unfortunately are not common. In all other cases, the large amounts of energy required for attaining any of the reduced carbon oxidation states must not only be generated but also supplied at a rate that matches the rate of CO_2_ fixation. Sunlight is ubiquitous and free, but the rate of energy transfer through photosynthesis is too slow to achieve the conversion rates expected for high density cell cultures in conventional industrial bioreactors. Conversely, plants have matched the rate of light harvesting to CO_2_ fixation and subsequent reduction, even though CO_2_ fixation remains limiting. Whether the carbon sequestration capacity of crop plants can be increased by the introduction of additional CO_2_ fixation cycles remains to be seen, but given the recent advances in constructing such pathways and the expanding toolbox for genetic modification of crop plants, progress can be expected. As an alternative to light, the energy required for CO_2_ reduction can be supplied as electrical energy through electrodes. However, the distances required for electron transfer are so short that cells would need to attach directly to the electrodes, requiring intricate reactor designs that are difficult to upscale to the dimensions necessary for generating a global impact. Here, multistep processes consisting of electrochemical CO_2_ conversion of C1 molecules, followed by fermentative conversion, are much more conceivable in the near future. The gas fermentative production of ethanol from synthesis gas, a mixture of CO, CO_2_, H_2_, and N_2_, by acetogenic *Clostridium* cultures highlights the potential of utilizing microbes to build up larger molecules from C1 compounds, in this case CO and CO_2_, at industrial scale.^390^

## 7 Outlook and Opportunities

The previous sections have highlighted that CO_2_ is not only a waste product harmful to the climate. It can also serve as an enzymatic substrate for the production of chemicals and is even required for the growth of many organisms, including plants. Although recent advances have shown that a range of products can be enzymatically synthesized from CO_2_, some inherent limitations remain. For example, due to the low potential energy of CO_2_, it is often necessary to employ high concentrations of CO_2_ (or bicarbonate) to shift the equilibrium to the product side and achieve acceptable yields in carboxylation reactions involving only a single enzyme. Although implementation of product removal techniques, as well as coupling the carboxylation steps to thermodynamically favorable downstream reactions, have shown to significantly improve reaction efficiencies, further improvements in this area are desirable.

Carboxylation reactions are challenging, and therefore only few reactions directly employ CO_2_ as starting material in organic synthesis. While the carboxylation reaction scope of enzymes is broader, still only a small range of compounds can be biosynthesized from CO_2_ directly. Besides broadening the substrate scope of existing enzymes by protein engineering, identification of new carbon-fixing enzymes is an attractive way to tackle this problem. Driven by the rapid increase in available sequence data and the development of ever more efficient genome mining techniques, the discovery of novel carbon-fixing enzymes can be anticipated. Another approach is repurposing existing enzymes to reduce CO_2_. The nitrogen gas-reducing enzyme nitrogenase is particularly interesting in this regard as it has been shown to accept both CO and CO_2_ as substrates to produce reduced hydrocarbons such as methane, ethylene, and propane.^391−396^ However, hydrocarbon production by nitrogenase is currently limited by low turnover numbers. Furthermore, the enzyme’s oxygen sensitivity and its dependence on complex cofactor maturation systems has further complicated the study of nitrogenases. An alternative to using natural systems is complementing enzymes with chemically-synthesized cofactors. Such systems have produced promising results. For example, by incorporation of photosensitizers, proteins with extremely high reducing power have been generated. The reduction potential of these photosensitizer proteins could be tuned by enzyme engineering, which allowed production of CO^396^ and formate^397^ from CO_2_ using NADPH as electron donor.

It is also possible to increase the palette of products accessible from CO_2_ by using carboxylation products as substrates in downstream reactions. Nature already very successfully applies this principle, as almost all biomass can be produced from only three products of naturally occurring carbon fixation cycles: acetyl-CoA, pyruvate, or glyceraldehyde-3-phosphate. The same idea can be applied to synthetic CO_2_ fixation cascades for sustainable production of chemicals without using any commodity chemicals derived from fossil feedstocks. A proof-of-principle study that such systems can function has been established for the synthesis of terpenes and polyketides from CO_2_.^314^ However, much more work needs to be done to improve this system beyond the proof-of-principle phase. One important challenge will be to increase the longevity and robustness of synthetic in vitro systems, e.g., by balancing fluxes and cofactor regeneration. Furthermore, higher flux through pathways is expected if it were possible to selectively remove the products of a given transformation, e.g., within engineered physical structures. Multicompartment engineered physical structures could mimic natural photosynthesis, in which fixed carbon is constantly pumped out of the chloroplast to create a strong source–sink relationship. The use of structures with selective transport capabilities would allow scientists to optimize reaction conditions between different compartments and implement different maintenance regimes, some of which would be impossible in living cells.

The ultimate challenge of synthetic biology is building novel living systems, often called synthetic cells. The central part of every living organism is carbon metabolism. In this regard, the in vivo implementation of artificial carbon fixation pathways is highly interesting as it would lay the foundation for artificial metabolism with no precedent in nature. At the same time, in vivo implementation of carbon fixation pathways into microorganisms is an interesting approach to validate their performance. Once the most efficient pathways have been identified, crops will be particularly attractive targets for engineering. As currently carbon fixation is a limiting factor in plant growth, improvements of carbon fixation efficiency are predicted to directly translate to increased photosynthetic yields. Opportunities thus arise in creating plants that feature new-to-nature CO_2_-fixing capabilities either in the context of improving CO_2_ fixation directly or by improving photorespiratory metabolism. Precedent shows that even introducing carbon-neutral photorespiratory bypasses can already stimulate plant growth in the field,^398^ which strongly incentivizes continuation of such efforts to expand the capabilities of plant metabolism beyond the constraints of natural evolution. With all the recent progress and enthusiasm around CO_2_ sequestration and fixation technologies, it should not be forgotten that the technologies discussed in this review will only provide an impact in the future. However, slowing down the rise in atmospheric greenhouse gas concentrations warrants immediate action and we must address the issue with today’s toolbox focusing on reducing global carbon emissions.

## Supporting Information Available

Extended version of Table 5 (XLSX)

## Abbreviations

2,3-DHBH: 2,3-dihydroxybenzoic acid decarboxylase
2KFOR: 2-ketogluterate:ferredoxin oxidoreductase
3HP/4HB: 3-hydroxypropionate/4-hydroxybutyrate
3-PG: 3-phosphoglycerate
5,10 mTHF: 5,10-methylene tetrahydrofolate
ACC: acetyl-CoA carboxylase
ACPCC: acetyl-CoA/propionyl-CoA carboxylase
API: active pharmaceutical ingredient
ASAP: artificial starch anabolic pathway
BC: biotin carboxylase
BCCP: biotin carboxyl carrier protein
BFD: benzoylformate decarboxylase
CA: carbonic anhydrase
CABP: 2-carboxyarabinitol biphosphate
CAM: Crassulacean acid metabolism
CBB: Calvin–Benson–Bassham
CCM: carbon concentrating mechanism
CCR: crotonyl-CoA carboxylase/reductase
CETCH: crotonyl-coenzyme A (CoA)/ethylmalonyl-CoA/hydroxybutyryl-CoA
CODH: CO dehydrogenase
CT: carboxytransferase
DAS: dihydroxyacetone synthase
DFT: density functional theory
DHA: dihydroxyacetone
FDH: formate dehydrogenase
FLS: formolase
FMFDH: formylmethanofuran dehydrogenase
FORCE: formyl-CoA elongation
GCL: glyoxylate carboligase
GCS: glycine cleavage system
GED: Gnd–Entner–Doudoroff
H4MPT: tetrahydromethanopterinin
HACL: 2-hydroxyl-CoA lyase
HPS: 3-hexulose-6-phosphate synthase
HWLS: Half–Wood–Ljungdal–Formolase
IDC: iso-orotate decarboxylase
IDH: isocitrate dehydrogenase
ISPR: in situ product removal
LigW: 5-carboxyvanillate decarboxylase
mcd: methylsuccinyl-CoA dehydrogenase
MDF: max–min driving force
MIMS: membrane inlet mass spectrometry
OXC: oxalyl-CoA decarboxylase
PEP: posphoenolpyruvate
PEPC: phosphoenolpyruvate carboxylase
PFL: pyruvate formate-lyase
PFOR: pyruvate:ferredoxin oxidoreductase
PLP: pyridoxal-5̀-phosphate
POAP: PYC-OAH-ACS-PFOR
prFMN: prenylated flavin mononucleotide
PRI: phosphoriboseisomerase
PRK: phosphoribulokinase
rGlycine: reductive glycine
rGPS-MCG: reductive glyoxylate and pyruvate synthesis cycle and malyl-CoA-glycerate pathway
RLP: RuBisCO-like enzymes
rTCA: reverse tricarboxylic acid cycle
RuBisCO: ribulose-1,5-bisphosphate carboxylase/oxygenase
RuBP: ribulose-1,5-phosphate
RuMP: ribulose monophosphate
SACA: synthetic acetyl-CoA
SAD: salicylic acid decarboxylase
SHMT: serine hydroxymethyltransferase
SMGF: synergistic metabolism of glucose and formate
TaCo: tartronyl-CoA
TCA: tricarboxylic acid
THF: tetrahydrofolate
TPP: thiamine pyrophosphate
WL: Wood Ljungdahl
XuMP: xylulose monophosphate
